# Conserved genes in a path from commensalism to pathogenicity: comparative phylogenetic profiles of *Staphylococcus epidermidis *RP62A and ATCC12228

**DOI:** 10.1186/1471-2164-7-112

**Published:** 2006-05-10

**Authors:** Wu Wei, ZhiWei Cao, Yu-Li Zhu, XiaoJing Wang, GuoHui Ding, Hao Xu, PeiLin Jia, Di Qu, Antoine Danchin, YiXue Li

**Affiliations:** 1 Bioinformatics Center, Shanghai Institutes for Biological Sciences, Chinese Academy of Sciences; Graduate School of the Chinese Academy of Sciences, 320 YueYang Road, Shanghai, China; 2 Shanghai Center for Bioinformation Technology, 100 Qinzhou Road, Shanghai, China; 3 Department of Medical Molecular Virology, Institute of Medical microbiology, Shanghai Medical School of Fudan University, 138 YiXue Yuan Road, Shanghai, China; 4 Genetics of Bacterial Genomes/URA 2171 CNRS, Institut Pasteur, 28 rue du Docteur Roux, 75724 Paris Cedex 15, France

## Abstract

**Background:**

*Staphylococcus epidermidis*, long regarded as an innocuous commensal bacterium of the human skin, is the most frequent cause of nosocomial infections associated with implanted medical devices. This conditional pathogen provides a model of choice to study genome landmarks correlated with the transition between commensalism and pathogenicity. Traditional investigations stress differences in gene content. We focused on conserved genes that have accumulated small mutation differences during the transition.

**Results:**

A comparison of strain ATCC12228, a non-biofilm forming, non-infection associated strain and strain RP62A, a methicillin-resistant biofilm clinical isolate, revealed consistent variation, mostly single-nucleotide polymorphisms (SNPs), in orthologous genes in addition to the previously investigated global changes in gene clusters. This polymorphism, scattered throughout the genome, may reveal genes that contribute to adaptation of the bacteria to different environmental stimuli, allowing them to shift from commensalism to pathogenicity. SNPs were detected in 931 pairs of orthologs with identical gene length, accounting for approximately 45% of the total pairs of orthologs. Assuming that non-synonymous mutations would mark recent evolution, and hence be associated to the onset of the pathogenic process, analysis of ratios of non-synonymous SNPs vs synonymous SNPs suggested hypotheses about possible pathogenicity determinants. The N/S ratios for virulence factors and surface proteins differed significantly from that of average SNPs. Of those gene pairs, 40 showed a disproportionate distribution of dN vs dS. Among those, the presence of the gene encoding methionine sulfoxide reductase suggested a possible involvement of reactive oxygen species. This led us to uncover that the infection associated strain was significantly more resistant to hydrogen peroxide and paraquat than the environmental strain. Some 16 genes of the list were of unknown function. We could suggest however that they were likely to belong to surface proteins or considered in priority as important for pathogenicity.

**Conclusion:**

Our study proposed a novel approach to identify genes involved in pathogenic processes and provided some insight about the molecular mechanisms leading a commensal inhabitant to become an invasive pathogen.

## Background

Emerging diseases have been a matter of great concern recently: the sudden appearance of SARS and the recent spread of bird's flu raise the question of how microbes evolve into pathogens to human not only an important but also an urgent question to answer. Most microbes have a benign symbiotic relationship with humans and only cause infections to healthy individuals under limited conditions. *Staphylococcus epidermidis *is such an opportunistic pathogen which is a common member of the normal flora of our skin and mucous membranes. But on certain occasions, the presence of *S. epidermidis *as a contaminant of medical devices, or breach of the skin by trauma or inoculation needles, makes it emerge as a causative agent of infections [1]. As biomedical devices are increasingly used in medical practice, a major complication due to *S. epidermidis *infection when using these devices is affecting several millions of patients worldwide each year [2].

The pathogenic process of foreign-body-associated infections with *S. epidermidis *is characterized by the ability of this species to colonize polymer surfaces by the formation of multilayered cell clusters, which are enveloped and protected by an amorphous slimy material to form a biofilm [3-5]. Most genetic and biochemical evidence has shown that polysaccharide intercellular adhesion (PIA) production [6], mediated by the *icaABCD *operon [7-9], is crucial in biofilm fomation. *S. epidermidis *does not produce components that are easily recognized as virulence factors, such as toxins or aggressive degradative exoenzymes [10]. Genetic manipulation of *S. epidermidis *has been difficult so far, limiting efforts that would elucidate the molecular basic of its pathogenicity. The analysis of bacterial genome sequences provides us with an alternative way to investigate some of the constraints that might pave the way from commensalism to pathogenicity in this species.

*Staphylococcus epidermidis *is one of the most commonly isolated bacterial pathogens in hospitals because of its large numbers and ubiquitous distribution. Environmental *S. epidermidis *strains differ significantly in their invasive and ability to form biofilm. Two complete genomes of *S. epidermidis *strains have been sequenced, one is ATCC 12228, a non-biofilm forming/non-infection associated strain, and another one is RP62A (also called ATCC35984), an infectious and biofilm forming strain [11,12]. Both strains are highly similar in genome sequence, and we tried to see whether some particular features might be correlated with the surviving ability of the organisms in certain environment, allowing us to uncover processes that might be important for the transition between a commensal life style, and a pathogenic behavior. In previous comparative genomics studies, the most obvious differences between these genome pairs revealed the loss or gain of large DNA segments [13]. Further comparative studies of *S. epidermidis *with other staphylococcal species indicated that the majority of the genes unique to a given strain or species is also related to the presence or absence of prophages and genomic islands. In the absence of other criteria to define virulence, some of these genes were proposed to be the main causes of pathogenicity and virulence [12]. However, acquisition of those genes has repeatedly been reported to be insufficient to trigger a pathogenic response [14]. For example, it has been discovered that the ATCC12228 and RP62A strains share most of the possible pathogenic factors. As a case in point, the *cap *operon encoding the polyglutamate capsule and being recognized as a major virulence factor in *Bacillus anthracis*, has been found to be integrated into the genomes of both *S. epidermidis *RP62A and ATCC12228, while the latter is a non-infectious strain [12,15]. In addition to gross differences displayed as genomic islands and unique genes, Single Nucleotide Polymorphisms (SNPs) have also been found to widely exist in the chromosomes of the two strains, especially in some genes with cell envelope functions [12]. Therefore, pathogenicity appears to be a complex phenomenon which could be accounted for not only by large genetic differences, but also by small variations in gene contents. This prompted us to perform a detailed SNPs comparative analysis between *S. epidermidis *ATCC12228 and RP62A, complementing the previous approaches used to differentiate pathogenic strains from non-pathogenic ones [16-18]. Assuming that pathogens have evolved recently [19], the amount of small genetic variations that would be accumulated in the genome sequences might not have had time to undergo purifying selection, thus revealing some of the genes that are important for pathogenic processes. Analyzing those variations, mostly SNPs, we expected to find genes bearing landmarks of recent evolution, and which may contribute to the pathogenesis of the bacterium. In this paper, we focused on the systematic SNP analysis between a pair of infectious and non-infectious strains of *S. epidermidis*, in particular by comparing synonymous and non-synonymous mutations in orthologous gene pairs. The genes displaying higher dN/dS rate, including genes of unknown function, were analyzed in relation with function possibly associated to pathogenicity.

## Results

### Small variations between the genomes of two *S. epidermidis *strains

The *S. epidermidis *RP62A and ATCC12228 chromosomes are 2,616,530 bp and 2,499,279 bp long, respectively [11,12]. Beside large segments coding for genes present in one genome and absent in the other, many small scale genetic variations which affected an individual gene or a small numbers of genes (< 10 CDSs) were dispersed throughout nearly all of both chromosomes, revealing extensive divergence between both species. The comparison of the insertions and deletions (indels) differentiating the two genome sequences showed that such events generally involved small-scale variations (see Additional file 1). In addition to indels, a total of 10,297 SNPs were found in the genome of *S. epidermidis *ATCC12228 when compared to that of strain RP62A [12]. These small-scale variations, mostly SNPs, might contribute to the different phenotypes of the two strains. We therefore focused on the SNPs of the orthologous genes to explore how they could contribute to adaptation of the bacteria to various environmental stimuli allowing them to shift from commensalism to pathogenicity.

The *S. epidermidis *RP62A and ATCC12228 chromosomes contain 2,494 and 2,419 predicted protein-coding sequences (CDSs), respectively [11,12]. Of the 2,419 predicted genes encoded by the ATCC12228 chromosome, 2,053 (85%) have an ortholog in RP62A (Table 1). Most of those genes are almost identical in sequence between both organisms. SNPs were detected in 931 pairs of orthologous genes with identical gene length, accounting for approximately 45% of the orthologous genes set (see Additional file 2). Among those orthologs with SNPs and of identical length, 118 pairs were identical to each other at the amino acid level, while the other 813 pairs (87%) displayed some changes in their amino acid sequence. In addition, more complex insertion/deletion events and related variations were observed in 263 pairs of orthologs.

### Pathogenicity-related genes and highly expressed proteins genes evolve differently

Pathogenicity in warm blooded vertebrates is expected to have arisen recently (at the living organisms evolution time scale). Mutations occurring during that period should have started more or less randomly, while selection pressure would have retained only some of them. This prompted us to measure the contribution of synonymous mutations (presumably not changing the existing fitness of the proteins) as compared to non-synonymous mutations. In order to assess the effect of non-synonymous mutations upon the intraspecies differentiation of the two *S. epidermidis *strains, we calculated the ratios of total non-synonymous SNPs (N) vs total synonymous SNPs (S) of all genes, with some emphasis on virulence factors, surface proteins [12] and translation, ribosomal structure and biogenesis-related proteins (see Additional file 3 and file 4). This comparison would be a first proof of concept, while we were trying to uncover genes' illuminating or unexpected functions that would, in this way, suggest a participation in the evolution of pathogenicity. The N/S ratios for virulence factors and surface proteins differed significantly from that of all SNPs. In contrast, translation-related proteins also showed a significant bias when compared to total SNPs, but displayed mostly synonymous substitutions, indicative of a selective stabilization process leading to purifying selection(Table 2). This analysis shows that virulence factors and surface proteins evolved quickly, in parallel with the pathogenicity environment. That this is significant is emphasized by the observation that translation, ribosomal structure and biogenesis-related proteins, which are submitted to considerable structural and functional constraints and are highly expressed, evolved slowly (there is hardly any change in protein sequence, while the gene sequence has evolved) [20].

**Figure 1:**
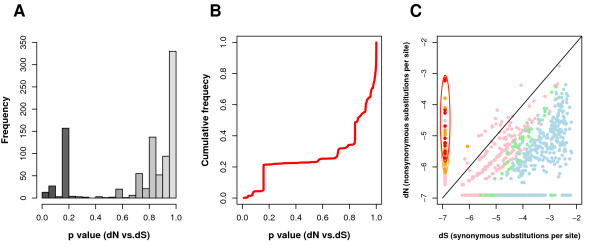
**Distribution of dN vs dS of SNPs pairs of two *S. epidermidis *strains**. The distribution of dN vs dS mutations was represented according to the p-value calculated from the One-tail Z (or *t*_∞_) test. Figure 1A represents the numbers of SNPs pairs falling into the different confidence level classes. Figure 1B shows the cumulative probability of all SNPs pairs. Figure 1C plots the distribution of dN/dS of all SNPs pairs. All plots are color-coded according to different intervals: red for [0, 0.05), orange for [0.05, 0.1), green for (0.9, 0.95], blue for (0.95, 1], pink for others.

To identify groups of SNPs genes differing in their evolution pattern, the dS and dN of each SNPs pairs of the two *S. epidermidis *strains were calculated and the distribution of individual SNPs pairs were analyzed using the one-tail Z (or *t*∞) test (see Additional file 5). Among 931 pairs of orthologs with SNPs, 213 (23%) pairs had a majority of non-synonymous substitutions (dN > dS), while the remaining 718 (77%) pairs had a majority of synonymous mutations (Figure 1A and 1B). This suggested that the majority of orthologs with SNPs suffer considerable selection constraints, indicative of adaptation to similar environments for both strains. The others, in contrast, may contribute to the bacterial pathogenesis process during the establishment of infection. In those synonymous pairs, we therefore restricted our analysis to the confidence interval of p < 0.1 of dS/dN to find what kinds of genes significantly suffered purifying selection pressures. As mentioned above, many genes of the translation machinery and energy production were found in that category. It is worth noticing that genes involved in the translation machinery, such as ribosomal proteins and tRNA methyltransferases, are expected to be expressed at a high level in many transcriptome studies [21].

### Genes potentially involved in pathogenicity

A total of 40 genes showed a significantly disproportionate distribution of dN vs dS with p-value < 0.10 (Figure 1A and Table 3). In standard statistical analyses, p < 0.05 is the level traditionally considered significant; however, if we represented the relative amount of those genes, the plot distribution of dN/dS (Figure 1C) shows a continuum that is worth exploring and may be relevant as providing putative signatures of adaptation to pathogenicity. Because the organisms we have chosen are extremely similar, leading to very few differences between the two genomes, the advantage of using a stringent statistical constraint for discarding false positives will put aside false negatives under conditions where we need to explore as many paths as possible. Indeed, from the figure we can see that 213 (23%) genes of the 931 SNP ortholog pairs are under positive selection (dN/dS > 1), consistent with the observation of other works [22] and strongly suggesting that most, if not all of the retained non-synonymous mutations are significant. Furthermore, the accumulation of mutations in some genes can be the result of several independent processes, with some submitted to positive selection for variation, while others would only be affected by drift, precluding, at this stage, a refined statistical analysis. We therefore decided for a compromise, and chose a slightly larger sample, still significant at the p < 0.10 level (orange area), in order to see whether the genes in that category pointed at particular functions non randomly, which would indicate that they are indeed significant. This approach is meant to propose genes as candidates for further study of pathogenicity, and a small number of false positives should not hinder further research in the domain, while false negatives would eliminate important candidates. Interestingly, the genes thus identified included phenol-soluble modulin (PSM) family peptides (SE0847), a Clp protease (SE0674), as well as genes involved in osmoprotection. Finally, 16 of the non-synonymous significant genes were of unknown function. Careful analysis of these genes revealed however that they were likely to belong to functions important for pathogenicity, as indicated in Table 3. Several of those genes were counterparts of proteins generally conserved in Firmicutes (YvcD(SE0546), YheB(SE1527), YqfN(SE1247), YlaF(SE0806), YkyA(SE0790), YfmM(SE2128), YhfK(SE1745) and YqgE(SE1448)) and sometimes in larger Bacterial clades (SE1986). YvcD is a TPR-repeat protein, which might interact with nucleic acids; YkyA has a lipoprotein signal, and is similar to cell wall binding proteins; YqgE(SE1448) is similar to MultiDrug Efflux proteins and finally YfmM is likely to code for polyphosphate-AMP phosphotransferase. The very fact that they belong to this class of non synonymous SNPs makes them interesting candidates for further studies of pathogenicity.

Although the sequencing technique is quite accurate, the error frequency in a finished sequence is thought to be one error (frameshift or base substitution) in 10^3 ^to 10^5 ^bases [23]. To confirm the SNP sites identified in this study, we chose the top 13 pairs of mostly non-synonymous substitutions proteins and carried out PCR experiments on their cognate gene to validate the corresponding sequences. Sequences of the PCR products confirmed all the SNP sites we identified from the orthologs analysis.

**Figure 2:**
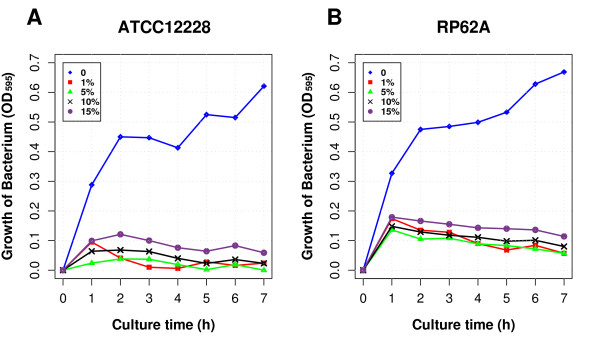
**Sensitivity to H_2_O_2 _of the two *S. epidermidis *strains**. Figure 2A displays the growth curve of *S. epidermidis *ATCC12228 and 2B, that of RP62A. The growth curves were plotted following the OD_595 _measured every one hour. Overnight bacterial cultures (OD_595 _approximately 1.2) were added to fresh TSB (Tryptone Soya Broth), and H_2_O_2 _at different concentrations (0, 1%, 5%, 10% and 15%) were added at the same time. The culture density was monitored at OD_595 _followed every one hour.

**Figure 3:**
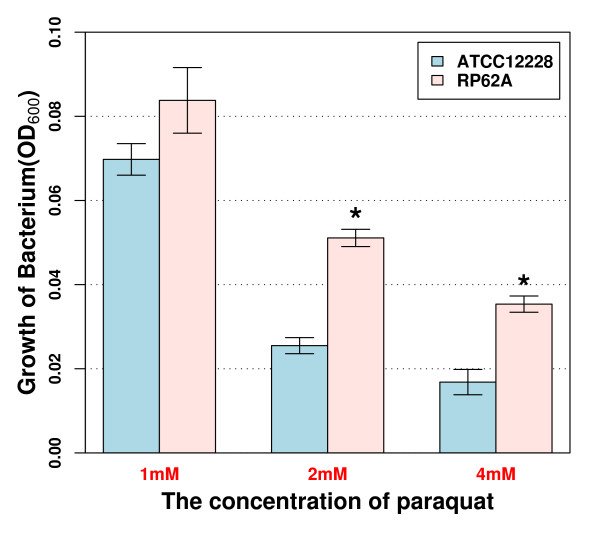
**Sensitivity to paraquat of two *S. epidermidis *strains**. The bacteria were cultured in MH broth (OXOID) with different concentration of paraquat (from 1 mM to 8 mM) and incubated at 35°C for 16–20 h. The MIC was defined as the lowest concentration of paraquat giving complete inhibition of visible growth. The growth of bacteria incubated with lower concentration paraquat than MIC was measured by OD_600_. The bar graph represents the average of three independent experiments, with standard deviations denoted by the error bars. *, *P *< 0.01.

Two main groups were observed in the cluster of 40 orthologous pairs made of proteins with mostly non-synonymous substitutions: surface proteins, which are likely under pressure to escape the host immune system, and other genes that should be considered in priority as important for pathogenicity. Two conserved hypothetical proteins (SE0265 and SE0378) predicted to localize in the extracellular medium and several transmembrane proteins, such as transporter family proteins, belonged to the first group. YkyA(SE0790), which contains a lipoprotein signal and a hydrolase domain, might also be recognized by the host immune defense. In the second group, several proteins involved in lipid metabolism, likely to be important for *S. epidermidis *multiplying on skin, apparently evolved faster in the pathogen. Genes encoding fosfomycin resistance protein FofB and beta-lactamase detected in this group also may have evolved fast to benefit bacteria trying to survive in their host. Genes involved in the formation of biofilms and osmoprotection were also found in this group. PSMs belong to the class of surfactant peptides with putative biofilm-inhibitory properties. Repression of expression of PSMs in the biofilm stage enables bacterial cells to adhere together and to evade the host immune system. Several gene products were involved in DNA recombination and repair (such as SE1170, SE1302 and SE1828) suggesting adaptation to some chemical stress. A gene (coding for methionine sulfoxide reductase, SE1042) involved in repair of Reactive Oxygen Species (ROS) damage, a process supposed to be significantly expressed upon infection [24], also suffered a high mutation rate. This prompted us to study the specific involvement of this process in the establishment of pathogenicity. Although the exact in vivo process of methionine oxygenation is not well established, it is supposed to be derived from ROS, in particular from reactions producing superoxide or H_2_O_2 _[25]. In an attempt to explore whether this prediction could be substantiated, both strains were challenged with increasing paraquat and H_2_O_2 _concentrations: interestingly, we observed that *S. epidermidis *ATCC12228 is indeed more sensitive to both paraquat [26] and H_2_O_2 _than *S. epidermidis *RP62A, as predicted (Figure 2 and 3, see Additional file 6 and Additional file 7).

Finally, genes are often grouped in operons, forming functionally consistent transcription units. If our hypothesis for the detection of genes important for pathogenesis is correct, then we should expect that a mutation bias would generally span entire operons when the corresponding genes participate to the process of pathogenicity. We found that genes making potential operons often displayed a collective increase in non-synonymous mutations (see Additional file 5). This is the case, for example, of *potA *(*se0797*) and *potB *(*se0798*), which code for the spermidine/putrescine ABC transporter.

## Discussion

While an important topic for human health, pathogenicity is not the most prevalent development process for living organisms. Often, this particular way of life happens as a new ecological niche (a possible host) is colonized by organisms that were previously indifferent or simple commensals. Previous work concluded, in general, that genome segments, often named pathogenicity islands, were the landmarks of pathogenic processes [27]. These islands are generally supposed to be the result of Horizontal Gene Transfer (HGT). HGT is a general process of gene acquisition by bacteria, where it may be associated to control of the background mutation level [28]. In Firmicutes, HGT is often the result of phage infection [29]. Bacteriophages can subsequently remain in the genome as functional (SPbeta in *B. subtilis*, for example) or more or less defective prophages (PBX, Skin, and similar elements). In the case of *S. epidermidis*, this type of analysis led Gill et al. to conclude that HGT was the major contribution to pathogenicity in the pathogenic strain they analyzed [12]. However, acquisition of virulence genes has repeatedly been found to be insufficient to trigger a pathogenic response. As a case in point, the *cap *operon, encoding the presumably virulence-associated polyglutamate capsule is present in both the commensal and pathogenic *S. epidermidis *strains [15]. The absence/presence of the *ica *operon that produces a biofilm exopolysaccharide was taken as the clearest genetic difference contributing to the different phenotype of two *S. epidermidis *strains [8,9]. However, the prevalence of *icaADBC *in commensal strains did not differ from that in invasive strains, indicating that other factors should been involved in pathogenesis [14]. All this indicates that important information about the development of pathogenic processes has been overlooked by the standard approaches focusing on HGT analysis. The small-scale variations which pepper the chromosomes of the commensal and pathogenic strains were not explored in detail. In order to gain further insight into the pathogenicity process, we studied the Single Nucleotide Polymorphisms (SNPs) between the orthologous gene pairs of the pathogenic and the commensal *S. epidermidis *strains of interest.

SNP analysis rests obviously on the quality of the genome sequences determination. The error frequency in a finished sequence has rarely been precisely measured. A common belief is that anywhere between one error in 10^3 ^to 10^5 ^bases, depending on the project. In order to evaluate the impact of sequencing errors on our analysis, approximately 23 kb of DNA sequence was re-sequenced after PCR amplification of 13 pairs of orthologs from both strains. 11 new SNP sites (5 and 6 in strains ATCC12228 and RP62A, respectively) were retrieved when the new sequences were compared to published genome sequences. If we take those new sites as sequencing errors, and not PCR errors or recent mutations, then the average error frequency is 4.8 errors in 10^4 ^bases. This shows that, while we may have missed some interesting genes, our approach is validated with the present available quality of genome sequences.

Analysis of the ratios of total non-synonymous SNPs vs total synonymous SNPs showed that N/S ratios for virulence factors and surface proteins differed significantly from that of total SNPs. While random mutations and evolutionary drift should not favor one type of mutation over the other one, genes related to pathogenic processes may have evolved recently due to positive selection to fit the pathogenic conditions [30]. Their evolution could be compared to that of genes belonging to translation, ribosomal structure and biogenesis (labeled according to the COG classification), which are submitted to purifying selection because of the general optimization of the translation process in the course of evolution. As expected, the latter were mostly submitted to synonymous mutations. This is remarkable and consistent with the interpretation of Drummond and colleagues who argued that highly expressed proteins evolve slowly because of the severe selective pressure on highly expressed proteins to avoid misfolding even when they are mistranslated [20]. This interpretation further supports the hypothesis that non synonymous mutations may be the landmark of proteins involved in pathogenic processes.

The percentages of identical vs. non-identical genes among the orthologs of those groups (virulence factors, surface proteins, translation-related genes and total genes) were also compared (see Additional file 9). Interestingly, the ratio of non-identical vs. identical genes coding for surface proteins differs significantly from that in translation-related genes and total genes, following the same trend as the ratio of non-synonymous vs. synonymous substitutions. This is not the case for virulence factors. The percentage of non-identical vs. identical genes reflected differences at the nucleic acids level, while the non-synonymous vs. synonymous differences emphasized the DNA mutations that result in a change protein sequences. Proteins are the ultimate functional entities in an organism, and this makes it reasonable to focus on the DNA mutations that change the coded amino acids, rather than silent mutations.

Of the 931 SNPs orthologous pairs identified in this study, a total of 40 genes showed a disproportionate distribution of dN/dS. Most are probably significant, as we consistently found that when present the imbalanced dN/dS distribution extended to genes common to an operon. That they may play important roles in pathogenic processes is suggested by the presence in the list of genes coding for proteins that are likely to influence pathogenicity, such as phenol-soluble modulin (PSM) family peptides (SE0847) or the Clp protease (SE0674). PSMs, a class of surfactant peptides with proinflammatory and putative biofilm-inhibitory properties, presumably represent key factors controlling the switch between the colonization and disseminative stages of the pathogen. Suppression of production of PSMs in the biofilm stage enables cells to stick together and to evade the host immune defense [31]. ATP-dependent Clp proteases are involved in regulation processes by proteolysis in many bacteria. They consist of a proteolytic subunit, ClpP, which confers substrate specificity through association with ATPase subunits. Signature-tagged mutagenesis screening experiments have demonstrated that ATP-dependent proteases are key factors in bacterial adaptation to environmental stress, including ROS [32]. Little is known about *clp *gene regulation in pathogenic bacteria, despite the fact that many of these genes play important roles in virulence, such as ClpX in *Staphylococcus aureus *[33] and ClpB in *Leishmania *sp. [34]. It seems plausible that these bifunctional chaperones/proteases may also modulate the activity of other virulence factors [35] and it is therefore interesting to have observed that a member of this class is evolving fast in the *S. epidermidis *pair studied here. Genes involved in osmoprotection which may respond to high salt conditions in cell agglomerations on human skin, such as *gbsA *(glycine betaine aldehyde dehydrogenase) were also found among the non-synonymous significant genes.

Surface proteins play a fundamental role in the interaction between the bacterial cell and its environment. In the present case, several of the 40 non-synonymous genes, in particular some genes with unknown function, have features suggesting that they code for surface proteins under the pressure of escaping the host immune system. Interestingly, SE1527 and SE0790 are orthologous to surface proteins experimentally identified in one of Group A *Streptococcus *(GAS) strains (SPY0792 and SPY2018) [36]. It may also be significant that two ribosomal proteins, for which there is also some evidence of extracellular functions, were also found in their surface proteome. This makes RpsL (SE0309) and RplC (SE1824), the two ribosomal proteins which have more non-synonymous substitutions than expected in our analysis more interesting. RpsL, protein S12 of the small subunit of the ribosome is known to control accuracy of translation, and this might indeed play a role in pathogenicity [37]. Further work is required to investigate this aspect as we can expect that some of the genes identified are simply the result of random deviation from the average.

Yao et al have used microaray-based genome-wide comparison of clinical and commensal strains to identify putative virulence factors in *S. epidermidis *[38]. 39 genes were found to be more frequent among clinical strains than commensal strains. Interestingly, we find that after detailed comparison our 40 high-non-synonymous substitutions genes have no overlap with those 39 high-frequent genes in clinical strains. This is probably not unexpected, for the following reasons. Firstly, we looked for conserved genes rather than genes that would differ in a variety of strains, and our study involves only orthologous pairs of genes in the two stains, while Yao et al focused on those genes which showed disproportionate difference in distribution between clinical and commensal strains. Second, our 40 high non-synonymous substitution genes were identified because they carry SNPs, small differences which may not be detected by hybridization with the oligonucleotides of the microarray.

Under laboratory growth conditions, with plenty of metabolites available, bacterial gene expression is typically dominated by the highly expressed genes involved in transcription, protein biosynthesis, maturation and folding. In contrast, as shown for example in a study by Rollenhagen and Bumann, most of the genes highly expressed in *Salmonella enterica *cells recovered from the caecum differ considerably from those highly expressed in bacteria located in the spleen [39]. The overall functional profile of highly expressed genes suggests a marked shift in transcriptional activity upon change in growth environment. In this respect, some pathogenicity related genes are likely to be highly expressed during infection to fight against the host. Interestingly, we found that one of the non-synonymous significant genes of the present study, gene *se2269 *encoding dihydropteroate synthase and recovered as a virulence-related antigen of a Gram-positive fish pathogen [40], was significantly more expressed in RP62A than in ATCC12228 (unpublished data).

Since pathogenicity is a recent form of lifestyle for an organism in evolution, we expected that genes involved in the process would show a significant mutation pattern [41]. Furthermore, we could expect that many of the corresponding mutations would not yet be adapted to the "self" of the bacteria, as they would result from non-synonymous mutations in codons, leading to alteration of the polypeptide sequence of the corresponding genes. Our observations substantiated this hypothesis, showing a significant number of genes, often grouped in operons, affected by non-synonymous mutations. In parallel – and this can be seen as an internal validation of our hypothesis – we observed that genes of the translation machinery, expected to be expressed at a high level (and hence under a "mutator" pressure) were indeed mutated, but that the corresponding mutations were usually of the synonymous class. This is easily accounted for by the enormous selection pressure operating on the translation machinery (made of proteins interacting with each other and with highly conserved RNAs), which cannot easily accommodate mutations resulting in alteration of the corresponding proteins. Even if important for pathogenicity, these genes would become invisible because the functional constraints would result in a purifying selection process. However not all genes can be equally submitted to purifying selection and our approach reveals several genes of the general cell machinery, as possibly important for pathogenicity. As a case in point, we found one gene (*se1042*) coding for peptide methionine sulfoxide reductase, involved in protection against ROS, which could probably be such an important candidate for adaptation to pathogenicity [24,25]. As the *clpB *gene found in our non-synonymous set also may point to adaptation to ROS, we explored the differential resistance to H_2_O_2 _and paraquat of the environmental species as compared to the pathogen (Figure 2 and 3, see Additional file 6 and file 7) and our experiments substantiated the interest of our approach to identify specific pathways of evolution to pathogenicity. In the same way a (presumably divalent) cation transporter SE0773 might be involved in the scavenging of divalent metals such as magnesium, manganese, iron or cobalt during infection. Gene *se2128 *might code for polyphosphate-AMP phosphotransferase, an observation that would support interest for the poorly explored role of polyphosphate in cells and its possible involvement in pathogenicity [42]. Others are involved in biofilm formation, an important contribution to virulence in several pathogenic bacteria [43], as they considerably limit the success of both antibiotic treatment and the human immune defense. Gene expression profiling of the *S. epidermidis *biofilm was analyzed and some significant metabolic shift was found between the planktonic and the biofilm modes of growth [31].

## Conclusion

In this paper, a SNPs comparative approach was developed to identify conserved genes possibly involved in pathogenicity by measuring their selective pressure in the gene pairs of non-infection associated *S. epidermidis *strain ATCC12228 and biofilm-forming strain RP62A. Our approach identified new genes that may be involved in pathogenesis, including some genes with unknown function. These results may provide fresh insight into discovering the genes that determine the success of *S. epidermidis *as an opportunistic pathogen. Complementing the previous methodologies which mainly focused on horizontal gene transfer, extensive SNPs investigations on more pathogens will facilitate our understanding of the path from commensalism to pathogenicity, a crucial prerequisite for designing therapeutic interventions directed to control pathogen infections.

## Methods

### Orthologous genes identification

For the identification of orthologous genes, all predicted CDSs from the *S. epidermidis *RP62A and ATCC12228 genomes were searched against each other locally using BLASTP [44]. Those genes that matched a non-self genomic sequence at *P *value of < = 10^-5^, and identity > = 35%, matching at least 75% of the length of both query and subject sequences were considered homologous (non strain-specific genes). Of these homologous pairs, the bidirectional-best match was defined as an orthologous group. Identical nucleic acid sequence pairs, SNPs and insertion/deletions of the genes' pairs of orthologs between the two *S. epidermidis *genomes were identified using BLASTN.

### SNPs analysis

The Nei-Gojobori (NG) method [45] was applied for estimating dS and dN of the SNPs pairs of *S. epidermidis *strains with minor modifications. The ratio of transition to transversion changes (R) was obtained by counting the total numbers of transitions to transversions that were observed in the entire set of orthologous pairs of the two species and adjusted using Kimura's 2-parameters method [46]. The adjusted R computed from our data was 2.330294 and used in the following computation. We also applied the one-tail Z (or *t*∞) test to conduct the statistical test of the positiveness of dN – dS. The estimated dS and dN, standard error (S.E.), Z-scores and their corresponding p-values were presented in the additional file 5. To explore the distribution of dN/dS, we applied the transformation *f *(*k*) = log(*k *+ 0.001) as described in other works[20,47].

### H_2_O_2 _and paraquat sensitivity analysis

We grew the S. *epidermidis *strains at different concentration of H_2_O_2 _and measured the growth curve of the strains by reading OD_595 _every one hour. Overnight bacterial cultures at (OD_595 _approximately 1.2) were added to fresh TSB(Tryptone Soya Broth) at a 1:100 dilution, and different concentrations of H_2_O_2 _(0, 1%, 5%, 10% and 15%) were added at the same time. Cells were grown at 37°C, agitated at, 220 rpm and the absorbancy of the culture (OD_595_) was followed every one hour.

MIC (minimal inhibitory concentration) assay of paraquat (sigma) by broth dilution was performed according to NCCLS (National Committee for Clinical Laboratory Standards) [48]. The bacterial inoculum was prepared using a 3–4 h broth culture of each isolates adjusted to a turbidity equivalent to a 0.5 McFarland standard, diluted in CAMHB to achieve a final concentration of 5 × 10^5 ^CFU/ml in the test tube. Broth not containing an antimicrobial agent is inoculated as a control for organism viability, and *E.coli *ATCC 25922 was used as a test quality control strain. The bacteria were cultured in MH broth (OXOID) with different concentration of paraquat (from 1 mM to 8 mM) and incubated at 35°C for 16–20 h. The MIC was defined as the lowest concentration of paraquat giving complete inhibition of visible growth. The growth of bacteria incubated with lower concentration paraquat than MIC was measured by OD_600_.

### PCR validation

In order to get PCR fragments containing SNPs, we designed primers by Primer Premier 5.0. The primers sequences are listed in Additional file 8. We used Pfu DNA polymerase (Tiangen Biotechnology Co., Ltd) to ensure high-fidelity synthesis. Amplifications were carried out in a thermocycler (GeneAmp PCR system 9700) through the following temperature program: 1 cycle of 5 min at 94°C; 30 cycles of 30 s at 94°C, 60 s at 55°C, and 60 s at 72°C; and finally 1 cycle at 72°C for 7 min. The PCR products were purified by agarose gel DNA purification kit (TaKaRa Biotechnology Co., Ltd) and sequenced by Shanghai Invitrogen Biotechnology Co., Ltd.

## Abbreviations

dN, number of non-synonymous substitutions per site; dS, number of synonymous substitutions per site; CDS, Coding DNA sequence; SNPs, Single-nucleotide polymorphisms.

## Authors' contributions

WW performed the genome comparative analysis of the two strains and drafted the manuscript. ZWC contributed to conceive the study and draft the manuscript. YLZ contributed to H_2_O_2 _and paraquat sensitivity analysis and PCR experiments. XJW contributed to comparative analysis of the CDSs of both strains. GHD contributed to the statistical analysis of SNPs pairs. HX contributed to the identification of orthologs and PCR primer design. PLJ contributed to sequences analysis of PCR results. DQ contributed to conceive the study. AD conceived the rationale for the mutation analysis study and contributed to the writing of the manuscript. YXL contributed to conceive the study and revised the manuscript. All authors read and approved the final manuscript.

## Supplementary Material

Additional File 1 Distribution of insertions in either of the two sequenced *Staphylococcus epidermidis *genomes. Click here for file

Additional File 2 Distribution of orthologs of two *Staphylococcus epidermidis *strains. Click here for file

Additional File 3 Ratios of non-synonymous vs synonymous of orthologs with SNPs pairs of Virulence factors. Click here for file

Additional File 4 Ratios of non-synonymous vs synonymous of orthologs with SNPs pairs of Surface proteins. Click here for file

Additional File 5 Ratios of non-synonymous vs synonymous of orthologs with all SNPs pairs. Click here for file

Additional File 6 **Comparison the sensitivity to H_2_O_2 _of both *Staphylococcus epidermidis *strains**. Each bar represents the OD_595 _value of one strain at specific time and concentration of H_2_O_2_. Click here for file

Additional File 7 **Comparison the sensitivity to H_2_O_2 _(5%) of both *Staphylococcus epidermidis *strains**. Red bars represent the OD_595 _value of *S. epidermidis *RP62A at different time and blue bars represent *S. epidermidis *ATCC12228. Click here for file

Additional File 9 Comparison of identical and non-identical distribution of different functional groups of orthologous genes. Click here for file

Additional File 8 Primers used in the PCR experiments. Click here for file

## Figures and Tables

**Table 1:** Comparison of the CDSs of *S. epidermidis *ATCC 12228 and *S. epidermidis *RP62A

	*S. epidermidis *ATCC12228^a^	*S. epidermidis *RP62A^a^
Total CDS number	2419	2494
CDSs categorized by sequence variations		
All homologs	2094	2103
Total orthologs	2053	2053
Orthologs with identical DNA sequences	859	859
Orthologs with SNPs and the same length^b^	931	931
Orthologs with insertions/deletions	263	263
Paralogs	41	50
Unique compared to the other *S. epidermidis*^c^	325	391
Unique compared to all staphylococcal	288	315

^a ^Genomic data of *S. epidermidis *ATCC12228 (accession no. AE015934) and *S. epidermidis *RP62A (accession no. CP000029) are according to GeneBank.

^b ^These CDSs were used in analyzing the variation of single-nucleotide polymorphisms (SNPs) between two *S. epidermidis *genome.

^c ^These CDSs were specific to each other.

**Table 2:** Comparison of SNP frequency and distribution into different functional groups

Groups^a^	No.CDS	n+s	n^b^	s	n/s	*P*_I_^c^	*P*_II_	*P*_III_
I	11	112	47	65	0.723			
II	6	84	34	50	0.68	0.834		
III	48	400	99	301	0.329	0.000*	0.003*	
Total	931	7322	2151	5171	0.416	0.003* ^d^	0.025* ^d^	0.047* ^d^

^a^Groups defined as (I) genes whose products are virulence factors and genomic islands in both *S. epidermidis *genome; (II) genes whose products are surface proteins; (III) genes whose products belong to translation, ribosomal structure and biogenesis catalog according to COG.

^b^(n) number of non-synonymous SNPs; (s) number of synonymous SNPs; (n/s) ratios of non-synonymous SNPs vs. synonymous SNPs.

^c ^*P*-value from χ^2 ^test for the difference in n/s ratios in all the pairwise comparisons among the three functional groups (* indicates *p *< 0.05).

^d^*P*-value from χ^2 ^test for the difference in n/s ratios between the three functional groups and total without themselves (* indicates *p *< 0.05).

**Table 3:** Non-synonymous substitutions significant group (p < 0.10) of Orthologs with SNP Pairs potential involves in pathogenicity

*S. epidermidis *ATCC12228	*S. epidermidis *RP62A	Function		N	S	dN/(dS+ dN)	p value
						
		Annotation from ATCC12228	New annotation				
SE1029	SERP0918	exonuclease SbcC		5	0	1.0000	0.0107
SE0231	SERP2349	fosfomycin resistance protein fofB		4	0	1.0000	0.0228
SE0800	SERP0689	potD protein		3	0	1.0000	0.0381
SE0265	SERP2313	hypothetical protein	Tranmembrane protein	3	0	1.0000	0.0383
SE1546	SERP1399	D-3-phosphoglycerate dehydrogenase		3	0	1.0000	0.0392
SE0378	SERP0259	hypothetical protein	Tranmembrane protein	3	0	1.0000	0.0398
SE2085	SERP2099	gluconokinase		3	0	1.0000	0.0401
SE1042	SERP0931	peptide methionine sulfoxide reductase		3	0	1.0000	0.0405
SE0039	SERP2495	conserved hypothetical protein		3	0	1.0000	0.0406
SE0977	SERP0866	glycerol uptake facilitator		3	0	1.0000	0.0415
SE1170	SERP1049	probable ATP-dependent DNA helicase RecQ		3	0	1.0000	0.0425
SE0502	SERP0385	choline transport ATP-binding protein		3	0	1.0000	0.0427
SE0546	SERP0431	conserved hypothetical protein	YvcD	3	0	1.0000	0.0440
SE1302	SERP1183	deoxyribonuclease		2	0	1.0000	0.0668
SE1527	SERP1382	conserved hypothetical protein	YheB (surface protein)	2	0	1.0000	0.0707
SE2166	SERP2177	glycine betaine aldehyde dehydrogenase gbsA		2	0	1.0000	0.0719
SE2379	SERP0037	cystathionine gamma-synthase		2	0	1.0000	0.0743
SE1828	SERP1836	DNA topoisomerase III topB		2	0	1.0000	0.0743
SE1824	SERP1831	50S ribosomal protein L3		2	0	1.0000	0.0759
SE0309	SERP0186	30S ribosomal protein S12		2	0	1.0000	0.0766
SE0463	SERP0349	putative deoxyribodipyrimidine photolyase		2	0	1.0000	0.0766
SE2067	SERP2080	glycine betaine aldehyde dehydrogenase gbsA		2	0	1.0000	0.0766
SE2215	SERP2247	arginine/oirnithine antiporter		2	0	1.0000	0.0766
SE0806	SERP0695	conserved hypothetical protein	YlaF	2	0	1.0000	0.0768
SE1448	SERP1335	conserved hypothetical protein	YqgE (MultiDrug Efflux proteins)	2	0	1.0000	0.0775
SE0847	SERP0737	hypothetical protein	phenol soluble modulin beta 1	2	0	1.0000	0.0777
SE0790	SERP0679	conserved hypothetical protein	YkyA (surface protein)	2	0	1.0000	0.0779
SE2196	SERP2207	conserved hypothetical protein		2	0	1.0000	0.0781
SE2269	SERP0153	dihydropteroate synthase chain A synthetase		2	0	1.0000	0.0783
SE0744	SERP0630	2-oxoglutarate decarboxylase		2	0	1.0000	0.0783
SE2003	SERP2016	conserved hypothetical protein		2	0	1.0000	0.0783
SE1247	SERP1126	conserved hypothetical protein	YqfN	2	0	1.0000	0.0787
SE1986	SERP1998	conserved hypothetical protein	In larger Bacterial clades	2	0	1.0000	0.0800
SE0432	SERP0317	low-affinity inorganic phosphate transporter		2	0	1.0000	0.0808
SE1745	SERP1754	conserved hypothetical protein	YhfK	2	0	1.0000	0.0827
SE0674	SERP0564	clpB protein		2	0	1.0000	0.0846
SE0773	SERP0660	conserved hypothetical protein	Cation transporter	2	0	1.0000	0.0846
SE1608	SERP1461	beta-lactamase		2	0	1.0000	0.0846
SE2128	SERP2140	conserved hypothetical protein	YfmM	2	0	1.0000	0.0874
SE0506	SERP0389	alcohol dehydrogenase		7	1	0.7451	0.0953
