# Supplementary material for: Conserved genes in a path from commensalism to pathogenicity: comparative phylogenetic profiles of Staphylococcus epidermidis RP62A and ATCC12228
Source: BMC Genomics. 2006 May 10;7:112. doi: 10.1186/1471-2164-7-112 (PMC1482698; doi:10.1186/1471-2164-7-112)
Supplement: Additional File 1 — Distribution of insertions in either of the two sequenced Staphylococcus epidermidis genomes. [file 1471-2164-7-112-S1.pdf]

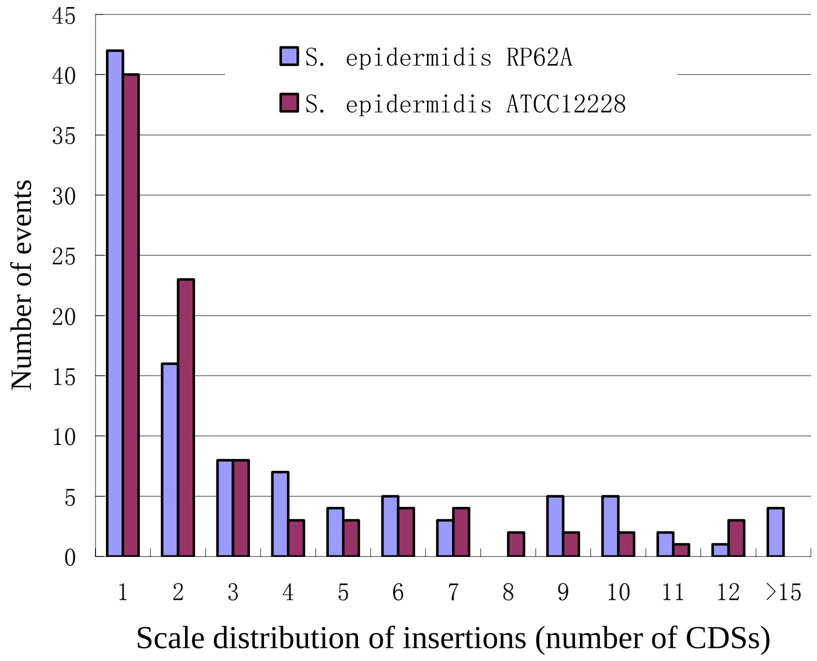

Additional file 1. Distribution of insertions in either of the two sequenced *Staphylococcus epidermidis* genomes.
