# Supplementary figures and images for: Conserved genes in a path from commensalism to pathogenicity: comparative phylogenetic profiles of Staphylococcus epidermidis RP62A and ATCC12228
Source: BMC Genomics. 2006 May 10;7:112. doi: 10.1186/1471-2164-7-112 (PMC1482698; doi:10.1186/1471-2164-7-112)

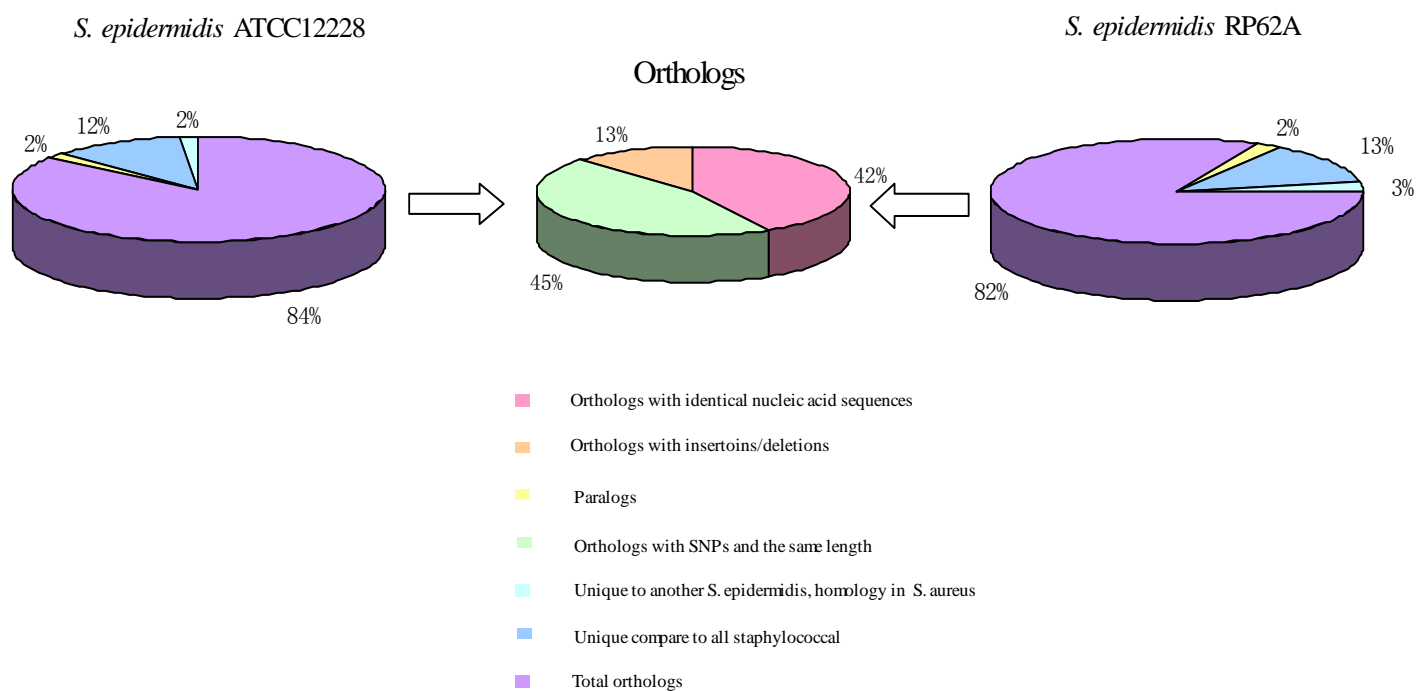

**Additional file 2.** Distribution of orthologs of two *Staphylococcus epidermidis* strains.

Supplement: Additional File 2 — Distribution of orthologs of two Staphylococcus epidermidis strains. [file 1471-2164-7-112-S2.pdf]
