# Supplementary material for: Conserved genes in a path from commensalism to pathogenicity: comparative phylogenetic profiles of Staphylococcus epidermidis RP62A and ATCC12228
Source: BMC Genomics. 2006 May 10;7:112. doi: 10.1186/1471-2164-7-112 (PMC1482698; doi:10.1186/1471-2164-7-112)
Supplement: Additional File 3 — Ratios of non-synonymous vs synonymous of orthologs with SNPs pairs of Virulence factors. [file 1471-2164-7-112-S3.pdf]

Additional file 3. Ratios of nonsynonymous vs synonymous of orthologs with SNPs pairs of Virulence factors.

Virulence factor	*S. epidermidis* RP62A	name	function	Compare to ATCC12228	*S. epidermidis* ATCC12228	n	s	dn	ds	dn/ds	*p* value
Esterase	SERP1941	-	esterase, putative	identical	SE1929						
	SERP2109	-	esterase, putative	SNP	SE2095	1	4	0.0014	0.0144	0.0886	0.9618
Serine protease	SERP1292	-	serine protease HtrA, putative	SNP	SE1405	2	1	0.0023	0.0028	0.4510	0.5616
	SERP2401	-	serine protease	specific							
Serine V8 protease	SERP1397	sspA	glutamyl endopeptidase precursor SspA	SNP	SE1543	1	0	0.0016	0.0000	1.0000	0.1587
Cysteine protease	SERP2390	sspB	cysteine protease precursor SspB	identical	SE0184						
	SERP2391	sspC	SspC protein	indel	SE0183						
Lipase	SERP2336	-	lipase, putative	SNP	SE0245	6	20	0.0038	0.0336	0.1016	0.9999
	SERP0018	-	lipase, putative	SNP	SE2403	1	0	0.0007	0.0000	1.0000	0.1587
	SERP2297	geh-1	lipase	SNP	SE0281	15	21	0.0102	0.0369	0.2166	0.9992
	SERP2388	geh-2	lipase	SNP	SE0185	16.5	16.5	0.0120	0.0310	0.2791	0.9900
Lipase/esterase	SERP0309	-	lipase/esterase, putative	identical	SE0424						
	SERP2252	sepA	extracellular elastase precursor	SNP	SE2219	1	1	0.0009	0.0023	0.2813	0.7146
Beta hemolysin	SERP2544	hlb	beta-hemolysin	identical	SE0008						
Delta hemolysin	SERP1489	hld	delta-hemolysin	identical	SE1634						
Hemolysin III	SERP1769	-	hemolysin III, putative	identical	SE1760						
Hemolysin	SERP2258	-	hemolysin, putative	indel	SE2226						
Thermonuclease	SERP0891	-	thermonuclease precursor family protein	indel	SE1004						
nuclease	SERP1570	-	Staphylococcal nuclease family protein	specific							
Zinc metalloprotense	SERP0829	-	membrane-associated zinc metalloprotease, putative	identical	SE0938						
Clp protease, procolytic subunit	SERP0436	clpP	ATP-dependent Clp protease, proteolytic subunit ClpP	identical	SE0551						
Clp protease, ATP binding subunit	SERP0564	clpB	ATP-dependent Clp protease, ATP-binding subunit ClpB	SNP	SE0674	2	0	0.0011	0.0000	1.0000	0.0846
Clp protease, ATP binding subunit	SERP1238	clpX	ATP-dependent Clp protease, ATP-binding subunit ClpX	identical	SE1349						
Clp protease, ATP binding subunit	SERP0165	clpC	ATP-dependent Clp protease, ATP-binding subunit ClpC	identical	SE0287						

Phenol-soluble modulin	SERP0736	-	phenol soluble modulin beta 1	indel	SE0846						
	SERP0737	-	phenol soluble modulin beta 1	SNP	SE0847	2	0	0.0216	0.0000	1.0000	0.0777
	SERP0738	-	phenol soluble modulin beta 1	paralog							
	SERP0739	-	phenol soluble modulin beta 1	SNP	SE0849	0	2	0.0000	0.0553	0.0000	0.9161
	SERP2397	-	phenol soluble modulin beta 1	identical	SE0177						
	SERP2400	-	phenol soluble modulin beta 1	identical	SE0174						
	SERP0083	-	phenol soluble modulin alpha	specific							
	SERP1489	hld	delta-hemolysin	identical	SE1634						
