# Supplementary material for: Conserved genes in a path from commensalism to pathogenicity: comparative phylogenetic profiles of Staphylococcus epidermidis RP62A and ATCC12228
Source: BMC Genomics. 2006 May 10;7:112. doi: 10.1186/1471-2164-7-112 (PMC1482698; doi:10.1186/1471-2164-7-112)
Supplement: Additional File 4 — Ratios of non-synonymous vs synonymous of orthologs with SNPs pairs of Surface proteins. [file 1471-2164-7-112-S4.pdf]

Additional file 4. Ratios of nonsynonymous vs synonymous of orthologs with SNPs pairs of Surface proteins.

S. epidermidis RP62A	name	function	Compare to ATCC12228	*S. epidermidis* ATCC12228	n	s	dn	ds	dn/ds	*p* value
SERP0026	-	sdrF protein, truncation	specific							
SERP0207	-	sdrG protein	indel	SE0331						
SERP1487	sdrH	sdrH protein	indel	SE1632						
SERP1316	-	cell wall surface anchor family protein	SNP	SE1429	14	4	0.0018	0.0013	0.5806	0.2610
SERP2162	-	cell wall surface anchor family protein	SNP	SE2152	2	2	0.0048	0.0117	0.2909	0.7791
SERP2264	-	cell wall surface anchor family protein	SNP	SE2232	7	7	0.0048	0.0123	0.2807	0.9274
SERP2392	bhp	cell wall associated biofilm protein	paralog							
SERP0719	-	cell wall surface anchor family protein	indel	SE0828						
SERP2398	aap	accumulation associated protein	indel	SE0175						
SERP1482	-	cell wall surface anchor family protein	specific							
SERP1483	-	cell wall surface anchor family protein	SNP	SE1628	6	4	0.0078	0.0135	0.3662	0.7759
SERP1654	-	cell wall surface anchor family protein	specific							
		hypothetical protein	specific	SE1500						
		hypothetical protein	specific	SE1501						
SERP1011	ebh	cell wall associated fibronectin-binding protein	indel	SE1128						
SERP1048	-	elastin binding protein, putative	indel	SE1169						
SERP0636	atlE	bifunctional autolysin	SNP	SE0750	1	1	0.0004	0.0009	0.3077	0.6942
SERP0775	fbe	fibronectin/fibrinogen binding protein	SNP	SE0884	4	32	0.0033	0.0709	0.0445	1.0000
