# Supplementary material for: Conserved genes in a path from commensalism to pathogenicity: comparative phylogenetic profiles of Staphylococcus epidermidis RP62A and ATCC12228
Source: BMC Genomics. 2006 May 10;7:112. doi: 10.1186/1471-2164-7-112 (PMC1482698; doi:10.1186/1471-2164-7-112)
Supplement: Additional File 5 — Ratios of non-synonymous vs synonymous of orthologs with all SNPs pairs. [file 1471-2164-7-112-S5.pdf]

Additional file 5. Ratios of nonsynonymous vs synonymous of orthologs with all SNPs pairs.

orthlogs with SNPs and same length		R	*S. epidermidis* RP62A		*S. epidermidis* ATCC12228		AVERAGE		*n*	*s*	p_n	p_s	d_n	d_s	d_n/d_s	$d_n/(d_s+d_n)$	Z score*	p value‡
			N	S	N	S	N	S										
SE1029	SERP0918	2.330294	2188.224	838.776	2187.701	839.299	2189.051	837.949	5	0	0.0023	0.0000	0.0023	0.0000	–	1.0000	2.3000	0.0107
SE0231	SERP2349	2.330294	310.396	115.604	311.246	114.754	311.909	114.091	4	0	0.0129	0.0000	0.0130	0.0000	–	1.0000	2.0000	0.0228
SE0800	SERP0689	2.330294	771.377	299.623	771.377	299.623	772.466	298.534	3	0	0.0039	0.0000	0.0039	0.0000	–	1.0000	1.7727	0.0381
SE0265	SERP2313	2.330294	74.756	27.244	75.880	26.120	76.406	25.594	3	0	0.0398	0.0000	0.0409	0.0000	–	1.0000	1.7706	0.0383
SE1546	SERP1399	2.330294	681.315	266.685	681.014	266.986	682.253	265.747	3	0	0.0044	0.0000	0.0044	0.0000	–	1.0000	1.7600	0.0392
SE0378	SERP0259	2.330294	79.979	28.021	80.129	27.871	81.142	26.858	3	0	0.0375	0.0000	0.0384	0.0000	–	1.0000	1.7534	0.0398
SE2085	SERP2099	2.330294	1062.091	425.909	1061.967	426.033	1063.117	424.883	3	0	0.0028	0.0000	0.0028	0.0000	–	1.0000	1.7500	0.0401
SE1042	SERP0931	2.330294	369.102	143.898	369.102	143.898	370.190	142.810	3	0	0.0081	0.0000	0.0082	0.0000	–	1.0000	1.7447	0.0405
SE0039	SERP2495	2.330294	210.692	83.308	211.469	82.531	212.168	81.832	3	0	0.0142	0.0000	0.0143	0.0000	–	1.0000	1.7439	0.0406
SE0977	SERP0866	2.330294	581.185	240.815	580.185	241.815	581.773	240.227	3	0	0.0052	0.0000	0.0052	0.0000	–	1.0000	1.7333	0.0415
SE1170	SERP1049	2.330294	979.954	397.046	978.954	398.046	980.543	396.457	3	0	0.0031	0.0000	0.0031	0.0000	–	1.0000	1.7222	0.0425
SE0502	SERP0385	2.330294	692.875	279.125	692.274	279.726	693.663	278.337	3	0	0.0043	0.0000	0.0043	0.0000	–	1.0000	1.7200	0.0427
SE0546	SERP0431	2.330294	1028.104	408.896	1028.104	408.896	1029.192	407.808	3	0	0.0029	0.0000	0.0029	0.0000	–	1.0000	1.7059	0.0440
SE1302	SERP1183	2.330294	1737.146	692.854	1736.995	693.005	1738.159	691.841	2	0	0.0012	0.0000	0.0012	0.0000	–	1.0000	1.5000	0.0668
SE1527	SERP1382	2.330294	808.136	319.864	810.235	317.765	810.273	317.727	2	0	0.0025	0.0000	0.0025	0.0000	–	1.0000	1.4706	0.0707
SE2166	SERP2177	2.330294	1062.813	425.187	1062.663	425.337	1063.826	424.174	2	0	0.0019	0.0000	0.0019	0.0000	–	1.0000	1.4615	0.0719
SE2379	SERP0037	2.330294	776.000	325.000	776.000	325.000	777.089	323.911	2	0	0.0026	0.0000	0.0026	0.0000	–	1.0000	1.4444	0.0743
SE1828	SERP1836	2.330294	1527.764	605.236	1527.764	605.236	1528.853	604.147	2	0	0.0013	0.0000	0.0013	0.0000	–	1.0000	1.4444	0.0743
SE1824	SERP1831	2.330294	472.175	187.825	471.476	188.524	472.914	187.086	2	0	0.0042	0.0000	0.0043	0.0000	–	1.0000	1.4333	0.0759
SE0309	SERP0186	2.330294	287.415	123.585	287.239	123.761	288.415	122.585	2	0	0.0070	0.0000	0.0070	0.0000	–	1.0000	1.4286	0.0766
SE0463	SERP0349	2.330294	1003.008	367.992	1002.407	368.593	1003.796	367.204	2	0	0.0020	0.0000	0.0020	0.0000	–	1.0000	1.4286	0.0766
SE2067	SERP2080	2.330294	1023.615	413.385	1023.615	413.385	1024.703	412.297	2	0	0.0020	0.0000	0.0020	0.0000	–	1.0000	1.4286	0.0766
SE2215	SERP2247	2.330294	994.808	406.192	994.658	406.342	995.821	405.179	2	0	0.0020	0.0000	0.0020	0.0000	–	1.0000	1.4286	0.0766
SE0806	SERP0695	2.330294	137.609	51.391	137.759	51.241	138.772	50.228	2	0	0.0145	0.0000	0.0147	0.0000	–	1.0000	1.4272	0.0768
SE1448	SERP1335	2.330294	315.899	125.101	315.599	125.401	316.837	124.163	2	0	0.0063	0.0000	0.0064	0.0000	–	1.0000	1.4222	0.0775
SE0847	SERP0737	2.330294	93.931	38.069	93.931	38.069	95.019	36.981	2	0	0.0213	0.0000	0.0216	0.0000	–	1.0000	1.4211	0.0777
SE0790	SERP0679	2.330294	454.862	169.138	455.162	168.838	456.100	167.900	2	0	0.0044	0.0000	0.0044	0.0000	–	1.0000	1.4194	0.0779
SE2196	SERP2207	2.330294	129.585	47.415	130.035	46.965	130.898	46.102	2	0	0.0154	0.0000	0.0156	0.0000	–	1.0000	1.4182	0.0781
SE2269	SERP0153	2.330294	585.303	230.697	584.303	231.697	585.891	230.109	2	0	0.0034	0.0000	0.0034	0.0000	–	1.0000	1.4167	0.0783
SE0744	SERP0630	2.330294	1181.679	489.321	1182.378	488.622	1183.117	487.883	2	0	0.0017	0.0000	0.0017	0.0000	–	1.0000	1.4167	0.0783
SE2003	SERP2016	2.330294	584.037	222.963	584.037	222.963	585.125	221.875	2	0	0.0034	0.0000	0.0034	0.0000	–	1.0000	1.4167	0.0783
SE1247	SERP1126	2.330294	491.554	195.446	491.554	195.446	492.643	194.357	2	0	0.0041	0.0000	0.0041	0.0000	–	1.0000	1.4138	0.0787

SE1986	SERP1998	2.330294	337.494	130.506	338.194	129.806	338.932	129.068	2	0	0.0059	0.0000	0.0059	0.0000	–	1.0000	1.4048	0.0800
SE0432	SERP0317	2.330294	707.563	300.437	707.563	300.437	708.651	299.349	2	0	0.0028	0.0000	0.0028	0.0000	–	1.0000	1.4000	0.0808
SE1745	SERP1754	2.330294	463.830	190.170	462.980	191.020	464.493	189.507	2	0	0.0043	0.0000	0.0043	0.0000	–	1.0000	1.3871	0.0827
SE0674	SERP0564	2.330294	1860.768	746.232	1860.768	746.232	1861.856	745.144	2	0	0.0011	0.0000	0.0011	0.0000	–	1.0000	1.3750	0.0846
SE0773	SERP0660	2.330294	601.031	235.969	601.332	235.668	602.270	234.730	2	0	0.0033	0.0000	0.0033	0.0000	–	1.0000	1.3750	0.0846
SE1608	SERP1461	2.330294	601.546	241.454	601.696	241.304	602.709	240.291	2	0	0.0033	0.0000	0.0033	0.0000	–	1.0000	1.3750	0.0846
SE2128	SERP2140	2.330294	1031.666	390.334	1032.366	389.634	1033.104	388.896	2	0	0.0019	0.0000	0.0019	0.0000	–	1.0000	1.3571	0.0874
SE0506	SERP0389	2.330294	1859.618	747.382	1858.919	748.081	1860.357	746.643	7	1	0.0038	0.0013	0.0038	0.0013	2.9231	0.7451	1.3086	0.0953
SE1371	SERP1258	2.330294	800.387	318.613	800.687	318.313	801.625	317.375	1	0	0.0012	0.0000	0.0013	0.0000	–	1.0000	1.0833	0.1393
SE1926	SERP1938	2.330294	690.396	254.604	689.997	255.003	691.285	253.715	1	0	0.0014	0.0000	0.0015	0.0000	–	1.0000	1.0714	0.1420
SE1792	SERP1800	2.330294	571.242	229.758	571.942	229.058	572.680	228.320	1	0	0.0017	0.0000	0.0018	0.0000	–	1.0000	1.0588	0.1448
SE1195	SERP1075	2.330294	308.350	126.650	308.350	126.650	309.438	125.562	1	0	0.0032	0.0000	0.0033	0.0000	–	1.0000	1.0313	0.1512
SE1256	SERP1136	2.330294	253.319	100.681	253.319	100.681	254.407	99.593	1	0	0.0039	0.0000	0.0040	0.0000	–	1.0000	1.0256	0.1525
SE0417	SERP0302	2.330294	100.885	46.115	100.584	46.416	101.823	45.177	1	0	0.0099	0.0000	0.0100	0.0000	–	1.0000	1.0101	0.1562
SE0246	SERP2335	2.330294	68.005	24.995	68.306	24.694	69.244	23.756	1	0	0.0147	0.0000	0.0148	0.0000	–	1.0000	1.0068	0.1570
SE2364	SERP0052	2.330294	421.127	166.873	421.604	166.396	422.454	165.546	6	1	0.0142	0.0060	0.0144	0.0060	2.4000	0.7059	1.0066	0.1571
SE1189	SERP1068	2.330294	65.756	27.244	66.456	26.544	67.194	25.806	1	0	0.0151	0.0000	0.0153	0.0000	–	1.0000	1.0066	0.1571
SE2403	SERP0018	2.330294	1468.535	574.465	1468.535	574.465	1469.623	573.377	1	0	0.0007	0.0000	0.0007	0.0000	–	1.0000	1.0000	0.1587
SE2402	SERP0019	2.330294	134.384	54.616	134.234	54.766	135.397	53.603	1	0	0.0074	0.0000	0.0075	0.0000	–	1.0000	1.0000	0.1587
SE2383	SERP0033	2.330294	529.932	211.068	529.932	211.068	531.020	209.980	1	0	0.0019	0.0000	0.0019	0.0000	–	1.0000	1.0000	0.1587
SE2382	SERP0034	2.330294	1584.792	659.208	1584.642	659.358	1585.805	658.195	1	0	0.0006	0.0000	0.0006	0.0000	–	1.0000	1.0000	0.1587
SE2360	SERP0057	2.330294	449.738	171.262	449.738	171.262	450.826	170.174	1	0	0.0022	0.0000	0.0022	0.0000	–	1.0000	1.0000	0.1587
SE2357	SERP0060	2.330294	402.809	164.191	402.809	164.191	403.898	163.102	1	0	0.0025	0.0000	0.0025	0.0000	–	1.0000	1.0000	0.1587
SE2352	SERP0065	2.330294	474.857	188.143	474.857	188.143	475.946	187.054	1	0	0.0021	0.0000	0.0021	0.0000	–	1.0000	1.0000	0.1587
SE2335	SERP0080	2.330294	851.217	348.783	851.217	348.783	852.305	347.695	1	0	0.0012	0.0000	0.0012	0.0000	–	1.0000	1.0000	0.1587
SE2333	SERP0084	2.330294	1058.384	435.616	1058.684	435.316	1059.623	434.377	1	0	0.0009	0.0000	0.0009	0.0000	–	1.0000	1.0000	0.1587
SE2321	SERP0098	2.330294	454.944	202.056	455.244	201.756	456.182	200.818	1	0	0.0022	0.0000	0.0022	0.0000	–	1.0000	1.0000	0.1587
SE2302	SERP0119	2.330294	962.835	372.165	963.136	371.864	964.074	370.926	1	0	0.0010	0.0000	0.0010	0.0000	–	1.0000	1.0000	0.1587
SE2294	SERP0127	2.330294	594.538	242.462	594.538	242.462	595.626	241.374	1	0	0.0017	0.0000	0.0017	0.0000	–	1.0000	1.0000	0.1587
SE2282	SERP0139	2.330294	467.156	189.844	467.457	189.543	468.395	188.605	1	0	0.0021	0.0000	0.0021	0.0000	–	1.0000	1.0000	0.1587
SE2280	SERP0141	2.330294	2498.416	1008.584	2498.416	1008.584	2499.504	1007.496	1	0	0.0004	0.0000	0.0004	0.0000	–	1.0000	1.0000	0.1587
SE0397	SERP0281	2.330294	1689.249	710.751	1689.549	710.451	1690.487	709.513	1	0	0.0006	0.0000	0.0006	0.0000	–	1.0000	1.0000	0.1587
SE0409	SERP0294	2.330294	521.554	222.446	520.854	223.146	522.292	221.708	1	0	0.0019	0.0000	0.0019	0.0000	–	1.0000	1.0000	0.1587
SE0418	SERP0303	2.330294	1224.502	500.498	1224.202	500.798	1225.441	499.559	1	0	0.0008	0.0000	0.0008	0.0000	–	1.0000	1.0000	0.1587
SE0451	SERP0336	2.330294	320.888	126.112	320.888	126.112	321.976	125.024	1	0	0.0031	0.0000	0.0031	0.0000	–	1.0000	1.0000	0.1587
SE0526	SERP0411	2.330294	349.465	142.535	349.465	142.535	350.553	141.447	1	0	0.0029	0.0000	0.0029	0.0000	–	1.0000	1.0000	0.1587
SE0533	SERP0418	2.330294	483.096	188.904	483.397	188.603	484.335	187.665	1	0	0.0021	0.0000	0.0021	0.0000	–	1.0000	1.0000	0.1587
SE0538	SERP0423	2.330294	461.228	177.772	461.228	177.772	462.317	176.683	1	0	0.0022	0.0000	0.0022	0.0000	–	1.0000	1.0000	0.1587
SE0576	SERP0463	2.330294	361.268	139.732	361.268	139.732	362.356	138.644	1	0	0.0028	0.0000	0.0028	0.0000	–	1.0000	1.0000	0.1587

SE0585	SERP0474	2.330294	142.611	58.389	142.611	58.389	143.700	57.300	1	0	0.0070	0.0000	0.0070	0.0000	–	1.0000	1.0000	0.1587
SE0596	SERP0485	2.330294	271.271	106.729	271.121	106.879	272.284	105.716	1	0	0.0037	0.0000	0.0037	0.0000	–	1.0000	1.0000	0.1587
SE0597	SERP0486	2.330294	64.908	25.092	63.908	26.092	65.497	24.503	1	0	0.0155	0.0000	0.0157	0.0000	–	1.0000	1.0000	0.1587
SE0608	SERP0498	2.330294	886.178	352.822	886.178	352.822	887.266	351.734	1	0	0.0011	0.0000	0.0011	0.0000	–	1.0000	1.0000	0.1587
SE0643	SERP0535	2.330294	1040.061	453.939	1039.761	454.239	1040.999	453.001	1	0	0.0010	0.0000	0.0010	0.0000	–	1.0000	1.0000	0.1587
SE0650	SERP0542	2.330294	803.792	321.208	803.616	321.384	804.792	320.208	1	0	0.0012	0.0000	0.0012	0.0000	–	1.0000	1.0000	0.1587
SE0680	SERP0570	2.330294	647.337	276.663	647.337	276.663	648.425	275.575	1	0	0.0015	0.0000	0.0015	0.0000	–	1.0000	1.0000	0.1587
SE0724	SERP0612	2.330294	960.553	395.447	960.553	395.447	961.641	394.359	1	0	0.0010	0.0000	0.0010	0.0000	–	1.0000	1.0000	0.1587
SE0740	SERP0626	2.330294	398.027	156.973	398.027	156.973	399.116	155.884	1	0	0.0025	0.0000	0.0025	0.0000	–	1.0000	1.0000	0.1587
SE0747	SERP0633	2.330294	836.284	345.716	836.584	345.416	837.522	344.478	1	0	0.0012	0.0000	0.0012	0.0000	–	1.0000	1.0000	0.1587
SE0754	SERP0641	2.330294	858.953	341.047	859.254	340.746	860.192	339.808	1	0	0.0012	0.0000	0.0012	0.0000	–	1.0000	1.0000	0.1587
SE0761	SERP0648	2.330294	606.341	251.659	605.641	252.359	607.079	250.921	1	0	0.0017	0.0000	0.0017	0.0000	–	1.0000	1.0000	0.1587
SE0762	SERP0649	2.330294	345.620	134.380	345.620	134.380	346.708	133.292	1	0	0.0029	0.0000	0.0029	0.0000	–	1.0000	1.0000	0.1587
SE0763	SERP0650	2.330294	805.782	319.218	805.782	319.218	806.870	318.130	1	0	0.0012	0.0000	0.0012	0.0000	–	1.0000	1.0000	0.1587
SE0767	SERP0654	2.330294	1551.626	635.374	1551.325	635.675	1552.564	634.436	1	0	0.0006	0.0000	0.0006	0.0000	–	1.0000	1.0000	0.1587
SE0779	SERP0667	2.330294	830.675	339.325	830.675	339.325	831.763	338.237	1	0	0.0012	0.0000	0.0012	0.0000	–	1.0000	1.0000	0.1587
SE0793	SERP0682	2.330294	926.086	372.914	926.086	372.914	927.174	371.826	1	0	0.0011	0.0000	0.0011	0.0000	–	1.0000	1.0000	0.1587
SE0797	SERP0686	2.330294	783.804	308.196	784.104	307.896	785.042	306.958	1	0	0.0013	0.0000	0.0013	0.0000	–	1.0000	1.0000	0.1587
SE0798	SERP0687	2.330294	570.457	230.543	570.457	230.543	571.546	229.454	1	0	0.0018	0.0000	0.0018	0.0000	–	1.0000	1.0000	0.1587
SE0911	SERP0802	2.330294	232.416	97.584	233.116	96.884	233.854	96.146	1	0	0.0043	0.0000	0.0043	0.0000	–	1.0000	1.0000	0.1587
SE0924	SERP0814	2.330294	644.413	261.587	645.112	260.888	645.851	260.149	1	0	0.0016	0.0000	0.0016	0.0000	–	1.0000	1.0000	0.1587
SE0942	SERP0833	2.330294	863.097	357.903	862.397	358.603	863.835	357.165	1	0	0.0012	0.0000	0.0012	0.0000	–	1.0000	1.0000	0.1587
SE0990	SERP0879	2.330294	971.031	372.969	971.031	372.969	972.119	371.881	1	0	0.0010	0.0000	0.0010	0.0000	–	1.0000	1.0000	0.1587
SE1023	SERP0910	2.330294	169.139	61.861	168.989	62.011	170.152	60.848	1	0	0.0059	0.0000	0.0059	0.0000	–	1.0000	1.0000	0.1587
SE1025	SERP0912	2.330294	1412.937	573.063	1412.937	573.063	1414.025	571.975	1	0	0.0007	0.0000	0.0007	0.0000	–	1.0000	1.0000	0.1587
SE1032	SERP0921	2.330294	1908.027	794.973	1907.727	795.273	1908.965	794.035	1	0	0.0005	0.0000	0.0005	0.0000	–	1.0000	1.0000	0.1587
SE1035	SERP0924	2.330294	429.973	176.027	429.973	176.027	431.061	174.939	1	0	0.0023	0.0000	0.0023	0.0000	–	1.0000	1.0000	0.1587
SE1038	SERP0927	2.330294	1033.453	421.547	1033.453	421.547	1034.541	420.459	1	0	0.0010	0.0000	0.0010	0.0000	–	1.0000	1.0000	0.1587
SE1048	SERP0937	2.330294	1007.393	396.607	1007.393	396.607	1008.481	395.519	1	0	0.0010	0.0000	0.0010	0.0000	–	1.0000	1.0000	0.1587
SE1058	SERP0947	2.330294	894.313	356.687	895.013	355.987	895.751	355.249	1	0	0.0011	0.0000	0.0011	0.0000	–	1.0000	1.0000	0.1587
SE1113	SERP0996	2.330294	1059.924	413.076	1059.774	413.226	1060.938	412.062	1	0	0.0009	0.0000	0.0009	0.0000	–	1.0000	1.0000	0.1587
SE1153	SERP1034	2.330294	927.751	371.249	927.451	371.549	928.689	370.311	1	0	0.0011	0.0000	0.0011	0.0000	–	1.0000	1.0000	0.1587
SE1171	SERP1050	2.330294	687.200	272.800	686.900	273.100	688.138	271.862	1	0	0.0015	0.0000	0.0015	0.0000	–	1.0000	1.0000	0.1587
SE1187	SERP1066	2.330294	658.165	259.835	659.165	258.835	659.754	258.246	1	0	0.0015	0.0000	0.0015	0.0000	–	1.0000	1.0000	0.1587
SE1191	SERP1070	2.330294	1208.553	444.447	1208.703	444.297	1209.716	443.284	1	0	0.0008	0.0000	0.0008	0.0000	–	1.0000	1.0000	0.1587
SE1193	SERP1072	2.330294	804.873	317.127	804.697	317.303	805.873	316.127	1	0	0.0012	0.0000	0.0012	0.0000	–	1.0000	1.0000	0.1587
SE1202	SERP1082	2.330294	627.745	251.255	629.144	249.856	629.533	249.467	1	0	0.0016	0.0000	0.0016	0.0000	–	1.0000	1.0000	0.1587
SE1212	SERP1092	2.330294	727.894	295.106	726.795	296.205	728.433	294.567	1	0	0.0014	0.0000	0.0014	0.0000	–	1.0000	1.0000	0.1587
SE1213	SERP1093	2.330294	394.497	160.503	394.497	160.503	395.586	159.414	1	0	0.0025	0.0000	0.0025	0.0000	–	1.0000	1.0000	0.1587

SE1218	SERP1098	2.330294	275.164	108.836	275.164	108.836	276.253	107.747	1	0	0.0036	0.0000	0.0036	0.0000	–	1.0000	1.0000	0.1587
SE1246	SERP1125	2.330294	793.561	304.439	793.261	304.739	794.499	303.501	1	0	0.0013	0.0000	0.0013	0.0000	–	1.0000	1.0000	0.1587
SE1254	SERP1134	2.330294	653.323	243.677	653.323	243.677	654.412	242.588	1	0	0.0015	0.0000	0.0015	0.0000	–	1.0000	1.0000	0.1587
SE1257	SERP1137	2.330294	335.077	129.923	335.777	129.223	336.515	128.485	1	0	0.0030	0.0000	0.0030	0.0000	–	1.0000	1.0000	0.1587
SE1270	SERP1151	2.330294	804.926	317.074	804.926	317.074	806.014	315.986	1	0	0.0012	0.0000	0.0012	0.0000	–	1.0000	1.0000	0.1587
SE1278	SERP1159	2.330294	254.742	96.258	254.742	96.258	255.830	95.170	1	0	0.0039	0.0000	0.0039	0.0000	–	1.0000	1.0000	0.1587
SE1291	SERP1172	2.330294	528.854	200.146	528.854	200.146	529.942	199.058	1	0	0.0019	0.0000	0.0019	0.0000	–	1.0000	1.0000	0.1587
SE1306	SERP1187	2.330294	702.417	290.583	702.417	290.583	703.506	289.494	1	0	0.0014	0.0000	0.0014	0.0000	–	1.0000	1.0000	0.1587
SE1313	SERP1194	2.330294	623.910	249.090	623.910	249.090	624.998	248.002	1	0	0.0016	0.0000	0.0016	0.0000	–	1.0000	1.0000	0.1587
SE1320	SERP1201	2.330294	1614.827	674.173	1615.127	673.873	1616.065	672.935	1	0	0.0006	0.0000	0.0006	0.0000	–	1.0000	1.0000	0.1587
SE1327	SERP1208	2.330294	914.910	375.090	914.511	375.489	915.799	374.201	1	0	0.0011	0.0000	0.0011	0.0000	–	1.0000	1.0000	0.1587
SE1337	SERP1226	2.330294	502.642	205.358	501.642	206.358	503.231	204.769	1	0	0.0020	0.0000	0.0020	0.0000	–	1.0000	1.0000	0.1587
SE1346	SERP1235	2.330294	577.988	232.012	577.688	232.312	578.926	231.074	1	0	0.0017	0.0000	0.0017	0.0000	–	1.0000	1.0000	0.1587
SE1347	SERP1236	2.330294	951.237	392.763	951.237	392.763	952.325	391.675	1	0	0.0011	0.0000	0.0011	0.0000	–	1.0000	1.0000	0.1587
SE1352	SERP1241	2.330294	441.704	167.296	441.404	167.596	442.642	166.358	1	0	0.0023	0.0000	0.0023	0.0000	–	1.0000	1.0000	0.1587
SE1357	SERP1246	2.330294	1392.464	542.536	1392.464	542.536	1393.552	541.448	1	0	0.0007	0.0000	0.0007	0.0000	–	1.0000	1.0000	0.1587
SE1365	SERP1252	2.330294	439.454	169.546	439.454	169.546	440.542	168.458	1	0	0.0023	0.0000	0.0023	0.0000	–	1.0000	1.0000	0.1587
SE1388	SERP1276	2.330294	674.076	270.924	674.376	270.624	675.314	269.686	1	0	0.0015	0.0000	0.0015	0.0000	–	1.0000	1.0000	0.1587
SE1393	SERP1281	2.330294	1213.423	478.577	1213.247	478.753	1214.423	477.577	1	0	0.0008	0.0000	0.0008	0.0000	–	1.0000	1.0000	0.1587
SE1446	SERP1333	2.330294	341.751	135.249	341.574	135.426	342.751	134.249	1	0	0.0029	0.0000	0.0029	0.0000	–	1.0000	1.0000	0.1587
SE1465	SERP1359	2.330294	101.236	42.764	102.236	41.764	102.824	41.176	1	0	0.0098	0.0000	0.0099	0.0000	–	1.0000	1.0000	0.1587
SE1519	SERP1374	2.330294	266.669	102.331	266.669	102.331	267.757	101.243	1	0	0.0037	0.0000	0.0038	0.0000	–	1.0000	1.0000	0.1587
SE1526	SERP1381	2.330294	252.196	89.804	252.196	89.804	253.285	88.715	1	0	0.0040	0.0000	0.0040	0.0000	–	1.0000	1.0000	0.1587
SE1530	SERP1385	2.330294	806.130	324.870	805.830	325.170	807.068	323.932	1	0	0.0012	0.0000	0.0012	0.0000	–	1.0000	1.0000	0.1587
SE1541	SERP1395	2.330294	1040.239	414.761	1039.939	415.061	1041.177	413.823	1	0	0.0010	0.0000	0.0010	0.0000	–	1.0000	1.0000	0.1587
SE1543	SERP1397	2.330294	609.828	236.172	609.828	236.172	610.916	235.084	1	0	0.0016	0.0000	0.0016	0.0000	–	1.0000	1.0000	0.1587
SE1547	SERP1400	2.330294	331.548	124.452	330.848	125.152	332.286	123.714	1	0	0.0030	0.0000	0.0030	0.0000	–	1.0000	1.0000	0.1587
SE1553	SERP1407	2.330294	697.163	274.837	697.463	274.537	698.401	273.599	1	0	0.0014	0.0000	0.0014	0.0000	–	1.0000	1.0000	0.1587
SE1565	SERP1418	2.330294	157.408	55.592	157.108	55.892	158.346	54.654	1	0	0.0064	0.0000	0.0064	0.0000	–	1.0000	1.0000	0.1587
SE1568	SERP1421	2.330294	833.530	342.470	833.530	342.470	834.618	341.382	1	0	0.0012	0.0000	0.0012	0.0000	–	1.0000	1.0000	0.1587
SE1578	SERP1431	2.330294	356.780	141.220	357.080	140.920	358.018	139.982	1	0	0.0028	0.0000	0.0028	0.0000	–	1.0000	1.0000	0.1587
SE1590	SERP1443	2.330294	1575.717	611.283	1575.717	611.283	1576.805	610.195	1	0	0.0006	0.0000	0.0006	0.0000	–	1.0000	1.0000	0.1587
SE1600	SERP1453	2.330294	787.576	307.424	787.876	307.124	788.814	306.186	1	0	0.0013	0.0000	0.0013	0.0000	–	1.0000	1.0000	0.1587
SE1601	SERP1454	2.330294	395.205	156.795	395.205	156.795	396.293	155.707	1	0	0.0025	0.0000	0.0025	0.0000	–	1.0000	1.0000	0.1587
SE1602	SERP1455	2.330294	662.297	264.703	661.997	265.003	663.235	263.765	1	0	0.0015	0.0000	0.0015	0.0000	–	1.0000	1.0000	0.1587
SE1631	SERP1486	2.330294	529.855	208.145	529.855	208.145	530.943	207.057	1	0	0.0019	0.0000	0.0019	0.0000	–	1.0000	1.0000	0.1587
SE1644	SERP1498	2.330294	895.974	352.026	895.974	352.026	897.062	350.938	1	0	0.0011	0.0000	0.0011	0.0000	–	1.0000	1.0000	0.1587
SE1647	SERP1657	2.330294	452.701	180.299	452.002	180.998	453.440	179.560	1	0	0.0022	0.0000	0.0022	0.0000	–	1.0000	1.0000	0.1587
SE1649	SERP1660	2.330294	1157.285	456.715	1157.162	456.838	1158.312	455.688	1	0	0.0009	0.0000	0.0009	0.0000	–	1.0000	1.0000	0.1587

SE1659	SERP1670	2.330294	729.525	311.475	729.525	311.475	730.614	310.386	1	0	0.0014	0.0000	0.0014	0.0000	–	1.0000	1.0000	0.1587
SE1678	SERP1687	2.330294	339.669	137.331	339.669	137.331	340.757	136.243	1	0	0.0029	0.0000	0.0029	0.0000	–	1.0000	1.0000	0.1587
SE1689	SERP1697	2.330294	631.222	238.778	631.222	238.778	632.310	237.690	1	0	0.0016	0.0000	0.0016	0.0000	–	1.0000	1.0000	0.1587
SE1695	SERP1703	2.330294	286.692	106.308	286.843	106.157	287.856	105.144	1	0	0.0035	0.0000	0.0035	0.0000	–	1.0000	1.0000	0.1587
SE1699	SERP1708	2.330294	285.961	116.039	285.961	116.039	287.049	114.951	1	0	0.0035	0.0000	0.0035	0.0000	–	1.0000	1.0000	0.1587
SE1722	SERP1731	2.330294	892.726	364.274	892.903	364.097	893.902	363.098	1	0	0.0011	0.0000	0.0011	0.0000	–	1.0000	1.0000	0.1587
SE1733	SERP1742	2.330294	241.211	106.789	241.061	106.939	242.224	105.776	1	0	0.0041	0.0000	0.0042	0.0000	–	1.0000	1.0000	0.1587
SE1738	SERP1748	2.330294	322.175	121.825	322.175	121.825	323.263	120.737	1	0	0.0031	0.0000	0.0031	0.0000	–	1.0000	1.0000	0.1587
SE1761	SERP1770	2.330294	846.640	338.360	846.640	338.360	847.729	337.271	1	0	0.0012	0.0000	0.0012	0.0000	–	1.0000	1.0000	0.1587
SE1770	SERP1779	2.330294	1256.811	498.189	1256.961	498.039	1257.974	497.026	1	0	0.0008	0.0000	0.0008	0.0000	–	1.0000	1.0000	0.1587
SE1777	SERP1785	2.330294	723.623	284.377	723.623	284.377	724.711	283.289	1	0	0.0014	0.0000	0.0014	0.0000	–	1.0000	1.0000	0.1587
SE1789	SERP1797	2.330294	530.915	207.085	530.614	207.386	531.853	206.147	1	0	0.0019	0.0000	0.0019	0.0000	–	1.0000	1.0000	0.1587
SE1830	SERP1839	2.330294	570.006	218.994	570.307	218.693	571.245	217.755	1	0	0.0018	0.0000	0.0018	0.0000	–	1.0000	1.0000	0.1587
SE1832	SERP1841	2.330294	119.361	48.639	119.061	48.939	120.299	47.701	1	0	0.0084	0.0000	0.0084	0.0000	–	1.0000	1.0000	0.1587
SE1836	SERP1845	2.330294	549.383	209.617	549.683	209.317	550.621	208.379	1	0	0.0018	0.0000	0.0018	0.0000	–	1.0000	1.0000	0.1587
SE1857	SERP1865	2.330294	671.570	267.430	671.720	267.280	672.733	266.267	1	0	0.0015	0.0000	0.0015	0.0000	–	1.0000	1.0000	0.1587
SE1865	SERP1873	2.330294	495.659	191.341	495.659	191.341	496.747	190.253	1	0	0.0020	0.0000	0.0020	0.0000	–	1.0000	1.0000	0.1587
SE1884	SERP1894	2.330294	588.660	224.340	588.660	224.340	589.748	223.252	1	0	0.0017	0.0000	0.0017	0.0000	–	1.0000	1.0000	0.1587
SE1891	SERP1901	2.330294	617.396	252.604	617.396	252.604	618.484	251.516	1	0	0.0016	0.0000	0.0016	0.0000	–	1.0000	1.0000	0.1587
SE1895	SERP1907	2.330294	452.936	180.064	452.936	180.064	454.024	178.976	1	0	0.0022	0.0000	0.0022	0.0000	–	1.0000	1.0000	0.1587
SE1914	SERP1927	2.330294	1154.297	453.703	1153.996	454.004	1155.235	452.765	1	0	0.0009	0.0000	0.0009	0.0000	–	1.0000	1.0000	0.1587
SE1920	SERP1932	2.330294	581.418	219.582	581.268	219.732	582.431	218.569	1	0	0.0017	0.0000	0.0017	0.0000	–	1.0000	1.0000	0.1587
SE1976	SERP1988	2.330294	662.172	270.828	662.172	270.828	663.260	269.740	1	0	0.0015	0.0000	0.0015	0.0000	–	1.0000	1.0000	0.1587
SE1979	SERP1992	2.330294	512.304	204.696	512.304	204.696	513.393	203.607	1	0	0.0020	0.0000	0.0020	0.0000	–	1.0000	1.0000	0.1587
SE1993	SERP2005	2.330294	561.913	224.087	561.913	224.087	563.001	222.999	1	0	0.0018	0.0000	0.0018	0.0000	–	1.0000	1.0000	0.1587
SE1995	SERP2007	2.330294	484.083	199.917	484.383	199.617	485.321	198.679	1	0	0.0021	0.0000	0.0021	0.0000	–	1.0000	1.0000	0.1587
SE2015	SERP2028	2.330294	488.001	204.999	487.701	205.299	488.939	204.061	1	0	0.0020	0.0000	0.0021	0.0000	–	1.0000	1.0000	0.1587
SE2045	SERP2058	2.330294	1098.445	440.555	1098.745	440.255	1099.683	439.317	1	0	0.0009	0.0000	0.0009	0.0000	–	1.0000	1.0000	0.1587
SE2051	SERP2064	2.330294	656.352	258.648	656.175	258.825	657.351	257.649	1	0	0.0015	0.0000	0.0015	0.0000	–	1.0000	1.0000	0.1587
SE2060	SERP2073	2.330294	537.186	227.814	537.186	227.814	538.274	226.726	1	0	0.0019	0.0000	0.0019	0.0000	–	1.0000	1.0000	0.1587
SE2073	SERP2086	2.330294	475.381	184.619	475.682	184.318	476.620	183.380	1	0	0.0021	0.0000	0.0021	0.0000	–	1.0000	1.0000	0.1587
SE2090	SERP2104	2.330294	123.660	50.340	123.960	50.040	124.898	49.102	1	0	0.0081	0.0000	0.0081	0.0000	–	1.0000	1.0000	0.1587
SE2110	SERP2122	2.330294	833.730	330.270	833.430	330.570	834.668	329.332	1	0	0.0012	0.0000	0.0012	0.0000	–	1.0000	1.0000	0.1587
SE2121	SERP2133	2.330294	710.616	285.384	710.316	285.684	711.554	284.446	1	0	0.0014	0.0000	0.0014	0.0000	–	1.0000	1.0000	0.1587
SE2125	SERP2137	2.330294	1293.172	512.828	1293.172	512.828	1294.260	511.740	1	0	0.0008	0.0000	0.0008	0.0000	–	1.0000	1.0000	0.1587
SE2129	SERP2141	2.330294	751.211	304.789	751.911	304.089	752.649	303.351	1	0	0.0013	0.0000	0.0013	0.0000	–	1.0000	1.0000	0.1587
SE2131	SERP2143	2.330294	613.459	241.541	613.609	241.391	614.622	240.378	1	0	0.0016	0.0000	0.0016	0.0000	–	1.0000	1.0000	0.1587
SE2135	SERP2147	2.330294	517.221	196.779	517.221	196.779	518.309	195.691	1	0	0.0019	0.0000	0.0019	0.0000	–	1.0000	1.0000	0.1587
SE2165	SERP2176	2.330294	1226.208	489.792	1226.058	489.942	1227.221	488.779	1	0	0.0008	0.0000	0.0008	0.0000	–	1.0000	1.0000	0.1587

SE2174	SERP2185	2.330294	427.575	169.425	427.875	169.125	428.813	168.187	1	0	0.0023	0.0000	0.0023	0.0000	-	1.0000	1.0000	0.1587
SE2179	SERP2190	2.330294	1228.258	487.742	1228.258	487.742	1229.346	486.654	1	0	0.0008	0.0000	0.0008	0.0000	-	1.0000	1.0000	0.1587
SE2180	SERP2191	2.330294	1314.062	527.938	1313.912	528.088	1315.075	526.925	1	0	0.0008	0.0000	0.0008	0.0000	-	1.0000	1.0000	0.1587
SE2181	SERP2192	2.330294	524.643	204.357	524.643	204.357	525.731	203.269	1	0	0.0019	0.0000	0.0019	0.0000	-	1.0000	1.0000	0.1587
SE2240	SERP2272	2.330294	342.679	134.321	342.679	134.321	343.767	133.233	1	0	0.0029	0.0000	0.0029	0.0000	-	1.0000	1.0000	0.1587
SE2243	SERP2275	2.330294	1077.919	428.081	1077.919	428.081	1079.007	426.993	1	0	0.0009	0.0000	0.0009	0.0000	-	1.0000	1.0000	0.1587
SE2253	SERP2283	2.330294	575.284	237.716	574.434	238.566	575.947	237.053	1	0	0.0017	0.0000	0.0017	0.0000	-	1.0000	1.0000	0.1587
SE0261	SERP2317	2.330294	454.230	181.770	453.530	182.470	454.968	181.032	1	0	0.0022	0.0000	0.0022	0.0000	-	1.0000	1.0000	0.1587
SE0228	SERP2352	2.330294	657.696	272.304	657.696	272.304	658.784	271.216	1	0	0.0015	0.0000	0.0015	0.0000	-	1.0000	1.0000	0.1587
SE0011	SERP2541	2.330294	682.245	283.755	681.945	284.055	683.183	282.817	1	0	0.0015	0.0000	0.0015	0.0000	-	1.0000	1.0000	0.1587
SE1206	SERP1086	2.330294	256.442	103.558	256.141	103.859	256.792	103.208	2	0	0.0078	0.0000	0.0078	0.0000	-	1.0000	0.9873	0.1617
SE1998	SERP2010	2.330294	1064.360	405.640	1063.811	406.189	1065.174	404.826	6	1	0.0056	0.0025	0.0057	0.0025	2.2800	0.6951	0.9420	0.1731
SE2163	SERP2174	2.330294	154.088	61.912	152.565	63.435	154.415	61.585	5	1	0.0326	0.0160	0.0333	0.0161	2.0683	0.6741	0.7941	0.2136
SE1730	SERP1739	2.330294	847.384	340.616	848.184	339.816	848.872	339.128	5	1	0.0059	0.0029	0.0059	0.0029	2.0345	0.6705	0.7702	0.2206
SE1378	SERP1266	2.330294	2291.086	903.914	2291.387	903.613	2292.325	902.675	5	1	0.0022	0.0011	0.0022	0.0011	2.0000	0.6667	0.7399	0.2297
SE1020	SERP0907	2.330294	736.021	286.979	738.447	284.553	738.322	284.678	5	1	0.0068	0.0035	0.0068	0.0035	1.9429	0.6602	0.7159	0.2370
SE1429	SERP1316	2.330294	7983.041	3092.959	7984.041	3091.959	7984.629	3091.371	14	4	0.0018	0.0013	0.0018	0.0013	1.3846	0.5806	0.6402	0.2610
SE0601	SERP0490	2.330294	485.697	207.303	486.148	206.852	487.011	205.989	4	1	0.0082	0.0048	0.0083	0.0048	1.7292	0.6336	0.5544	0.2896
SE2097	SERP2111	2.330294	234.267	89.733	234.868	89.132	234.567	89.433	2.5	0.5	0.0107	0.0056	0.0107	0.0056	1.9107	0.6564	0.5357	0.2961
SE2415	SERP0005	2.330294	507.535	209.465	506.835	210.165	508.273	208.727	4	1	0.0079	0.0048	0.0079	0.0048	1.6458	0.6220	0.5012	0.3081
SE2408	SERP0013	2.330294	451.202	175.798	451.301	175.699	452.340	174.660	4	1	0.0089	0.0057	0.0089	0.0057	1.5614	0.6096	0.4444	0.3284
SE2013	SERP2026	2.330294	317.426	138.574	318.126	137.874	318.864	137.136	3	1	0.0094	0.0072	0.0095	0.0073	1.3014	0.5655	0.2407	0.4049
SE1514	SERP1369	2.330294	356.152	144.848	355.675	145.325	357.002	143.998	3	1	0.0084	0.0069	0.0085	0.0069	1.2319	0.5519	0.1891	0.4250
SE2186	SERP2197	2.330294	540.390	206.610	540.089	206.911	541.328	205.672	6	2	0.0111	0.0097	0.0112	0.0097	1.1546	0.5359	0.1821	0.4278
SE1963	SERP1974	2.330294	259.343	88.657	259.193	88.807	260.356	87.644	3	1	0.0116	0.0113	0.0117	0.0114	1.0263	0.5065	0.0227	0.4909
SE2007	SERP2019	2.330294	1085.077	429.923	1084.677	430.323	1085.965	429.035	5	2	0.0046	0.0046	0.0046	0.0047	0.9787	0.4946	-0.0256	0.5102
SE1598	SERP1451	2.330294	767.049	297.951	767.499	297.501	767.274	297.726	5	2	0.0065	0.0067	0.0065	0.0067	0.9701	0.4924	-0.0337	0.5134
SE2338	SERP0077	2.330294	83.753	30.247	84.103	29.897	85.016	28.984	5	2	0.0596	0.0665	0.0621	0.0696	0.8922	0.4715	-0.1333	0.5530
SE1011	SERP0899	2.330294	648.610	269.390	648.911	269.089	649.849	268.151	2	1	0.0031	0.0037	0.0031	0.0037	0.8378	0.4559	-0.1394	0.5554
SE0653	SERP0545	2.330294	836.559	351.441	836.859	351.141	837.797	350.203	2	1	0.0024	0.0028	0.0024	0.0029	0.8276	0.4528	-0.1487	0.5591
SE0573	SERP0460	2.330294	242.663	99.337	242.363	99.637	243.601	98.399	2	1	0.0082	0.0101	0.0083	0.0101	0.8218	0.4511	-0.1545	0.5614
SE1405	SERP1292	2.330294	883.725	352.275	883.124	352.876	884.512	351.488	2	1	0.0023	0.0028	0.0023	0.0028	0.8214	0.4510	-0.1550	0.5616
SE2086	SERP2100	2.330294	654.620	266.380	655.719	265.281	656.258	264.742	2	1	0.0031	0.0038	0.0031	0.0038	0.8158	0.4493	-0.1594	0.5633
SE0203	SERP2373	2.330294	915.791	374.209	917.014	372.986	917.491	372.509	2	1	0.0022	0.0027	0.0022	0.0027	0.8148	0.4490	-0.1619	0.5643
SE0391	SERP0275	2.330294	676.998	274.002	676.698	274.302	677.936	273.064	2	1	0.0030	0.0036	0.0030	0.0037	0.8108	0.4478	-0.1645	0.5653
SE1204	SERP1084	2.330294	956.714	378.286	956.714	378.286	957.802	377.198	2	1	0.0021	0.0026	0.0021	0.0026	0.8077	0.4468	-0.1666	0.5661
SE0470	SERP0357	2.330294	538.312	217.688	538.312	217.688	539.400	216.600	2	1	0.0037	0.0046	0.0037	0.0046	0.8043	0.4458	-0.1703	0.5676
SE1984	SERP1996	2.330294	381.323	152.677	381.323	152.677	382.411	151.589	2	1	0.0052	0.0065	0.0053	0.0066	0.8030	0.4454	-0.1718	0.5682
SE1978	SERP1990	2.330294	1714.292	688.708	1713.991	689.009	1715.230	687.770	2	1	0.0012	0.0015	0.0012	0.0015	0.8000	0.4444	-0.1765	0.5700

SE0040	SERP2494	2.330294	758.909	285.091	758.732	285.268	758.820	285.180	7	3	0.0092	0.0105	0.0093	0.0106	0.8774	0.4673	−0.1782	0.5707
SE1997	SERP2009	2.330294	275.090	108.910	275.090	108.910	276.179	107.821	2	1	0.0073	0.0092	0.0073	0.0092	0.7935	0.4424	−0.1783	0.5708
SE0090	SERP2526	2.330294	481.608	190.392	480.807	191.193	482.296	189.704	2	1	0.0042	0.0052	0.0042	0.0053	0.7925	0.4421	−0.1821	0.5722
SE1198	SERP1078	2.330294	714.567	281.433	715.067	280.933	715.905	280.095	2	1	0.0028	0.0036	0.0028	0.0036	0.7778	0.4375	−0.1943	0.5770
SE1466	SERP1360	2.330294	476.856	186.144	476.856	186.144	477.944	185.056	2	1	0.0042	0.0054	0.0042	0.0054	0.7778	0.4375	−0.1943	0.5770
SE2410	SERP0011	2.330294	389.074	147.926	389.074	147.926	390.162	146.838	2	1	0.0051	0.0068	0.0052	0.0068	0.7647	0.4333	−0.2080	0.5824
SE0413	SERP0298	2.330294	787.949	301.051	787.399	301.601	788.762	300.238	2	1	0.0025	0.0033	0.0025	0.0033	0.7576	0.4310	−0.2128	0.5843
SE1727	SERP1736	2.330294	610.972	247.028	611.122	246.878	612.135	245.865	4	2	0.0065	0.0081	0.0066	0.0081	0.8148	0.4490	−0.2248	0.5889
SE0166	SERP2405	2.330294	1097.445	438.555	1098.895	437.105	1099.258	436.742	8	4	0.0073	0.0091	0.0073	0.0092	0.7935	0.4424	−0.3596	0.6404
SE2254	SERP2284	2.330294	561.086	236.914	561.086	236.914	562.174	235.826	3	2	0.0053	0.0084	0.0054	0.0085	0.6353	0.3885	−0.4590	0.6769
SE1051	SERP0940	2.330294	554.396	231.604	555.646	230.354	556.109	229.891	3	2	0.0054	0.0087	0.0054	0.0087	0.6207	0.3830	−0.4761	0.6830
SE1037	SERP0926	2.330294	1709.536	690.464	1709.713	690.287	1710.713	689.287	1	1	0.0006	0.0014	0.0006	0.0014	0.4286	0.3000	−0.4952	0.6898
SE2381	SERP0035	2.330294	1294.941	541.059	1294.641	541.359	1295.879	540.121	1	1	0.0008	0.0018	0.0008	0.0018	0.4444	0.3077	−0.5077	0.6942
SE0750	SERP0636	2.330294	2845.868	1159.132	2846.568	1158.432	2847.306	1157.694	1	1	0.0004	0.0009	0.0004	0.0009	0.4444	0.3077	−0.5077	0.6942
SE1804	SERP1812	2.330294	303.794	134.206	304.494	133.506	305.232	132.768	1	1	0.0033	0.0075	0.0033	0.0075	0.4400	0.3056	−0.5126	0.6959
SE1194	SERP1074	2.330294	688.756	286.244	688.456	286.544	689.694	285.306	1	1	0.0015	0.0035	0.0015	0.0035	0.4286	0.3000	−0.5252	0.7003
SE0151	SERP2419	2.330294	132.232	50.768	133.333	49.667	132.783	50.217	3	2	0.0226	0.0398	0.0229	0.0409	0.5599	0.3589	−0.5333	0.7031
SE2172	SERP2183	2.330294	1313.650	534.350	1313.350	534.650	1314.588	533.412	1	1	0.0008	0.0019	0.0008	0.0019	0.4211	0.2963	−0.5336	0.7032
SE0784	SERP0672	2.330294	968.402	384.598	969.351	383.649	969.965	383.035	5	3	0.0052	0.0078	0.0052	0.0079	0.6582	0.3969	−0.5343	0.7034
SE1728	SERP1737	2.330294	561.058	233.942	561.058	233.942	562.146	232.854	1	1	0.0018	0.0043	0.0018	0.0043	0.4186	0.2951	−0.5363	0.7041
SE2010	SERP2023	2.330294	974.909	423.091	975.059	422.941	976.072	421.928	1	1	0.0010	0.0024	0.0010	0.0024	0.4167	0.2941	−0.5385	0.7049
SE2183	SERP2194	2.330294	336.981	137.019	337.282	136.718	338.220	135.780	1	1	0.0030	0.0073	0.0030	0.0073	0.4110	0.2913	−0.5385	0.7049
SE1456	SERP1350	2.330294	602.855	231.145	602.405	231.595	602.630	231.370	3	2	0.0050	0.0086	0.0050	0.0087	0.5747	0.3650	−0.5406	0.7056
SE0745	SERP0631	2.330294	570.591	230.409	570.891	230.109	571.829	229.171	1	1	0.0018	0.0043	0.0018	0.0044	0.4091	0.2903	−0.5469	0.7078
SE1691	SERP1699	2.330294	555.890	230.110	556.191	229.809	557.129	228.871	1	1	0.0018	0.0043	0.0018	0.0044	0.4091	0.2903	−0.5469	0.7078
SE0504	SERP0387	2.330294	741.778	311.222	741.602	311.398	742.778	310.222	1	1	0.0013	0.0032	0.0013	0.0032	0.4063	0.2889	−0.5501	0.7089
SE1868	SERP1876	2.330294	243.520	98.480	243.520	98.480	244.608	97.392	1	1	0.0041	0.0102	0.0041	0.0102	0.4020	0.2867	−0.5502	0.7089
SE1883	SERP1893	2.330294	679.663	268.337	678.263	269.737	680.051	267.949	1	1	0.0015	0.0037	0.0015	0.0037	0.4054	0.2885	−0.5510	0.7092
SE0544	SERP0429	2.330294	600.427	236.573	599.027	237.973	600.815	236.185	1	1	0.0017	0.0042	0.0017	0.0042	0.4048	0.2881	−0.5518	0.7094
SE1596	SERP1449	2.330294	585.487	239.513	585.487	239.513	586.575	238.425	1	1	0.0017	0.0042	0.0017	0.0042	0.4048	0.2881	−0.5518	0.7094
SE0610	SERP0500	2.330294	999.816	395.184	1000.117	394.883	1001.055	393.945	1	1	0.0010	0.0025	0.0010	0.0025	0.4000	0.2857	−0.5571	0.7113
SE1453	SERP0544	2.330294	725.375	285.625	725.375	285.625	726.463	284.537	1	1	0.0014	0.0035	0.0014	0.0035	0.4000	0.2857	−0.5571	0.7113
SE1197	SERP1077	2.330294	693.493	287.507	693.493	287.507	694.581	286.419	1	1	0.0014	0.0035	0.0014	0.0035	0.4000	0.2857	−0.5571	0.7113
SE1361	SERP1250	2.330294	733.866	289.134	733.566	289.434	734.804	288.196	1	1	0.0014	0.0035	0.0014	0.0035	0.4000	0.2857	−0.5571	0.7113
SE1784	SERP1792	2.330294	690.904	284.096	691.081	283.919	692.080	282.920	1	1	0.0014	0.0035	0.0014	0.0035	0.4000	0.2857	−0.5571	0.7113
SE1975	SERP1987	2.330294	2653.185	1027.815	2653.185	1027.815	2654.274	1026.726	1	1	0.0004	0.0010	0.0004	0.0010	0.4000	0.2857	−0.5571	0.7113
SE2244	SERP2276	2.330294	1711.663	676.337	1711.963	676.037	1712.901	675.099	1	1	0.0006	0.0015	0.0006	0.0015	0.4000	0.2857	−0.5571	0.7113
SE0647	SERP0539	2.330294	269.245	108.755	269.245	108.755	270.333	107.667	1	1	0.0037	0.0092	0.0037	0.0093	0.3978	0.2846	−0.5595	0.7121
SE1711	SERP1720	2.330294	374.648	147.352	374.798	147.202	375.811	146.189	1	1	0.0027	0.0068	0.0027	0.0068	0.3971	0.2842	−0.5604	0.7124

SE0602	SERP0491	2.330294	574.474	235.526	574.474	235.526	575.562	234.438	1	1	0.0017	0.0042	0.0017	0.0043	0.3953	0.2833	-0.5623	0.7130
SE0746	SERP0632	2.330294	580.210	235.790	580.910	235.090	581.648	234.352	1	1	0.0017	0.0042	0.0017	0.0043	0.3953	0.2833	-0.5623	0.7130
SE0209	SERP2369	2.330294	580.342	232.658	579.642	233.358	581.080	231.920	1	1	0.0017	0.0043	0.0017	0.0043	0.3953	0.2833	-0.5623	0.7130
SE0414	SERP0299	2.330294	762.345	299.655	762.646	299.354	763.584	298.416	1	1	0.0013	0.0033	0.0013	0.0033	0.3939	0.2826	-0.5639	0.7136
SE1010	SERP0898	2.330294	756.820	305.180	756.121	305.879	757.559	304.441	1	1	0.0013	0.0033	0.0013	0.0033	0.3939	0.2826	-0.5639	0.7136
SE2098	SERP2112	2.330294	742.308	307.692	743.707	306.293	744.095	305.905	1	1	0.0013	0.0033	0.0013	0.0033	0.3939	0.2826	-0.5639	0.7136
SE2347	SERP0070	2.330294	1100.299	438.701	1099.749	439.251	1101.112	437.888	1	1	0.0009	0.0023	0.0009	0.0023	0.3913	0.2813	-0.5668	0.7146
SE1014	SERP0901	2.330294	224.338	87.662	224.639	87.361	225.577	86.423	1	1	0.0045	0.0114	0.0045	0.0115	0.3913	0.2813	-0.5668	0.7146
SE1220	SERP1100	2.330294	1073.890	432.110	1073.890	432.110	1074.979	431.021	1	1	0.0009	0.0023	0.0009	0.0023	0.3913	0.2813	-0.5668	0.7146
SE1679	SERP1688	2.330294	1094.357	432.643	1094.357	432.643	1095.445	431.555	1	1	0.0009	0.0023	0.0009	0.0023	0.3913	0.2813	-0.5668	0.7146
SE2198	SERP2209	2.330294	1101.113	440.887	1101.413	440.587	1102.351	439.649	1	1	0.0009	0.0023	0.0009	0.0023	0.3913	0.2813	-0.5668	0.7146
SE2219	SERP2252	2.330294	1087.242	433.758	1087.242	433.758	1088.330	432.670	1	1	0.0009	0.0023	0.0009	0.0023	0.3913	0.2813	-0.5668	0.7146
SE1612	SERP1465	2.330294	1464.447	560.553	1463.897	561.103	1465.260	559.740	1	1	0.0007	0.0018	0.0007	0.0018	0.3889	0.2800	-0.5696	0.7155
SE0156	SERP2413	2.330294	696.045	281.955	696.045	281.955	697.133	280.867	1	1	0.0014	0.0035	0.0014	0.0036	0.3889	0.2800	-0.5696	0.7155
SE0505	SERP0388	2.330294	390.423	146.577	390.122	146.878	391.361	145.639	1	1	0.0026	0.0068	0.0026	0.0068	0.3824	0.2766	-0.5696	0.7155
SE0825	SERP0716	2.330294	807.532	323.468	807.532	323.468	808.621	322.379	1	1	0.0012	0.0031	0.0012	0.0031	0.3871	0.2791	-0.5716	0.7162
SE1985	SERP1997	2.330294	589.743	229.257	589.743	229.257	590.831	228.169	1	1	0.0017	0.0044	0.0017	0.0044	0.3864	0.2787	-0.5724	0.7165
SE0110	SERP2220	2.330294	308.369	120.631	308.369	120.631	309.457	119.543	1	1	0.0032	0.0083	0.0032	0.0083	0.3855	0.2783	-0.5733	0.7168
SE2299	SERP0122	2.330294	666.800	257.200	666.800	257.200	667.888	256.112	1	1	0.0015	0.0039	0.0015	0.0039	0.3846	0.2778	-0.5744	0.7171
SE1372	SERP1260	2.330294	965.851	390.149	965.851	390.149	966.939	389.061	1	1	0.0010	0.0026	0.0010	0.0026	0.3846	0.2778	-0.5744	0.7171
SE1660	SERP1671	2.330294	980.835	387.165	980.835	387.165	981.924	386.076	1	1	0.0010	0.0026	0.0010	0.0026	0.3846	0.2778	-0.5744	0.7171
SE0251	SERP2330	2.330294	649.922	259.078	649.222	259.778	650.661	258.339	1	1	0.0015	0.0039	0.0015	0.0039	0.3846	0.2778	-0.5744	0.7171
SE1949	SERP1961	2.330294	388.694	148.306	388.571	148.429	389.721	147.279	1	1	0.0026	0.0067	0.0026	0.0068	0.3824	0.2766	-0.5769	0.7180
SE2091	SERP2105	2.330294	772.900	298.100	772.577	298.423	773.827	297.173	1	1	0.0013	0.0034	0.0013	0.0034	0.3824	0.2766	-0.5769	0.7180
SE0786	SERP0674	2.330294	473.548	183.452	473.548	183.452	474.636	182.364	1	1	0.0021	0.0055	0.0021	0.0055	0.3818	0.2763	-0.5775	0.7182
SE1196	SERP1076	2.330294	950.617	366.383	950.740	366.260	951.767	365.233	7	4	0.0074	0.0109	0.0074	0.0110	0.6727	0.4022	-0.5833	0.7202
SE1188	SERP1067	2.330294	1065.695	416.305	1065.695	416.305	1066.784	415.216	1	1	0.0009	0.0024	0.0009	0.0024	0.3750	0.2727	-0.5852	0.7208
SE1552	SERP1406	2.330294	755.650	285.350	755.650	285.350	756.739	284.261	1	1	0.0013	0.0035	0.0013	0.0035	0.3714	0.2708	-0.5892	0.7221
SE0574	SERP0461	2.330294	302.675	111.325	302.675	111.325	303.763	110.237	1	1	0.0033	0.0090	0.0033	0.0090	0.3667	0.2683	-0.5946	0.7240
SE1432	SERP1319	2.330294	875.903	336.097	875.603	336.397	876.841	335.159	1	1	0.0011	0.0030	0.0011	0.0030	0.3667	0.2683	-0.5946	0.7240
SE2201	SERP2212	2.330294	946.144	388.856	948.044	386.956	947.094	387.906	15.5	8.5	0.0164	0.0219	0.0165	0.0222	0.7432	0.4264	-0.6528	0.7430
SE0181	SERP2394	2.330294	805.211	325.789	805.785	325.215	805.498	325.502	15.5	8.5	0.0192	0.0261	0.0195	0.0266	0.7331	0.4230	-0.6798	0.7517
SE2239	SERP2271	2.330294	243.947	110.053	243.947	110.053	245.035	108.965	2	2	0.0082	0.0182	0.0082	0.0184	0.4457	0.3083	-0.7120	0.7618
SE1464	SERP1358	2.330294	1013.025	408.975	1014.025	407.975	1014.613	407.387	6	4	0.0059	0.0098	0.0059	0.0099	0.5960	0.3734	-0.7331	0.7683
SE1459	SERP1353	2.330294	1137.876	452.124	1140.126	449.874	1140.089	449.911	4	3	0.0035	0.0067	0.0035	0.0067	0.5224	0.3431	-0.7450	0.7719
SE0003	SERP2550	2.330294	785.684	327.316	786.484	326.516	787.172	325.828	2	2	0.0025	0.0061	0.0025	0.0061	0.4098	0.2907	-0.7573	0.7756
SE1628	SERP1483	2.330294	772.850	298.150	773.099	297.901	774.063	296.937	6	4	0.0078	0.0134	0.0078	0.0135	0.5778	0.3662	-0.7585	0.7759
SE1437	SERP1324	2.330294	720.889	278.111	720.340	278.660	721.703	277.297	4	3	0.0056	0.0108	0.0056	0.0109	0.5138	0.3394	-0.7688	0.7790
SE2152	SERP2162	2.330294	415.503	172.497	414.703	173.297	415.103	172.897	2	2	0.0048	0.0116	0.0048	0.0117	0.4103	0.2909	-0.7693	0.7791

SE1589	SERP1442	2.330294	1418.042	576.958	1418.418	576.582	1419.318	575.682	2	2	0.0014	0.0035	0.0014	0.0035	0.4000	0.2857	−0.7799	0.7823
SE0616	SERP0510	2.330294	939.635	377.365	939.935	377.065	940.873	376.127	2	2	0.0021	0.0053	0.0021	0.0053	0.3962	0.2838	−0.7833	0.7833
SE0226	SERP2354	2.330294	546.508	218.492	546.808	218.192	547.746	217.254	2	2	0.0037	0.0092	0.0037	0.0092	0.4022	0.2868	−0.7856	0.7840
SE1427	SERP1313	2.330294	1175.731	483.269	1176.181	482.819	1177.044	481.956	2	2	0.0017	0.0041	0.0017	0.0042	0.4048	0.2881	−0.7966	0.7871
SE2087	SERP2101	2.330294	287.898	114.102	287.022	114.978	288.548	113.452	2	2	0.0070	0.0175	0.0070	0.0177	0.3955	0.2834	−0.7970	0.7873
SE1434	SERP1321	2.330294	403.434	157.566	403.611	157.389	404.611	156.389	2	2	0.0050	0.0127	0.0050	0.0128	0.3906	0.2809	−0.8000	0.7881
SE1439	SERP1326	2.330294	851.801	327.199	851.978	327.022	852.978	326.022	2	2	0.0023	0.0061	0.0024	0.0061	0.3934	0.2824	−0.8002	0.7882
SE2373	SERP0043	2.330294	580.209	226.791	580.209	226.791	581.298	225.702	2	2	0.0034	0.0088	0.0035	0.0089	0.3933	0.2823	−0.8010	0.7884
SE0679	SERP0569	2.330294	265.990	103.010	265.690	103.310	266.928	102.072	2	2	0.0075	0.0194	0.0076	0.0196	0.3878	0.2794	−0.8016	0.7886
SE1461	SERP1355	2.330294	340.749	133.251	340.625	133.375	341.775	132.225	2	2	0.0059	0.0150	0.0059	0.0152	0.3882	0.2796	−0.8091	0.7908
SE0921	SERP0811	2.330294	622.310	238.690	622.460	238.540	623.473	237.527	2	2	0.0032	0.0084	0.0032	0.0084	0.3810	0.2759	−0.8092	0.7908
SE1987	SERP1999	2.330294	431.713	162.287	431.112	162.888	432.501	161.499	2	2	0.0046	0.0123	0.0047	0.0124	0.3790	0.2749	−0.8193	0.7937
SE0154	SERP2414	2.330294	260.569	96.431	260.268	96.732	261.507	95.493	2	2	0.0077	0.0207	0.0077	0.0210	0.3667	0.2683	−0.8392	0.7993
SE1938	SERP1950	2.330294	679.493	274.507	679.943	274.057	680.806	273.194	5	4	0.0074	0.0146	0.0074	0.0147	0.5034	0.3348	−0.9010	0.8162
SE2177	SERP2188	2.330294	431.631	177.369	430.932	178.068	432.370	176.630	3	3	0.0070	0.0169	0.0070	0.0171	0.4094	0.2905	−0.9459	0.8279
SE1550	SERP1403	2.330294	1227.671	506.329	1227.097	506.903	1228.472	505.528	3	3	0.0024	0.0059	0.0024	0.0059	0.4068	0.2892	−0.9519	0.8294
SE0818	SERP0709	2.330294	307.775	124.225	308.625	123.375	309.288	122.712	3	3	0.0097	0.0242	0.0098	0.0246	0.3984	0.2849	−0.9579	0.8309
SE1274	SERP1155	2.330294	1575.316	638.684	1577.715	636.285	1577.604	636.396	3	3	0.0019	0.0047	0.0019	0.0047	0.4043	0.2879	−0.9604	0.8316
SE1345	SERP1234	2.330294	656.573	267.427	656.573	267.427	657.661	266.339	0	1	0.0000	0.0037	0.0000	0.0037	0.0000	0.0000	−0.9737	0.8349
SE0715	SERP0604	2.330294	363.151	143.849	363.701	143.299	364.514	142.486	3	3	0.0083	0.0209	0.0083	0.0212	0.3915	0.2814	−0.9770	0.8357
SE0205	SERP2372	2.330294	1140.231	455.769	1140.231	455.769	1141.319	454.681	3	3	0.0026	0.0066	0.0026	0.0066	0.3939	0.2826	−0.9791	0.8362
SE0620	SERP0514	2.330294	310.422	121.578	309.422	122.578	309.922	122.078	3	3	0.0097	0.0246	0.0097	0.0250	0.3880	0.2795	−0.9843	0.8375
SE2092	SERP2106	2.330294	316.821	133.179	316.821	133.179	317.909	132.091	0	1	0.0000	0.0075	0.0000	0.0075	0.0000	0.0000	−0.9868	0.8381
SE1790	SERP1798	2.330294	269.442	120.558	269.442	120.558	270.530	119.470	0	1	0.0000	0.0083	0.0000	0.0083	0.0000	0.0000	−0.9881	0.8384
SE2418	SERP0002	2.330294	250.182	94.818	250.182	94.818	251.270	93.730	0	1	0.0000	0.0105	0.0000	0.0106	0.0000	0.0000	−0.9907	0.8391
SE1812	SERP1820	2.330294	224.815	90.185	225.365	89.635	226.178	88.822	0	1	0.0000	0.0111	0.0000	0.0112	0.0000	0.0000	−0.9912	0.8392
SE1450	SERP1337	2.330294	221.188	84.812	220.639	85.361	222.002	83.998	0	1	0.0000	0.0118	0.0000	0.0118	0.0000	0.0000	−0.9916	0.8393
SE1882	SERP1892	2.330294	193.212	79.788	193.212	79.788	194.300	78.700	0	1	0.0000	0.0125	0.0000	0.0126	0.0000	0.0000	−0.9921	0.8394
SE0626	SERP0520	2.330294	167.563	66.437	167.563	66.437	168.651	65.349	0	1	0.0000	0.0151	0.0000	0.0152	0.0000	0.0000	−0.9935	0.8398
SE1775	SERP1783	2.330294	171.263	65.737	171.263	65.737	172.351	64.649	0	1	0.0000	0.0152	0.0000	0.0154	0.0000	0.0000	−0.9935	0.8398
SE1024	SERP0911	2.330294	171.734	62.266	171.734	62.266	172.822	61.178	0	1	0.0000	0.0161	0.0000	0.0162	0.0000	0.0000	−0.9939	0.8399
SE0230	SERP2350	2.330294	160.338	61.662	160.338	61.662	161.427	60.573	0	1	0.0000	0.0162	0.0000	0.0164	0.0000	0.0000	−0.9939	0.8399
SE0783	SERP0671	2.330294	172.866	61.134	172.866	61.134	173.954	60.046	0	1	0.0000	0.0164	0.0000	0.0165	0.0000	0.0000	−0.9940	0.8399
SE1742	SERP1751	2.330294	164.580	60.420	164.580	60.420	165.668	59.332	0	1	0.0000	0.0166	0.0000	0.0167	0.0000	0.0000	−0.9940	0.8399
SE1003	SERP0890	2.330294	131.258	54.742	131.258	54.742	132.346	53.654	0	1	0.0000	0.0183	0.0000	0.0185	0.0000	0.0000	−0.9946	0.8400
SE0297	SERP0175	2.330294	99.710	41.290	99.710	41.290	100.798	40.202	0	1	0.0000	0.0242	0.0000	0.0246	0.0000	0.0000	−0.9960	0.8404
SE2359	SERP0058	2.330294	97.054	28.946	97.054	28.946	98.142	27.858	0	1	0.0000	0.0345	0.0000	0.0354	0.0000	0.0000	−0.9972	0.8407
SE2411	SERP0010	2.330294	284.290	108.710	284.290	108.710	285.378	107.622	0	1	0.0000	0.0092	0.0000	0.0093	0.0000	0.0000	−1.0000	0.8413
SE2406	SERP0015	2.330294	487.736	196.264	488.436	195.564	489.174	194.826	0	1	0.0000	0.0051	0.0000	0.0051	0.0000	0.0000	−1.0000	0.8413

SE2405	SERP0016	2.330294	855.377	338.623	855.377	338.623	856.466	337.534	0	1	0.0000	0.0030	0.0000	0.0030	0.0000	0.0000	−1.0000	0.8413
SE2401	SERP0020	2.330294	1351.233	526.767	1351.233	526.767	1352.321	525.679	0	1	0.0000	0.0019	0.0000	0.0019	0.0000	0.0000	−1.0000	0.8413
SE2371	SERP0045	2.330294	358.424	148.576	358.924	148.076	359.762	147.238	0	1	0.0000	0.0067	0.0000	0.0068	0.0000	0.0000	−1.0000	0.8413
SE2349	SERP0068	2.330294	889.951	376.049	889.951	376.049	891.040	374.960	0	1	0.0000	0.0027	0.0000	0.0027	0.0000	0.0000	−1.0000	0.8413
SE2341	SERP0074	2.330294	446.982	168.018	446.982	168.018	448.070	166.930	0	1	0.0000	0.0060	0.0000	0.0060	0.0000	0.0000	−1.0000	0.8413
SE2323	SERP0095	2.330294	805.470	337.530	805.470	337.530	806.559	336.441	0	1	0.0000	0.0030	0.0000	0.0030	0.0000	0.0000	−1.0000	0.8413
SE2322	SERP0097	2.330294	733.079	289.921	733.379	289.621	734.318	288.682	0	1	0.0000	0.0035	0.0000	0.0035	0.0000	0.0000	−1.0000	0.8413
SE2306	SERP0115	2.330294	232.266	82.734	232.266	82.734	233.354	81.646	0	1	0.0000	0.0121	0.0000	0.0122	0.0000	0.0000	−1.0000	0.8413
SE2298	SERP0123	2.330294	568.549	232.451	568.549	232.451	569.638	231.362	0	1	0.0000	0.0043	0.0000	0.0043	0.0000	0.0000	−1.0000	0.8413
SE2271	SERP0151	2.330294	626.538	252.462	626.538	252.462	627.627	251.373	0	1	0.0000	0.0040	0.0000	0.0040	0.0000	0.0000	−1.0000	0.8413
SE0307	SERP0184	2.330294	2565.305	1055.695	2565.305	1055.695	2566.394	1054.606	0	1	0.0000	0.0009	0.0000	0.0009	0.0000	0.0000	−1.0000	0.8413
SE0421	SERP0306	2.330294	561.167	233.833	561.167	233.833	562.255	232.745	0	1	0.0000	0.0043	0.0000	0.0043	0.0000	0.0000	−1.0000	0.8413
SE0503	SERP0386	2.330294	1073.556	438.444	1073.556	438.444	1074.644	437.356	0	1	0.0000	0.0023	0.0000	0.0023	0.0000	0.0000	−1.0000	0.8413
SE0515	SERP0400	2.330294	679.743	292.257	679.443	292.557	680.681	291.319	0	1	0.0000	0.0034	0.0000	0.0034	0.0000	0.0000	−1.0000	0.8413
SE0525	SERP0410	2.330294	880.985	355.015	880.985	355.015	882.074	353.926	0	1	0.0000	0.0028	0.0000	0.0028	0.0000	0.0000	−1.0000	0.8413
SE0550	SERP0435	2.330294	676.890	265.110	676.890	265.110	677.978	264.022	0	1	0.0000	0.0038	0.0000	0.0038	0.0000	0.0000	−1.0000	0.8413
SE0564	SERP0449	2.330294	529.610	208.390	529.610	208.390	530.698	207.302	0	1	0.0000	0.0048	0.0000	0.0048	0.0000	0.0000	−1.0000	0.8413
SE0571	SERP0458	2.330294	919.581	385.419	919.581	385.419	920.669	384.331	0	1	0.0000	0.0026	0.0000	0.0026	0.0000	0.0000	−1.0000	0.8413
SE0598	SERP0487	2.330294	279.770	104.230	279.770	104.230	280.858	103.142	0	1	0.0000	0.0096	0.0000	0.0097	0.0000	0.0000	−1.0000	0.8413
SE0646	SERP0538	2.330294	1686.313	716.687	1686.313	716.687	1687.401	715.599	0	1	0.0000	0.0014	0.0000	0.0014	0.0000	0.0000	−1.0000	0.8413
SE0657	SERP0549	2.330294	852.458	350.542	852.458	350.542	853.546	349.454	0	1	0.0000	0.0029	0.0000	0.0029	0.0000	0.0000	−1.0000	0.8413
SE0673	SERP0563	2.330294	1292.278	528.722	1291.579	529.421	1293.017	527.983	0	1	0.0000	0.0019	0.0000	0.0019	0.0000	0.0000	−1.0000	0.8413
SE0677	SERP0567	2.330294	677.177	261.823	677.177	261.823	678.266	260.734	0	1	0.0000	0.0038	0.0000	0.0038	0.0000	0.0000	−1.0000	0.8413
SE0712	SERP0601	2.330294	539.112	228.888	539.112	228.888	540.201	227.799	0	1	0.0000	0.0044	0.0000	0.0044	0.0000	0.0000	−1.0000	0.8413
SE0714	SERP0603	2.330294	540.038	212.962	540.038	212.962	541.126	211.874	0	1	0.0000	0.0047	0.0000	0.0047	0.0000	0.0000	−1.0000	0.8413
SE0728	SERP0616	2.330294	718.670	265.330	718.670	265.330	719.758	264.242	0	1	0.0000	0.0038	0.0000	0.0038	0.0000	0.0000	−1.0000	0.8413
SE0774	SERP0661	2.330294	994.552	406.448	994.552	406.448	995.640	405.360	0	1	0.0000	0.0025	0.0000	0.0025	0.0000	0.0000	−1.0000	0.8413
SE0789	SERP0678	2.330294	392.800	156.200	392.800	156.200	393.888	155.112	0	1	0.0000	0.0064	0.0000	0.0064	0.0000	0.0000	−1.0000	0.8413
SE0791	SERP0680	2.330294	789.493	320.507	789.493	320.507	790.582	319.418	0	1	0.0000	0.0031	0.0000	0.0031	0.0000	0.0000	−1.0000	0.8413
SE0899	SERP0790	2.330294	132.904	53.096	132.904	53.096	133.992	52.008	0	1	0.0000	0.0188	0.0000	0.0191	0.0000	0.0000	−1.0000	0.8413
SE0913	SERP0804	2.330294	192.012	80.988	192.012	80.988	193.100	79.900	0	1	0.0000	0.0123	0.0000	0.0125	0.0000	0.0000	−1.0000	0.8413
SE0923	SERP0813	2.330294	819.010	344.990	819.010	344.990	820.098	343.902	0	1	0.0000	0.0029	0.0000	0.0029	0.0000	0.0000	−1.0000	0.8413
SE1016	SERP0903	2.330294	1088.590	423.410	1088.590	423.410	1089.678	422.322	0	1	0.0000	0.0024	0.0000	0.0024	0.0000	0.0000	−1.0000	0.8413
SE1036	SERP0925	2.330294	1421.639	576.361	1421.639	576.361	1422.727	575.273	0	1	0.0000	0.0017	0.0000	0.0017	0.0000	0.0000	−1.0000	0.8413
SE1046	SERP0935	2.330294	900.131	359.869	900.131	359.869	901.220	358.780	0	1	0.0000	0.0028	0.0000	0.0028	0.0000	0.0000	−1.0000	0.8413
SE1049	SERP0938	2.330294	402.983	161.017	402.983	161.017	404.072	159.928	0	1	0.0000	0.0062	0.0000	0.0062	0.0000	0.0000	−1.0000	0.8413
SE1052	SERP0941	2.330294	453.002	167.998	453.551	167.449	454.364	166.636	0	1	0.0000	0.0060	0.0000	0.0060	0.0000	0.0000	−1.0000	0.8413
SE1054	SERP0943	2.330294	523.932	205.068	523.932	205.068	525.020	203.980	0	1	0.0000	0.0049	0.0000	0.0049	0.0000	0.0000	−1.0000	0.8413
SE1123	SERP1006	2.330294	316.397	118.603	316.397	118.603	317.485	117.515	0	1	0.0000	0.0084	0.0000	0.0085	0.0000	0.0000	−1.0000	0.8413

SE1138	SERP1020	2.330294	1593.075	635.925	1593.075	635.925	1594.164	634.836	0	1	0.0000	0.0016	0.0000	0.0016	0.0000	0.0000	-1.0000	0.8413
SE1144	SERP1026	2.330294	703.466	265.534	703.466	265.534	704.554	264.446	0	1	0.0000	0.0038	0.0000	0.0038	0.0000	0.0000	-1.0000	0.8413
SE1159	SERP1040	2.330294	410.856	165.144	410.856	165.144	411.944	164.056	0	1	0.0000	0.0061	0.0000	0.0061	0.0000	0.0000	-1.0000	0.8413
SE1180	SERP1059	2.330294	374.129	147.871	374.129	147.871	375.217	146.783	0	1	0.0000	0.0068	0.0000	0.0068	0.0000	0.0000	-1.0000	0.8413
SE1182	SERP1061	2.330294	316.678	130.322	316.678	130.322	317.766	129.234	0	1	0.0000	0.0077	0.0000	0.0077	0.0000	0.0000	-1.0000	0.8413
SE1184	SERP1063	2.330294	643.950	262.050	643.950	262.050	645.038	260.962	0	1	0.0000	0.0038	0.0000	0.0038	0.0000	0.0000	-1.0000	0.8413
SE1185	SERP1064	2.330294	543.057	212.943	543.057	212.943	544.145	211.855	0	1	0.0000	0.0047	0.0000	0.0047	0.0000	0.0000	-1.0000	0.8413
SE1215	SERP1095	2.330294	418.207	157.793	417.658	158.342	419.021	156.979	0	1	0.0000	0.0063	0.0000	0.0064	0.0000	0.0000	-1.0000	0.8413
SE1227	SERP1106	2.330294	321.624	125.376	321.624	125.376	322.713	124.287	0	1	0.0000	0.0080	0.0000	0.0080	0.0000	0.0000	-1.0000	0.8413
SE1231	SERP1110	2.330294	443.584	177.416	443.584	177.416	444.672	176.328	0	1	0.0000	0.0056	0.0000	0.0057	0.0000	0.0000	-1.0000	0.8413
SE1252	SERP1132	2.330294	1009.445	379.555	1009.445	379.555	1010.534	378.466	0	1	0.0000	0.0026	0.0000	0.0026	0.0000	0.0000	-1.0000	0.8413
SE1253	SERP1133	2.330294	543.439	215.561	543.439	215.561	544.527	214.473	0	1	0.0000	0.0046	0.0000	0.0047	0.0000	0.0000	-1.0000	0.8413
SE1255	SERP1135	2.330294	289.273	112.727	289.273	112.727	290.361	111.639	0	1	0.0000	0.0089	0.0000	0.0089	0.0000	0.0000	-1.0000	0.8413
SE1264	SERP1145	2.330294	551.665	207.335	551.665	207.335	552.754	206.246	0	1	0.0000	0.0048	0.0000	0.0048	0.0000	0.0000	-1.0000	0.8413
SE1269	SERP1150	2.330294	700.022	274.978	699.322	275.678	700.760	274.240	0	1	0.0000	0.0036	0.0000	0.0036	0.0000	0.0000	-1.0000	0.8413
SE1299	SERP1180	2.330294	307.441	118.559	307.441	118.559	308.529	117.471	0	1	0.0000	0.0084	0.0000	0.0085	0.0000	0.0000	-1.0000	0.8413
SE1315	SERP1196	2.330294	1574.316	612.684	1574.316	612.684	1575.405	611.595	0	1	0.0000	0.0016	0.0000	0.0016	0.0000	0.0000	-1.0000	0.8413
SE1318	SERP1199	2.330294	1632.863	638.137	1632.863	638.137	1633.951	637.049	0	1	0.0000	0.0016	0.0000	0.0016	0.0000	0.0000	-1.0000	0.8413
SE1350	SERP1239	2.330294	932.694	366.306	932.694	366.306	933.782	365.218	0	1	0.0000	0.0027	0.0000	0.0027	0.0000	0.0000	-1.0000	0.8413
SE1355	SERP1244	2.330294	379.679	145.321	379.679	145.321	380.767	144.233	0	1	0.0000	0.0069	0.0000	0.0069	0.0000	0.0000	-1.0000	0.8413
SE1381	SERP1269	2.330294	286.919	124.081	286.919	124.081	288.007	122.993	0	1	0.0000	0.0081	0.0000	0.0081	0.0000	0.0000	-1.0000	0.8413
SE1387	SERP1275	2.330294	892.048	358.952	892.048	358.952	893.137	357.863	0	1	0.0000	0.0028	0.0000	0.0028	0.0000	0.0000	-1.0000	0.8413
SE1391	SERP1279	2.330294	879.546	341.454	879.546	341.454	880.634	340.366	0	1	0.0000	0.0029	0.0000	0.0029	0.0000	0.0000	-1.0000	0.8413
SE1404	SERP1291	2.330294	442.607	178.393	442.607	178.393	443.695	177.305	0	1	0.0000	0.0056	0.0000	0.0056	0.0000	0.0000	-1.0000	0.8413
SE1436	SERP1323	2.330294	597.482	227.518	597.482	227.518	598.571	226.429	0	1	0.0000	0.0044	0.0000	0.0044	0.0000	0.0000	-1.0000	0.8413
SE1443	SERP1330	2.330294	616.015	232.985	616.315	232.685	617.254	231.746	0	1	0.0000	0.0043	0.0000	0.0043	0.0000	0.0000	-1.0000	0.8413
SE1451	SERP1338	2.330294	252.515	110.485	252.515	110.485	253.603	109.397	0	1	0.0000	0.0091	0.0000	0.0091	0.0000	0.0000	-1.0000	0.8413
SE1518	SERP1373	2.330294	309.692	113.308	309.692	113.308	310.781	112.219	0	1	0.0000	0.0088	0.0000	0.0089	0.0000	0.0000	-1.0000	0.8413
SE1524	SERP1379	2.330294	2110.506	826.494	2110.506	826.494	2111.594	825.406	0	1	0.0000	0.0012	0.0000	0.0012	0.0000	0.0000	-1.0000	0.8413
SE1529	SERP1384	2.330294	444.004	176.996	444.004	176.996	445.092	175.908	0	1	0.0000	0.0056	0.0000	0.0057	0.0000	0.0000	-1.0000	0.8413
SE1554	SERP1408	2.330294	594.963	233.037	594.963	233.037	596.051	231.949	0	1	0.0000	0.0043	0.0000	0.0043	0.0000	0.0000	-1.0000	0.8413
SE1566	SERP1419	2.330294	336.947	125.053	336.947	125.053	338.035	123.965	0	1	0.0000	0.0080	0.0000	0.0080	0.0000	0.0000	-1.0000	0.8413
SE1569	SERP1422	2.330294	451.426	175.574	451.426	175.574	452.514	174.486	0	1	0.0000	0.0057	0.0000	0.0057	0.0000	0.0000	-1.0000	0.8413
SE1572	SERP1425	2.330294	284.343	108.657	284.343	108.657	285.431	107.569	0	1	0.0000	0.0092	0.0000	0.0093	0.0000	0.0000	-1.0000	0.8413
SE1586	SERP1439	2.330294	214.792	85.208	214.792	85.208	215.880	84.120	0	1	0.0000	0.0117	0.0000	0.0118	0.0000	0.0000	-1.0000	0.8413
SE1593	SERP1446	2.330294	924.474	368.526	924.474	368.526	925.563	367.437	0	1	0.0000	0.0027	0.0000	0.0027	0.0000	0.0000	-1.0000	0.8413
SE1606	SERP1459	2.330294	278.268	99.732	277.768	100.232	279.106	98.894	0	1	0.0000	0.0100	0.0000	0.0101	0.0000	0.0000	-1.0000	0.8413
SE1637	SERP1492	2.330294	924.959	362.041	925.659	361.341	926.397	360.603	0	1	0.0000	0.0028	0.0000	0.0028	0.0000	0.0000	-1.0000	0.8413
SE1654	SERP1665	2.330294	1197.057	488.943	1196.507	489.493	1197.870	488.130	0	1	0.0000	0.0020	0.0000	0.0020	0.0000	0.0000	-1.0000	0.8413

SE1698	SERP1706	2.330294	892.072	370.928	891.522	371.478	892.885	370.115	0	1	0.0000	0.0027	0.0000	0.0027	0.0000	0.0000	−1.0000	0.8413
SE1701	SERP1710	2.330294	618.732	245.268	618.732	245.268	619.821	244.179	0	1	0.0000	0.0041	0.0000	0.0041	0.0000	0.0000	−1.0000	0.8413
SE1729	SERP1738	2.330294	482.581	180.419	482.581	180.419	483.669	179.331	0	1	0.0000	0.0055	0.0000	0.0056	0.0000	0.0000	−1.0000	0.8413
SE1739	SERP1749	2.330294	292.671	115.329	292.671	115.329	293.759	114.241	0	1	0.0000	0.0087	0.0000	0.0087	0.0000	0.0000	−1.0000	0.8413
SE1781	SERP1789	2.330294	1018.138	391.862	1018.138	391.862	1019.226	390.774	0	1	0.0000	0.0026	0.0000	0.0026	0.0000	0.0000	−1.0000	0.8413
SE1802	SERP1810	2.330294	462.057	182.943	462.057	182.943	463.145	181.855	0	1	0.0000	0.0055	0.0000	0.0055	0.0000	0.0000	−1.0000	0.8413
SE1811	SERP1819	2.330294	380.524	156.476	380.524	156.476	381.612	155.388	0	1	0.0000	0.0064	0.0000	0.0064	0.0000	0.0000	−1.0000	0.8413
SE1823	SERP1830	2.330294	436.524	184.476	435.824	185.176	437.262	183.738	0	1	0.0000	0.0054	0.0000	0.0054	0.0000	0.0000	−1.0000	0.8413
SE1834	SERP1843	2.330294	2249.922	903.078	2249.922	903.078	2251.010	901.990	0	1	0.0000	0.0011	0.0000	0.0011	0.0000	0.0000	−1.0000	0.8413
SE1890	SERP1900	2.330294	999.059	422.941	999.059	422.941	1000.147	421.853	0	1	0.0000	0.0024	0.0000	0.0024	0.0000	0.0000	−1.0000	0.8413
SE1935	SERP1947	2.330294	850.345	358.655	850.372	358.628	851.447	357.553	0	1	0.0000	0.0028	0.0000	0.0028	0.0000	0.0000	−1.0000	0.8413
SE1952	SERP1964	2.330294	291.044	122.956	291.744	122.256	292.482	121.518	0	1	0.0000	0.0082	0.0000	0.0082	0.0000	0.0000	−1.0000	0.8413
SE1960	SERP1969	2.330294	266.372	108.628	266.372	108.628	267.460	107.540	0	1	0.0000	0.0092	0.0000	0.0093	0.0000	0.0000	−1.0000	0.8413
SE1967	SERP1979	2.330294	313.227	130.773	313.227	130.773	314.315	129.685	0	1	0.0000	0.0076	0.0000	0.0077	0.0000	0.0000	−1.0000	0.8413
SE1977	SERP1989	2.330294	224.420	87.580	224.420	87.580	225.508	86.492	0	1	0.0000	0.0114	0.0000	0.0115	0.0000	0.0000	−1.0000	0.8413
SE1991	SERP2003	2.330294	523.233	205.767	523.233	205.767	524.321	204.679	0	1	0.0000	0.0049	0.0000	0.0049	0.0000	0.0000	−1.0000	0.8413
SE2018	SERP2031	2.330294	895.513	358.487	895.513	358.487	896.601	357.399	0	1	0.0000	0.0028	0.0000	0.0028	0.0000	0.0000	−1.0000	0.8413
SE2024	SERP2037	2.330294	542.580	231.420	542.580	231.420	543.669	230.331	0	1	0.0000	0.0043	0.0000	0.0043	0.0000	0.0000	−1.0000	0.8413
SE2029	SERP2042	2.330294	337.750	127.250	337.750	127.250	338.838	126.162	0	1	0.0000	0.0079	0.0000	0.0079	0.0000	0.0000	−1.0000	0.8413
SE2071	SERP2084	2.330294	1055.273	435.727	1055.273	435.727	1056.361	434.639	0	1	0.0000	0.0023	0.0000	0.0023	0.0000	0.0000	−1.0000	0.8413
SE2102	SERP2114	2.330294	1431.092	593.908	1431.092	593.908	1432.181	592.819	0	1	0.0000	0.0017	0.0000	0.0017	0.0000	0.0000	−1.0000	0.8413
SE2106	SERP2118	2.330294	636.548	245.452	636.548	245.452	637.636	244.364	0	1	0.0000	0.0041	0.0000	0.0041	0.0000	0.0000	−1.0000	0.8413
SE2116	SERP2128	2.330294	1098.969	443.031	1098.969	443.031	1100.057	441.943	0	1	0.0000	0.0023	0.0000	0.0023	0.0000	0.0000	−1.0000	0.8413
SE2119	SERP2131	2.330294	1689.040	692.960	1689.040	692.960	1690.128	691.872	0	1	0.0000	0.0014	0.0000	0.0014	0.0000	0.0000	−1.0000	0.8413
SE2145	SERP2156	2.330294	666.291	281.709	666.591	281.409	667.529	280.471	0	1	0.0000	0.0036	0.0000	0.0036	0.0000	0.0000	−1.0000	0.8413
SE2210	SERP2242	2.330294	1174.949	472.051	1175.498	471.502	1176.312	470.688	0	1	0.0000	0.0021	0.0000	0.0021	0.0000	0.0000	−1.0000	0.8413
SE2212	SERP2244	2.330294	841.258	322.742	841.258	322.742	842.346	321.654	0	1	0.0000	0.0031	0.0000	0.0031	0.0000	0.0000	−1.0000	0.8413
SE2238	SERP2270	2.330294	825.006	344.994	825.006	344.994	826.094	343.906	0	1	0.0000	0.0029	0.0000	0.0029	0.0000	0.0000	−1.0000	0.8413
SE0264	SERP2314	2.330294	372.677	140.323	372.677	140.323	373.765	139.235	0	1	0.0000	0.0071	0.0000	0.0072	0.0000	0.0000	−1.0000	0.8413
SE0255	SERP2325	2.330294	733.296	304.704	733.296	304.704	734.384	303.616	0	1	0.0000	0.0033	0.0000	0.0033	0.0000	0.0000	−1.0000	0.8413
SE0160	SERP2411	2.330294	545.102	219.898	545.102	219.898	546.190	218.810	0	1	0.0000	0.0045	0.0000	0.0046	0.0000	0.0000	−1.0000	0.8413
SE0623	SERP0517	2.330294	102.308	41.692	102.308	41.692	103.397	40.603	0	1	0.0000	0.0240	0.0000	0.0244	0.0000	0.0000	−1.0167	0.8453
SE0412	SERP0297	2.330294	579.307	230.693	579.607	230.393	580.545	229.455	0	1	0.0000	0.0043	0.0000	0.0044	0.0000	0.0000	−1.0233	0.8469
SE0188	SERP2386	2.330294	624.553	254.447	624.853	254.147	624.703	254.297	4	4	0.0064	0.0157	0.0064	0.0159	0.4025	0.2870	−1.0674	0.8571
SE0258	SERP2321	2.330294	361.848	148.152	361.998	148.002	363.011	146.989	1	2	0.0028	0.0135	0.0028	0.0136	0.2059	0.1707	−1.0697	0.8576
SE1015	SERP0902	2.330294	1023.723	422.277	1023.900	422.100	1024.900	421.100	1	2	0.0010	0.0047	0.0010	0.0048	0.2083	0.1724	−1.0722	0.8582
SE0870	SERP0761	2.330294	345.845	122.155	345.145	122.855	345.495	122.505	11	7	0.0318	0.0571	0.0325	0.0594	0.5471	0.3536	−1.0737	0.8585
SE0615	SERP0509	2.330294	578.601	246.399	579.300	245.700	580.039	244.961	1	2	0.0017	0.0081	0.0017	0.0082	0.2073	0.1717	−1.0754	0.8589
SE0210	SERP2368	2.330294	530.396	216.604	529.996	217.004	531.284	215.716	1	2	0.0019	0.0092	0.0019	0.0093	0.2043	0.1696	−1.0775	0.8594

SE2114	SERP2126	2.330294	1417.387	574.613	1417.387	574.613	1418.475	573.525	1	2	0.0007	0.0035	0.0007	0.0035	0.2000	0.1667	-1.0785	0.8596
SE0667	SERP0558	2.330294	725.225	285.775	725.198	285.802	726.299	284.701	1	2	0.0014	0.0070	0.0014	0.0070	0.2000	0.1667	-1.0785	0.8596
SE1941	SERP1953	2.330294	478.833	193.167	478.533	193.467	479.771	192.229	1	2	0.0021	0.0103	0.0021	0.0104	0.2019	0.1680	-1.0790	0.8597
SE1460	SERP1354	2.330294	562.397	220.603	562.397	220.603	563.486	219.514	1	2	0.0018	0.0091	0.0018	0.0091	0.1978	0.1651	-1.0823	0.8604
SE1457	SERP1351	2.330294	646.873	259.127	646.174	259.826	647.612	258.388	1	2	0.0015	0.0077	0.0015	0.0077	0.1948	0.1630	-1.0876	0.8616
SE0270	SERP2307	2.330294	583.137	226.863	581.787	228.213	583.550	226.450	6	5	0.0103	0.0220	0.0104	0.0223	0.4664	0.3180	-1.0879	0.8617
SE1512	SERP1367	2.330294	657.896	263.104	658.047	262.953	659.060	261.940	1	2	0.0015	0.0076	0.0015	0.0076	0.1974	0.1648	-1.0884	0.8618
SE2132	SERP2144	2.330294	749.261	312.739	749.261	312.739	750.349	311.651	1	2	0.0013	0.0064	0.0013	0.0064	0.2031	0.1688	-1.0888	0.8619
SE2112	SERP2124	2.330294	328.400	133.600	328.674	133.326	329.625	132.375	1	2	0.0030	0.0150	0.0030	0.0151	0.1987	0.1657	-1.0889	0.8619
SE1876	SERP1884	2.330294	336.444	134.556	336.744	134.256	337.682	133.318	1	2	0.0030	0.0149	0.0030	0.0150	0.2000	0.1667	-1.0893	0.8620
SE1791	SERP1799	2.330294	308.245	126.755	308.545	126.455	309.483	125.517	1	2	0.0032	0.0158	0.0032	0.0160	0.2000	0.1667	-1.0899	0.8621
SE1517	SERP1372	2.330294	526.958	211.042	526.334	211.666	527.734	210.266	1	2	0.0019	0.0095	0.0019	0.0095	0.2000	0.1667	-1.0913	0.8624
SE1961	SERP1972	2.330294	1501.552	601.448	1502.651	600.349	1503.190	599.810	6	5	0.0040	0.0083	0.0040	0.0084	0.4762	0.3226	-1.0915	0.8625
SE1440	SERP1327	2.330294	453.048	176.952	452.898	177.102	454.061	175.939	1	2	0.0022	0.0113	0.0022	0.0114	0.1930	0.1618	-1.0961	0.8635
SE0026	SERP2470	2.330294	355.652	139.348	355.652	139.348	356.741	138.259	1	2	0.0028	0.0144	0.0028	0.0145	0.1931	0.1618	-1.0961	0.8635
SE2004	SERP2017	2.330294	970.627	403.373	970.777	403.223	971.791	402.209	1	2	0.0010	0.0050	0.0010	0.0050	0.2000	0.1667	-1.0989	0.8641
SE0826	SERP0717	2.330294	399.277	152.723	398.977	153.023	400.215	151.785	1	2	0.0025	0.0131	0.0025	0.0132	0.1894	0.1592	-1.1001	0.8643
SE1968	SERP1980	2.330294	818.802	342.198	818.802	342.198	819.890	341.110	1	2	0.0012	0.0058	0.0012	0.0059	0.2034	0.1690	-1.1002	0.8644
SE1433	SERP1320	2.330294	689.372	261.628	689.372	261.628	690.460	260.540	1	2	0.0015	0.0076	0.0015	0.0077	0.1948	0.1630	-1.1063	0.8657
SE0221	SERP2359	2.330294	354.226	131.774	354.226	131.774	355.314	130.686	1	2	0.0028	0.0152	0.0028	0.0153	0.1830	0.1547	-1.1107	0.8667
SE1648	SERP1658	2.330294	1396.397	535.603	1396.697	535.303	1397.635	534.365	1	2	0.0007	0.0037	0.0007	0.0037	0.1892	0.1591	-1.1142	0.8674
SE1951	SERP1963	2.330294	290.543	108.457	290.543	108.457	291.631	107.369	1	2	0.0034	0.0184	0.0034	0.0187	0.1818	0.1538	-1.1225	0.8692
SE0152	SERP2418	2.330294	295.872	112.128	295.545	112.455	295.708	112.292	10	7	0.0338	0.0623	0.0346	0.0651	0.5315	0.3470	-1.1276	0.8702
SE1368	SERP1255	2.330294	1212.323	482.677	1212.623	482.377	1213.561	481.439	1	2	0.0008	0.0041	0.0008	0.0042	0.1905	0.1600	-1.1302	0.8708
SE2213	SERP2245	2.330294	543.457	212.543	543.457	212.543	544.546	211.454	4	4	0.0074	0.0188	0.0074	0.0191	0.3874	0.2792	-1.1476	0.8744
SE1002	SERP0889	2.330294	433.323	166.677	434.023	165.977	434.762	165.238	4	4	0.0092	0.0240	0.0093	0.0244	0.3811	0.2760	-1.1499	0.8749
SE0886	SERP0777	2.330294	146.757	63.243	146.757	63.243	147.845	62.155	2	3	0.0136	0.0474	0.0138	0.0490	0.2816	0.2197	-1.1583	0.8766
SE2019	SERP2032	2.330294	736.547	313.453	737.246	312.754	737.985	312.015	2	3	0.0027	0.0096	0.0027	0.0096	0.2813	0.2195	-1.1668	0.8784
SE1516	SERP1371	2.330294	862.977	358.023	863.377	357.623	864.265	356.735	2	3	0.0023	0.0084	0.0023	0.0084	0.2738	0.2150	-1.1834	0.8817
SE0785	SERP0673	2.330294	715.919	298.081	717.069	296.931	717.582	296.418	2	3	0.0028	0.0101	0.0028	0.0102	0.2745	0.2154	-1.1878	0.8826
SE0456	SERP0341	2.330294	1205.250	486.750	1204.700	487.300	1206.063	485.937	7	6	0.0058	0.0123	0.0058	0.0124	0.4677	0.3187	-1.1883	0.8826
SE0814	SERP0705	2.330294	640.360	265.640	639.360	266.640	640.948	265.052	2	3	0.0031	0.0113	0.0031	0.0114	0.2719	0.2138	-1.1930	0.8836
SE0263	SERP2315	2.330294	692.874	282.126	692.874	282.126	693.962	281.038	2	3	0.0029	0.0106	0.0029	0.0107	0.2710	0.2132	-1.1973	0.8844
SE1375	SERP1263	2.330294	672.912	269.088	672.612	269.388	673.850	268.150	2	3	0.0030	0.0111	0.0030	0.0112	0.2679	0.2113	-1.2004	0.8850
SE2156	SERP2166	2.330294	632.202	255.798	632.002	255.998	633.190	254.810	2	3	0.0032	0.0117	0.0032	0.0118	0.2712	0.2133	-1.2033	0.8856
SE0581	SERP0469	2.330294	530.835	213.165	530.835	213.165	531.923	212.077	2	3	0.0038	0.0141	0.0038	0.0142	0.2676	0.2111	-1.2047	0.8858
SE2077	SERP2090	2.330294	349.543	142.457	349.843	142.157	350.781	141.219	2	3	0.0057	0.0211	0.0057	0.0214	0.2664	0.2103	-1.2109	0.8870
SE0216	SERP2364	2.330294	868.132	346.868	868.932	346.068	869.620	345.380	2	3	0.0023	0.0087	0.0023	0.0087	0.2644	0.2091	-1.2191	0.8886
SE0474	SERP0361	2.330294	969.684	377.316	969.684	377.316	970.773	376.227	2	3	0.0021	0.0080	0.0021	0.0080	0.2625	0.2079	-1.2194	0.8887

SE1740	SERP1750	2.330294	988.338	385.662	988.038	385.962	989.276	384.724	2	3	0.0020	0.0078	0.0020	0.0078	0.2564	0.2041	−1.2307	0.8908
SE2199	SERP2210	2.330294	1682.076	648.924	1679.677	651.323	1681.965	649.035	7	6	0.0042	0.0092	0.0042	0.0093	0.4516	0.3111	−1.2369	0.8919
SE0631	SERP0523	2.330294	230.814	87.186	231.814	86.186	232.402	85.598	2	3	0.0086	0.0346	0.0087	0.0354	0.2458	0.1973	−1.2428	0.8930
SE1607	SERP1460	2.330294	1273.391	481.609	1272.992	482.008	1274.280	480.720	2	3	0.0016	0.0062	0.0016	0.0063	0.2540	0.2025	−1.2486	0.8941
SE1772	SERP1781	2.330294	1406.515	552.485	1407.116	551.884	1407.904	551.096	2	3	0.0014	0.0054	0.0014	0.0055	0.2545	0.2029	−1.2587	0.8959
SE1881	SERP1891	2.330294	568.434	205.566	568.084	205.916	569.348	204.652	2	3	0.0035	0.0146	0.0035	0.0147	0.2381	0.1923	−1.2641	0.8969
SE0867	SERP0757	2.330294	468.661	185.339	469.760	184.240	470.298	183.702	5	5	0.0107	0.0271	0.0107	0.0276	0.3877	0.2794	−1.2710	0.8981
SE0273	SERP2304	2.330294	407.522	168.478	406.122	169.878	406.822	169.178	3	4	0.0074	0.0236	0.0074	0.0240	0.3083	0.2357	−1.2927	0.9019
SE1981	SERP1994	2.330294	1049.172	423.828	1049.322	423.678	1050.335	422.665	3	4	0.0029	0.0094	0.0029	0.0095	0.3053	0.2339	−1.2961	0.9025
SE0202	SERP2374	2.330294	578.845	234.155	579.769	233.231	580.395	232.605	3	4	0.0052	0.0171	0.0052	0.0173	0.3006	0.2311	−1.3148	0.9057
SE1109	SERP0992	2.330294	430.404	181.596	431.653	180.347	431.028	180.972	13.3	10.8	0.0307	0.0594	0.0314	0.0619	0.5073	0.3365	−1.3177	0.9062
SE1266	SERP1147	2.330294	799.281	319.719	799.581	319.419	800.519	318.481	3	4	0.0038	0.0125	0.0038	0.0126	0.3016	0.2317	−1.3187	0.9064
SE0634	SERP0526	2.330294	254.672	102.328	253.695	103.305	255.272	101.728	3	4	0.0118	0.0389	0.0119	0.0399	0.2982	0.2297	−1.3196	0.9065
SE1794	SERP1802	2.330294	622.739	235.261	621.739	236.261	623.328	234.672	3	4	0.0048	0.0170	0.0048	0.0172	0.2791	0.2182	−1.3710	0.9148
SE0849	SERP0739	2.330294	94.507	37.493	94.507	37.493	95.595	36.405	0	2	0.0000	0.0533	0.0000	0.0553	0.0000	0.0000	−1.3791	0.9161
SE0259	SERP2320	2.330294	1128.688	461.312	1128.688	461.312	1129.776	460.224	0	2	0.0000	0.0043	0.0000	0.0043	0.0000	0.0000	−1.3871	0.9173
SE1744	SERP1753	2.330294	106.834	40.166	106.834	40.166	107.922	39.078	0	2	0.0000	0.0498	0.0000	0.0515	0.0000	0.0000	−1.3919	0.9180
SE1990	SERP2002	2.330294	103.830	40.170	103.830	40.170	104.918	39.082	0	2	0.0000	0.0498	0.0000	0.0515	0.0000	0.0000	−1.3919	0.9180
SE0834	SERP0724	2.330294	191.036	72.964	191.586	72.414	192.399	71.601	0	2	0.0000	0.0275	0.0000	0.0280	0.0000	0.0000	−1.3930	0.9182
SE1548	SERP1401	2.330294	909.673	377.327	910.296	376.704	911.073	375.927	0	2	0.0000	0.0053	0.0000	0.0053	0.0000	0.0000	−1.3947	0.9185
SE0473	SERP0360	2.330294	836.275	333.725	836.275	333.725	837.363	332.637	0	2	0.0000	0.0060	0.0000	0.0060	0.0000	0.0000	−1.3953	0.9185
SE0406	SERP0291	2.330294	586.387	247.613	586.387	247.613	587.475	246.525	0	2	0.0000	0.0081	0.0000	0.0081	0.0000	0.0000	−1.3966	0.9187
SE0208	SERP2370	2.330294	628.758	247.242	628.758	247.242	629.846	246.154	0	2	0.0000	0.0081	0.0000	0.0081	0.0000	0.0000	−1.3966	0.9187
SE1093	SERP0980	2.330294	145.909	55.091	145.909	55.091	146.997	54.003	0	2	0.0000	0.0363	0.0000	0.0372	0.0000	0.0000	−1.3985	0.9190
SE0472	SERP0359	2.330294	1378.410	571.590	1378.410	571.590	1379.498	570.502	0	2	0.0000	0.0035	0.0000	0.0035	0.0000	0.0000	−1.4000	0.9192
SE1339	SERP1228	2.330294	1908.921	719.079	1908.921	719.079	1910.009	717.991	0	2	0.0000	0.0028	0.0000	0.0028	0.0000	0.0000	−1.4000	0.9192
SE2407	SERP0014	2.330294	798.680	317.320	798.680	317.320	799.769	316.231	0	2	0.0000	0.0063	0.0000	0.0063	0.0000	0.0000	−1.4000	0.9192
SE0136	SERP2429	2.330294	218.703	93.297	218.703	93.297	219.791	92.209	0	2	0.0000	0.0214	0.0000	0.0217	0.0000	0.0000	−1.4000	0.9192
SE0788	SERP0677	2.330294	155.436	60.564	155.436	60.564	156.524	59.476	0	2	0.0000	0.0330	0.0000	0.0338	0.0000	0.0000	−1.4025	0.9196
SE0405	SERP0290	2.330294	675.274	251.726	675.274	251.726	676.362	250.638	0	2	0.0000	0.0079	0.0000	0.0080	0.0000	0.0000	−1.4035	0.9198
SE0907	SERP0798	2.330294	165.713	65.287	165.713	65.287	166.802	64.198	0	2	0.0000	0.0306	0.0000	0.0313	0.0000	0.0000	−1.4036	0.9198
SE1767	SERP1776	2.330294	726.957	302.043	726.656	302.344	727.895	301.105	0	2	0.0000	0.0066	0.0000	0.0066	0.0000	0.0000	−1.4043	0.9199
SE1595	SERP1448	2.330294	442.472	181.528	442.472	181.528	443.560	180.440	0	2	0.0000	0.0110	0.0000	0.0111	0.0000	0.0000	−1.4051	0.9200
SE0225	SERP2355	2.330294	451.298	193.702	451.298	193.702	452.387	192.613	0	2	0.0000	0.0103	0.0000	0.0104	0.0000	0.0000	−1.4054	0.9200
SE1438	SERP1325	2.330294	324.194	134.806	323.344	135.656	324.857	134.143	0	2	0.0000	0.0148	0.0000	0.0149	0.0000	0.0000	−1.4057	0.9201
SE1449	SERP1336	2.330294	503.885	207.115	503.885	207.115	504.973	206.027	0	2	0.0000	0.0097	0.0000	0.0097	0.0000	0.0000	−1.4058	0.9201
SE0686	SERP0576	2.330294	275.776	117.224	275.776	117.224	276.864	116.136	0	2	0.0000	0.0171	0.0000	0.0173	0.0000	0.0000	−1.4065	0.9202
SE0900	SERP0791	2.330294	265.586	106.414	265.586	106.414	266.674	105.326	0	2	0.0000	0.0188	0.0000	0.0190	0.0000	0.0000	−1.4074	0.9203
SE1087	SERP0974	2.330294	191.387	75.613	191.387	75.613	192.475	74.525	0	2	0.0000	0.0265	0.0000	0.0269	0.0000	0.0000	−1.4084	0.9205

SE2082	SERP2096	2.330294	719.151	321.849	719.151	321.849	720.239	320.761	0	2	0.0000	0.0062	0.0000	0.0062	0.0000	0.0000	−1.4091	0.9206
SE1795	SERP1803	2.330294	573.464	233.536	573.464	233.536	574.552	232.448	0	2	0.0000	0.0086	0.0000	0.0086	0.0000	0.0000	−1.4098	0.9207
SE0838	SERP0728	2.330294	222.370	89.630	222.370	89.630	223.458	88.542	0	2	0.0000	0.0223	0.0000	0.0227	0.0000	0.0000	−1.4099	0.9207
SE1942	SERP1954	2.330294	962.692	390.308	961.992	391.008	963.430	389.570	4	5	0.0042	0.0128	0.0042	0.0129	0.3256	0.2456	−1.4104	0.9208
SE1265	SERP1146	2.330294	680.401	255.599	680.401	255.599	681.489	254.511	0	2	0.0000	0.0078	0.0000	0.0079	0.0000	0.0000	−1.4107	0.9208
SE1829	SERP1838	2.330294	605.332	255.668	605.332	255.668	606.420	254.580	0	2	0.0000	0.0078	0.0000	0.0079	0.0000	0.0000	−1.4107	0.9208
SE0198	SERP2378	2.330294	491.337	195.663	491.037	195.963	492.275	194.725	0	2	0.0000	0.0102	0.0000	0.0103	0.0000	0.0000	−1.4110	0.9209
SE0804	SERP0693	2.330294	440.322	168.678	440.322	168.678	441.410	167.590	0	2	0.0000	0.0119	0.0000	0.0120	0.0000	0.0000	−1.4118	0.9210
SE2124	SERP1880	2.330294	545.224	225.776	545.224	225.776	546.312	224.688	0	2	0.0000	0.0089	0.0000	0.0089	0.0000	0.0000	−1.4127	0.9211
SE2014	SERP2027	2.330294	486.778	212.222	486.078	212.922	486.428	212.572	1	3	0.0021	0.0141	0.0021	0.0142	0.1479	0.1288	−1.4133	0.9212
SE2000	SERP2013	2.330294	508.760	190.240	508.760	190.240	509.848	189.152	0	2	0.0000	0.0105	0.0000	0.0106	0.0000	0.0000	−1.4133	0.9212
SE2140	SERP2151	2.330294	613.137	244.863	613.438	244.562	614.376	243.624	0	2	0.0000	0.0082	0.0000	0.0082	0.0000	0.0000	−1.4138	0.9213
SE0600	SERP0489	2.330294	726.726	296.274	728.275	294.725	728.589	294.411	4	5	0.0055	0.0169	0.0055	0.0171	0.3216	0.2434	−1.4158	0.9216
SE0189	SERP2385	2.330294	980.577	399.423	982.278	397.722	981.427	398.573	9	8	0.0092	0.0201	0.0092	0.0203	0.4532	0.3119	−1.4160	0.9216
SE1221	SERP1101	2.330294	951.464	392.536	951.464	392.536	952.553	391.447	0	2	0.0000	0.0051	0.0000	0.0051	0.0000	0.0000	−1.4167	0.9217
SE1827	SERP1835	2.330294	935.903	396.097	935.903	396.097	936.991	395.009	0	2	0.0000	0.0050	0.0000	0.0051	0.0000	0.0000	−1.4167	0.9217
SE2130	SERP2142	2.330294	982.378	394.622	982.378	394.622	983.466	393.534	0	2	0.0000	0.0051	0.0000	0.0051	0.0000	0.0000	−1.4167	0.9217
SE2258	SERP2288	2.330294	1096.032	436.968	1096.831	436.169	1097.520	435.480	4	5	0.0036	0.0115	0.0037	0.0115	0.3217	0.2434	−1.4175	0.9218
SE1933	SERP1945	2.330294	465.228	179.772	465.228	179.772	466.317	178.683	0	2	0.0000	0.0111	0.0000	0.0112	0.0000	0.0000	−1.4177	0.9219
SE2017	SERP2030	2.330294	440.703	192.297	440.403	192.597	441.641	191.359	0	2	0.0000	0.0104	0.0000	0.0105	0.0000	0.0000	−1.4189	0.9220
SE2089	SERP2103	2.330294	1149.894	452.106	1149.894	452.106	1150.982	451.018	0	2	0.0000	0.0044	0.0000	0.0044	0.0000	0.0000	−1.4194	0.9221
SE1885	SERP1895	2.330294	481.866	205.134	481.943	205.057	482.993	204.007	0	2	0.0000	0.0098	0.0000	0.0098	0.0000	0.0000	−1.4203	0.9222
SE2136	SERP2148	2.330294	955.648	373.352	955.648	373.352	956.736	372.264	0	2	0.0000	0.0054	0.0000	0.0054	0.0000	0.0000	−1.4211	0.9223
SE0411	SERP0296	2.330294	570.688	221.312	570.661	221.339	571.763	220.237	0	2	0.0000	0.0090	0.0000	0.0091	0.0000	0.0000	−1.4219	0.9225
SE1751	SERP1760	2.330294	1262.462	540.538	1262.462	540.538	1263.550	539.450	0	2	0.0000	0.0037	0.0000	0.0037	0.0000	0.0000	−1.4231	0.9226
SE1555	SERP1409	2.330294	1085.314	429.686	1085.314	429.686	1086.402	428.598	0	2	0.0000	0.0047	0.0000	0.0047	0.0000	0.0000	−1.4242	0.9228
SE1959	SERP1968	2.330294	1017.485	425.515	1017.485	425.515	1018.574	424.426	0	2	0.0000	0.0047	0.0000	0.0047	0.0000	0.0000	−1.4242	0.9228
SE0916	SERP0807	2.330294	241.586	106.414	241.886	106.114	242.824	105.176	0	2	0.0000	0.0188	0.0000	0.0191	0.0000	0.0000	−1.4254	0.9230
SE0816	SERP0707	2.330294	321.743	137.257	321.443	137.557	322.681	136.319	0	2	0.0000	0.0146	0.0000	0.0147	0.0000	0.0000	−1.4272	0.9232
SE1680	SERP1689	2.330294	972.122	380.878	972.122	380.878	973.210	379.790	0	2	0.0000	0.0053	0.0000	0.0053	0.0000	0.0000	−1.4324	0.9240
SE2354	SERP0063	2.330294	972.799	413.201	972.799	413.201	973.887	412.113	0	2	0.0000	0.0048	0.0000	0.0049	0.0000	0.0000	−1.4412	0.9252
SE0613	SERP0507	2.330294	729.947	293.053	729.920	293.080	731.022	291.978	4	5	0.0055	0.0171	0.0055	0.0173	0.3179	0.2412	−1.4461	0.9259
SE1199	SERP1079	2.330294	1010.518	408.482	1009.969	409.031	1011.332	407.668	1	3	0.0010	0.0073	0.0010	0.0074	0.1351	0.1190	−1.4497	0.9264
SE2400	SERP0021	2.330294	539.111	216.889	539.411	216.589	540.349	215.651	1	3	0.0019	0.0138	0.0019	0.0140	0.1357	0.1195	−1.4544	0.9271
SE0254	SERP2326	2.330294	688.771	262.229	687.771	263.229	689.360	261.640	1	3	0.0015	0.0114	0.0015	0.0115	0.1304	0.1154	−1.4565	0.9274
SE2232	SERP2264	2.330294	1456.506	571.494	1456.382	571.618	1456.444	571.556	7	7	0.0048	0.0122	0.0048	0.0123	0.3902	0.2807	−1.4569	0.9274
SE2117	SERP2129	2.330294	616.383	250.617	616.383	250.617	617.472	249.528	1	3	0.0016	0.0120	0.0016	0.0121	0.1322	0.1168	−1.4623	0.9282
SE1737	SERP1747	2.330294	508.253	199.747	508.553	199.447	509.491	198.509	1	3	0.0020	0.0150	0.0020	0.0152	0.1316	0.1163	−1.4627	0.9282
SE0267	SERP2311	2.330294	370.624	145.376	370.624	145.376	371.713	144.287	1	3	0.0027	0.0206	0.0027	0.0209	0.1292	0.1144	−1.4680	0.9290

SE2214	SERP2246	2.330294	491.667	192.333	490.790	193.210	492.317	191.683	1	3	0.0020	0.0156	0.0020	0.0157	0.1274	0.1130	−1.4704	0.9293
SE1681	SERP1690	2.330294	767.354	303.646	767.354	303.646	768.442	302.558	1	3	0.0013	0.0099	0.0013	0.0099	0.1313	0.1161	−1.4710	0.9294
SE0193	SERP2383	2.330294	715.009	277.991	715.310	277.690	716.248	276.752	1	3	0.0014	0.0108	0.0014	0.0109	0.1284	0.1138	−1.4720	0.9295
SE0201	SERP2375	2.330294	607.371	229.629	606.344	230.656	607.946	229.054	4	5	0.0066	0.0217	0.0066	0.0220	0.3000	0.2308	−1.4757	0.9300
SE1871	SERP1879	2.330294	1426.461	544.539	1426.912	544.088	1427.775	543.225	4	5	0.0028	0.0092	0.0028	0.0092	0.3043	0.2333	−1.4772	0.9302
SE2397	SERP0024	2.330294	414.923	161.077	415.724	160.276	416.412	159.588	1	3	0.0024	0.0187	0.0024	0.0189	0.1270	0.1127	−1.4783	0.9303
SE0494	SERP0379	2.330294	1381.072	556.928	1381.222	556.778	1382.235	555.765	1	3	0.0007	0.0054	0.0007	0.0054	0.1296	0.1148	−1.4789	0.9304
SE2012	SERP2025	2.330294	545.364	210.636	545.364	210.636	546.453	209.547	1	3	0.0018	0.0142	0.0018	0.0144	0.1250	0.1111	−1.4836	0.9310
SE0999	SERP0887	2.330294	521.640	207.360	524.013	204.987	522.826	206.174	13.5	10.5	0.0258	0.0509	0.0263	0.0527	0.4991	0.3329	−1.4858	0.9313
SE1588	SERP1441	2.330294	872.467	333.533	872.767	333.233	873.705	332.295	1	3	0.0011	0.0090	0.0011	0.0091	0.1209	0.1078	−1.5052	0.9339
SE2154	SERP2165	2.330294	967.985	391.015	969.436	389.564	969.799	389.201	5	6	0.0052	0.0154	0.0052	0.0155	0.3355	0.2512	−1.5145	0.9351
SE1947	SERP1959	2.330294	464.785	180.215	464.382	180.618	465.672	179.328	7	7	0.0151	0.0388	0.0152	0.0398	0.3819	0.2764	−1.5154	0.9352
SE0835	SERP0725	2.330294	367.720	151.280	367.970	151.030	368.933	150.067	5	6	0.0136	0.0397	0.0137	0.0408	0.3358	0.2514	−1.5162	0.9353
SE0330	SERP0206	2.330294	398.931	165.069	398.931	165.069	400.019	163.981	2	4	0.0050	0.0242	0.0050	0.0246	0.2033	0.1689	−1.5212	0.9359
SE0248	SERP2333	2.330294	1207.422	493.578	1207.898	493.102	1208.748	492.252	2	4	0.0017	0.0081	0.0017	0.0082	0.2073	0.1717	−1.5215	0.9359
SE0182	SERP2393	2.330294	484.638	199.362	484.939	199.061	485.877	198.123	2	4	0.0041	0.0201	0.0041	0.0204	0.2010	0.1673	−1.5371	0.9379
SE0266	SERP2312	2.330294	1067.971	426.029	1067.820	426.180	1068.984	425.016	2	4	0.0019	0.0094	0.0019	0.0094	0.2021	0.1681	−1.5380	0.9380
SE1521	SERP1376	2.330294	703.709	271.291	704.009	270.991	704.947	270.053	2	4	0.0028	0.0148	0.0028	0.0149	0.1879	0.1582	−1.5589	0.9405
SE1463	SERP1357	2.330294	719.285	279.715	719.162	279.838	720.312	278.688	5	6	0.0070	0.0214	0.0070	0.0218	0.3211	0.2431	−1.5704	0.9418
SE2094	SERP2108	2.330294	227.116	87.884	226.365	88.635	226.740	88.260	5	6	0.0221	0.0680	0.0224	0.0713	0.3142	0.2391	−1.5715	0.9420
SE2356	SERP0061	2.330294	546.535	230.465	546.835	230.165	547.773	229.227	3	5	0.0055	0.0217	0.0055	0.0220	0.2500	0.2000	−1.5859	0.9436
SE0211	SERP2367	2.330294	841.621	346.379	842.744	345.256	843.271	344.729	3	5	0.0036	0.0145	0.0036	0.0146	0.2466	0.1978	−1.5882	0.9439
SE0348	SERP0222	2.330294	191.611	66.389	190.762	67.238	192.275	65.725	2	4	0.0105	0.0599	0.0105	0.0624	0.1683	0.1440	−1.5991	0.9451
SE2247	SERP2279	2.330294	1124.694	429.306	1124.394	429.606	1125.632	428.368	8	8	0.0071	0.0186	0.0071	0.0189	0.3757	0.2731	−1.6501	0.9505
SE2075	SERP2088	2.330294	584.017	222.983	583.141	223.859	584.667	222.333	6	7	0.0103	0.0313	0.0104	0.0320	0.3250	0.2453	−1.6741	0.9529
SE1587	SERP1440	2.330294	1082.876	450.124	1083.876	449.124	1084.464	448.536	4	6	0.0037	0.0133	0.0037	0.0135	0.2741	0.2151	−1.6934	0.9548
SE0272	SERP2305	2.330294	885.213	356.787	886.213	355.787	886.801	355.199	4	6	0.0045	0.0168	0.0045	0.0170	0.2647	0.2093	−1.6965	0.9551
SE0720	SERP0609	2.330294	1131.370	428.630	1131.370	428.630	1132.458	427.542	0	3	0.0000	0.0070	0.0000	0.0070	0.0000	0.0000	−1.7073	0.9561
SE1248	SERP1127	2.330294	784.152	319.848	784.152	319.848	785.240	318.760	0	3	0.0000	0.0094	0.0000	0.0094	0.0000	0.0000	−1.7091	0.9563
SE1603	SERP1456	2.330294	984.320	392.680	983.269	393.731	984.882	392.118	4	6	0.0041	0.0153	0.0041	0.0154	0.2662	0.2103	−1.7096	0.9563
SE0782	SERP0670	2.330294	1218.277	497.723	1217.727	498.273	1219.090	496.910	0	3	0.0000	0.0060	0.0000	0.0060	0.0000	0.0000	−1.7143	0.9568
SE1870	SERP1878	2.330294	515.896	210.104	515.896	210.104	516.985	209.015	0	3	0.0000	0.0143	0.0000	0.0144	0.0000	0.0000	−1.7143	0.9568
SE0638	SERP0530	2.330294	266.618	105.382	266.618	105.382	267.707	104.293	0	3	0.0000	0.0285	0.0000	0.0290	0.0000	0.0000	−1.7160	0.9569
SE0566	SERP0451	2.330294	328.445	139.555	328.445	139.555	329.533	138.467	0	3	0.0000	0.0215	0.0000	0.0218	0.0000	0.0000	−1.7165	0.9570
SE0561	SERP0446	2.330294	918.988	383.012	918.988	383.012	920.076	381.924	0	3	0.0000	0.0078	0.0000	0.0079	0.0000	0.0000	−1.7174	0.9570
SE1012	SERP0900	2.330294	578.189	225.811	578.189	225.811	579.277	224.723	0	3	0.0000	0.0133	0.0000	0.0134	0.0000	0.0000	−1.7179	0.9571
SE2412	SERP0009	2.330294	253.762	103.238	253.762	103.238	254.850	102.150	0	3	0.0000	0.0291	0.0000	0.0296	0.0000	0.0000	−1.7209	0.9574
SE0229	SERP2351	2.330294	712.625	286.375	712.625	286.375	713.714	285.286	0	3	0.0000	0.0105	0.0000	0.0105	0.0000	0.0000	−1.7213	0.9574
SE0407	SERP0292	2.330294	524.666	219.334	524.666	219.334	525.754	218.246	0	3	0.0000	0.0137	0.0000	0.0138	0.0000	0.0000	−1.7250	0.9577

SE0560	SERP0445	2.330294	1077.075	437.925	1077.075	437.925	1078.163	436.837	0	3	0.0000	0.0069	0.0000	0.0069	0.0000	0.0000	−1.7250	0.9577
SE2417	SERP0003	2.330294	981.911	395.089	982.212	394.788	983.150	393.850	0	3	0.0000	0.0076	0.0000	0.0076	0.0000	0.0000	−1.7273	0.9579
SE1585	SERP1438	2.330294	1021.867	433.133	1021.867	433.133	1022.955	432.045	1	4	0.0010	0.0092	0.0010	0.0093	0.1075	0.0971	−1.7273	0.9579
SE2195	SERP2206	2.330294	472.590	190.410	472.590	190.410	473.678	189.322	0	3	0.0000	0.0158	0.0000	0.0159	0.0000	0.0000	−1.7283	0.9580
SE0223	SERP2357	2.330294	443.599	189.401	443.299	189.701	444.537	188.463	1	4	0.0023	0.0211	0.0023	0.0214	0.1075	0.0970	−1.7297	0.9582
SE2256	SERP2286	2.330294	677.650	276.350	677.650	276.350	678.738	275.262	0	3	0.0000	0.0109	0.0000	0.0109	0.0000	0.0000	−1.7302	0.9582
SE0224	SERP2356	2.330294	679.349	277.651	679.349	277.651	680.437	276.563	0	3	0.0000	0.0108	0.0000	0.0109	0.0000	0.0000	−1.7302	0.9582
SE1028	SERP0917	2.330294	810.231	311.769	810.231	311.769	811.319	310.681	0	3	0.0000	0.0096	0.0000	0.0097	0.0000	0.0000	−1.7321	0.9584
SE2146	SERP2157	2.330294	632.222	246.778	632.523	246.477	633.461	245.539	0	3	0.0000	0.0122	0.0000	0.0123	0.0000	0.0000	−1.7324	0.9584
SE0648	SERP0540	2.330294	427.328	163.672	427.328	163.672	428.416	162.584	0	3	0.0000	0.0183	0.0000	0.0186	0.0000	0.0000	−1.7383	0.9589
SE2159	SERP2169	2.330294	577.618	238.382	577.968	238.032	578.881	237.119	0	3	0.0000	0.0126	0.0000	0.0127	0.0000	0.0000	−1.7397	0.9590
SE0244	SERP2337	2.330294	444.787	176.213	447.160	173.840	445.973	175.027	11.5	10.5	0.0258	0.0600	0.0262	0.0625	0.4192	0.2954	−1.7484	0.9598
SE2358	SERP0059	2.330294	1078.651	442.349	1078.350	442.650	1079.589	441.411	1	4	0.0009	0.0090	0.0009	0.0091	0.0989	0.0900	−1.7494	0.9599
SE1428	SERP1315	2.330294	899.309	360.691	899.009	360.991	900.247	359.753	0	3	0.0000	0.0083	0.0000	0.0084	0.0000	0.0000	−1.7500	0.9599
SE1580	SERP1433	2.330294	759.431	308.569	759.581	308.419	760.594	307.406	1	4	0.0013	0.0130	0.0013	0.0131	0.0992	0.0903	−1.7542	0.9603
SE1271	SERP1152	2.330294	1298.020	522.980	1298.020	522.980	1299.108	521.892	0	3	0.0000	0.0057	0.0000	0.0058	0.0000	0.0000	−1.7576	0.9606
SE0507	SERP0390	2.330294	660.322	260.678	660.996	260.004	661.747	259.253	1	4	0.0015	0.0154	0.0015	0.0155	0.0968	0.0882	−1.7626	0.9610
SE0855	SERP0745	2.330294	287.190	111.810	287.190	111.810	288.278	110.722	1	4	0.0035	0.0358	0.0035	0.0367	0.0954	0.0871	−1.7633	0.9611
SE2377	SERP0039	2.330294	620.835	252.165	621.135	251.865	622.073	250.927	1	4	0.0016	0.0159	0.0016	0.0160	0.1000	0.0909	−1.7650	0.9612
SE1574	SERP1427	2.330294	695.353	288.647	695.326	288.674	696.428	287.572	1	4	0.0014	0.0139	0.0014	0.0140	0.1000	0.0909	−1.7650	0.9612
SE0347	SERP0221	2.330294	263.922	96.078	263.372	96.628	264.735	95.265	1	4	0.0038	0.0415	0.0038	0.0427	0.0890	0.0817	−1.7658	0.9613
SE1932	SERP1944	2.330294	1412.551	561.449	1412.851	561.149	1413.789	560.211	1	4	0.0007	0.0071	0.0007	0.0072	0.0972	0.0886	−1.7724	0.9618
SE2095	SERP2109	2.330294	697.993	280.007	695.593	282.407	697.881	280.119	1	4	0.0014	0.0142	0.0014	0.0144	0.0972	0.0886	−1.7724	0.9618
SE0717	SERP0606	2.330294	848.632	324.368	848.233	324.767	849.521	323.479	1	4	0.0012	0.0123	0.0012	0.0124	0.0968	0.0882	−1.7735	0.9619
SE0684	SERP0574	2.330294	1171.302	469.698	1170.452	470.548	1170.877	470.123	8	9	0.0068	0.0191	0.0069	0.0194	0.3557	0.2624	−1.7760	0.9621
SE2409	SERP0012	2.330294	416.200	150.800	415.650	151.350	417.013	149.987	1	4	0.0024	0.0265	0.0024	0.0270	0.0889	0.0816	−1.7813	0.9626
SE1970	SERP1982	2.330294	743.977	288.023	743.526	288.474	744.840	287.160	1	4	0.0013	0.0139	0.0013	0.0140	0.0929	0.0850	−1.7838	0.9628
SE1964	SERP1976	2.330294	898.011	379.989	897.861	380.139	899.024	378.976	2	5	0.0022	0.0132	0.0022	0.0133	0.1654	0.1419	−1.7875	0.9631
SE1736	SERP1745	2.330294	468.732	191.268	468.732	191.268	468.732	191.268	2	5	0.0043	0.0261	0.0043	0.0266	0.1617	0.1392	−1.8028	0.9643
SE0269	SERP2308	2.330294	297.377	116.623	298.051	115.949	297.714	116.286	2	6	0.0067	0.0516	0.0067	0.0535	0.1252	0.1113	−1.8036	0.9644
SE0197	SERP2379	2.330294	547.438	223.562	547.438	223.562	548.526	222.474	2	5	0.0037	0.0224	0.0037	0.0227	0.1630	0.1402	−1.8050	0.9645
SE0275	SERP2302	2.330294	498.785	203.215	499.184	202.816	500.073	201.927	2	5	0.0040	0.0246	0.0040	0.0250	0.1600	0.1379	−1.8190	0.9655
SE0274	SERP2303	2.330294	413.029	162.971	412.330	163.670	413.768	162.232	2	5	0.0048	0.0306	0.0049	0.0313	0.1565	0.1354	−1.8202	0.9656
SE0898	SERP0789	2.330294	451.531	181.469	449.681	183.319	450.606	182.394	11	11	0.0244	0.0603	0.0248	0.0629	0.3943	0.2828	−1.8203	0.9656
SE0685	SERP0575	2.330294	705.500	281.500	706.049	280.951	706.863	280.137	2	5	0.0028	0.0178	0.0028	0.0180	0.1556	0.1346	−1.8218	0.9658
SE2257	SERP2287	2.330294	1114.242	433.758	1113.942	434.058	1115.180	432.820	2	5	0.0018	0.0115	0.0018	0.0116	0.1552	0.1343	−1.8283	0.9663
SE1064	SERP0954	2.330294	244.435	91.565	244.135	91.865	245.374	90.626	2	5	0.0082	0.0545	0.0082	0.0566	0.1449	0.1265	−1.8303	0.9664
SE0271	SERP2306	2.330294	431.381	180.619	430.708	181.292	432.133	179.867	3	6	0.0070	0.0332	0.0070	0.0339	0.2065	0.1711	−1.8598	0.9685
SE0018	SERP2534	2.330294	496.580	202.420	496.881	202.119	496.730	202.270	0	5	0.0000	0.0247	0.0000	0.0251	0.0000	0.0000	−1.8731	0.9695

SE1062	SERP0952	2.330294	608.112	243.888	609.536	242.464	609.912	242.088	6	8	0.0099	0.0329	0.0099	0.0336	0.2946	0.2276	−1.8736	0.9695
SE0354	SERP0232	2.330294	1054.592	433.408	1053.343	434.657	1055.056	432.944	3	6	0.0028	0.0138	0.0029	0.0140	0.2071	0.1716	−1.8749	0.9696
SE0019	SERP2533	2.330294	1320.357	509.643	1320.357	509.643	1321.445	508.555	0	4	0.0000	0.0078	0.0000	0.0079	0.0000	0.0000	−1.9750	0.9759
SE2376	SERP0040	2.330294	150.662	50.338	150.662	50.338	151.750	49.250	0	4	0.0000	0.0795	0.0000	0.0840	0.0000	0.0000	−1.9765	0.9759
SE0853	SERP0743	2.330294	315.247	113.753	315.274	113.726	316.349	112.651	0	4	0.0000	0.0352	0.0000	0.0360	0.0000	0.0000	−1.9780	0.9760
SE0509	SERP0392	2.330294	1071.681	431.319	1071.681	431.319	1072.769	430.231	0	4	0.0000	0.0093	0.0000	0.0093	0.0000	0.0000	−1.9787	0.9761
SE0215	SERP2365	2.330294	535.419	217.581	534.869	218.131	536.232	216.768	0	4	0.0000	0.0184	0.0000	0.0186	0.0000	0.0000	−1.9787	0.9761
SE0890	SERP0781	2.330294	347.001	138.999	347.001	138.999	348.089	137.911	0	4	0.0000	0.0288	0.0000	0.0293	0.0000	0.0000	−1.9797	0.9761
SE0217	SERP2363	2.330294	949.437	406.563	949.737	406.263	950.675	405.325	0	4	0.0000	0.0098	0.0000	0.0099	0.0000	0.0000	−1.9800	0.9761
SE1207	SERP1087	2.330294	973.430	382.570	973.129	382.871	974.368	381.632	0	4	0.0000	0.0105	0.0000	0.0105	0.0000	0.0000	−1.9811	0.9762
SE1803	SERP1811	2.330294	913.196	376.804	913.196	376.804	914.284	375.716	0	4	0.0000	0.0106	0.0000	0.0107	0.0000	0.0000	−1.9815	0.9762
SE1458	SERP1352	2.330294	841.294	355.706	840.794	356.206	842.133	354.867	0	4	0.0000	0.0112	0.0000	0.0113	0.0000	0.0000	−1.9825	0.9763
SE1950	SERP1962	2.330294	703.338	286.662	703.364	286.636	704.439	285.561	0	4	0.0000	0.0140	0.0000	0.0141	0.0000	0.0000	−1.9859	0.9765
SE0053	SERP2503	2.330294	220.013	88.987	220.039	88.961	220.026	88.974	2	7	0.0091	0.0787	0.0091	0.0831	0.1095	0.0987	−1.9864	0.9765
SE0633	SERP0525	2.330294	173.270	69.730	173.570	69.430	174.508	68.492	0	4	0.0000	0.0575	0.0000	0.0598	0.0000	0.0000	−1.9867	0.9765
SE1019	SERP0906	2.330294	705.834	269.166	705.834	269.166	706.922	268.078	0	4	0.0000	0.0149	0.0000	0.0150	0.0000	0.0000	−2.0000	0.9772
SE0364	SERP0241	2.330294	240.900	98.100	240.250	98.750	241.663	97.337	1	5	0.0042	0.0508	0.0042	0.0526	0.0798	0.0739	−2.0109	0.9778
SE2175	SERP2186	2.330294	827.903	348.097	827.903	348.097	828.991	347.009	1	5	0.0012	0.0144	0.0012	0.0145	0.0828	0.0764	−2.0122	0.9779
SE0840	SERP0730	2.330294	437.127	174.873	436.977	175.023	438.140	173.860	1	5	0.0023	0.0286	0.0023	0.0291	0.0790	0.0732	−2.0150	0.9780
SE2157	SERP2167	2.330294	664.426	256.574	662.026	258.974	664.314	256.686	10	11	0.0151	0.0427	0.0152	0.0439	0.3462	0.2572	−2.0163	0.9781
SE0326	SERP0203	2.330294	444.034	170.966	444.034	170.966	445.122	169.878	1	5	0.0023	0.0292	0.0023	0.0298	0.0772	0.0717	−2.0227	0.9784
SE0222	SERP2358	2.330294	848.873	336.127	849.246	335.754	850.148	334.852	1	5	0.0012	0.0149	0.0012	0.0150	0.0800	0.0741	−2.0274	0.9787
SE1743	SERP1752	2.330294	677.717	258.283	677.717	258.283	678.805	257.195	1	5	0.0015	0.0194	0.0015	0.0196	0.0765	0.0711	−2.0276	0.9787
SE2088	SERP2102	2.330294	617.758	261.242	618.308	260.692	619.121	259.879	2	6	0.0032	0.0230	0.0032	0.0234	0.1368	0.1203	−2.0463	0.9796
SE0366	SERP0243	2.330294	298.002	121.998	298.079	121.921	299.128	120.872	2	6	0.0067	0.0492	0.0067	0.0509	0.1316	0.1163	−2.0518	0.9799
SE0850	SERP0740	2.330294	494.391	189.609	495.190	188.810	495.879	188.121	10	11	0.0202	0.0581	0.0205	0.0605	0.3388	0.2531	−2.0533	0.9800
SE1441	SERP1328	2.330294	740.822	300.178	739.672	301.328	741.335	299.665	2	6	0.0027	0.0199	0.0027	0.0202	0.1337	0.1179	−2.0553	0.9801
SE0368	SERP0250	2.330294	335.519	138.481	335.342	138.658	336.519	137.481	2	6	0.0060	0.0433	0.0060	0.0446	0.1345	0.1186	−2.0558	0.9801
SE1376	SERP1264	2.330294	615.066	239.934	615.066	239.934	616.154	238.846	2	6	0.0033	0.0250	0.0033	0.0254	0.1299	0.1150	−2.0560	0.9801
SE1088	SERP0975	2.330294	442.615	181.385	443.092	180.908	443.941	180.059	2	6	0.0045	0.0331	0.0045	0.0339	0.1327	0.1172	−2.0612	0.9804
SE2011	SERP2024	2.330294	1258.284	514.716	1258.761	514.239	1259.611	513.389	2	6	0.0016	0.0117	0.0016	0.0118	0.1356	0.1194	−2.0713	0.9808
SE1273	SERP1154	2.330294	692.255	279.745	692.255	279.745	693.343	278.657	2	6	0.0029	0.0214	0.0029	0.0218	0.1330	0.1174	−2.0719	0.9809
SE1785	SERP1793	2.330294	662.271	267.729	663.195	266.805	663.821	266.179	2	6	0.0030	0.0224	0.0030	0.0228	0.1316	0.1163	−2.0767	0.9811
SE2093	SERP2107	2.330294	824.899	330.101	824.350	330.650	825.713	329.287	2	6	0.0024	0.0182	0.0024	0.0184	0.1304	0.1154	−2.0806	0.9813
SE0694	SERP0585	2.330294	249.220	95.780	247.420	97.580	248.320	96.680	1.5	5.5	0.0060	0.0569	0.0061	0.0592	0.1030	0.0934	−2.0871	0.9816
SE0140	SERP2425	2.330294	625.789	256.211	627.339	254.661	626.564	255.436	3	7	0.0048	0.0274	0.0048	0.0279	0.1720	0.1468	−2.1070	0.9824
SE0500	SERP0383	2.330294	1343.284	537.716	1344.384	536.616	1344.922	536.078	3	7	0.0022	0.0130	0.0022	0.0131	0.1679	0.1438	−2.1099	0.9826
SE1989	SERP2001	2.330294	894.292	350.708	894.292	350.708	895.380	349.620	3	7	0.0034	0.0200	0.0034	0.0202	0.1683	0.1441	−2.1183	0.9829
SE0276	SERP2301	2.330294	536.640	219.360	536.190	219.810	537.503	218.497	3	7	0.0056	0.0319	0.0056	0.0326	0.1718	0.1466	−2.1244	0.9832

SE1379	SERP1267	2.330294	672.472	263.528	672.996	263.004	673.822	262.178	3	7	0.0045	0.0266	0.0045	0.0271	0.1661	0.1424	−2.1274	0.9833
SE1059	SERP0948	2.330294	547.066	214.934	545.192	216.808	546.129	215.871	11.5	13.5	0.0211	0.0625	0.0214	0.0653	0.3277	0.2468	−2.1657	0.9848
SE0693	SERP0583	2.330294	422.583	165.417	422.210	165.790	423.485	164.515	12	13	0.0284	0.0785	0.0290	0.0829	0.3498	0.2592	−2.1738	0.9851
SE2378	SERP0038	2.330294	597.716	242.284	598.265	241.735	599.079	240.921	4	8	0.0067	0.0331	0.0067	0.0338	0.1982	0.1654	−2.1775	0.9853
SE1377	SERP1265	2.330294	880.373	346.627	880.223	346.777	881.386	345.614	4	8	0.0045	0.0231	0.0046	0.0234	0.1966	0.1643	−2.1828	0.9855
SE1061	SERP0951	2.330294	557.118	216.882	557.641	216.359	558.468	215.532	7	10	0.0126	0.0462	0.0127	0.0476	0.2668	0.2106	−2.1895	0.9857
SE2375	SERP0041	2.330294	782.028	312.972	782.028	312.972	783.116	311.884	0	5	0.0000	0.0160	0.0000	0.0161	0.0000	0.0000	−2.2055	0.9863
SE2353	SERP0064	2.330294	645.453	254.547	645.453	254.547	646.541	253.459	0	5	0.0000	0.0196	0.0000	0.0199	0.0000	0.0000	−2.2111	0.9865
SE2083	SERP2098	2.330294	558.674	224.326	558.674	224.326	559.762	223.238	0	5	0.0000	0.0223	0.0000	0.0226	0.0000	0.0000	−2.2157	0.9866
SE2255	SERP2285	2.330294	546.259	224.741	546.259	224.741	547.347	223.653	0	5	0.0000	0.0222	0.0000	0.0226	0.0000	0.0000	−2.2157	0.9866
SE2076	SERP2089	2.330294	440.377	168.623	440.377	168.623	441.465	167.535	0	5	0.0000	0.0297	0.0000	0.0303	0.0000	0.0000	−2.2279	0.9871
SE2259	SERP2289	2.330294	827.178	345.822	826.628	346.372	827.991	345.009	1	6	0.0012	0.0173	0.0012	0.0175	0.0686	0.0642	−2.2331	0.9872
SE1945	SERP1957	2.330294	1111.079	487.921	1111.079	487.921	1112.167	486.833	0	5	0.0000	0.0102	0.0000	0.0103	0.0000	0.0000	−2.2391	0.9874
SE0876	SERP0766	2.330294	628.292	250.708	628.292	250.708	629.381	249.619	1	6	0.0016	0.0239	0.0016	0.0243	0.0658	0.0618	−2.2415	0.9875
SE0541	SERP0426	2.330294	1418.560	564.440	1418.710	564.290	1419.723	563.277	1	6	0.0007	0.0106	0.0007	0.0107	0.0654	0.0614	−2.2445	0.9876
SE1373	SERP1261	2.330294	1246.574	508.426	1246.574	508.426	1247.662	507.338	0	5	0.0000	0.0098	0.0000	0.0099	0.0000	0.0000	−2.2500	0.9878
SE1878	SERP1887	2.330294	220.338	94.662	220.215	94.785	221.365	93.635	1	6	0.0045	0.0633	0.0046	0.0662	0.0695	0.0650	−2.2504	0.9878
SE1549	SERP1402	2.330294	775.690	316.310	775.690	316.310	776.779	315.221	0	5	0.0000	0.0158	0.0000	0.0160	0.0000	0.0000	−2.2535	0.9879
SE0994	SERP0882	2.330294	490.410	190.590	489.959	191.041	491.273	189.727	8	11	0.0163	0.0576	0.0165	0.0600	0.2750	0.2157	−2.2548	0.9879
SE1096	SERP0985	2.330294	905.211	354.789	905.211	354.789	905.211	354.789	1	6	0.0011	0.0169	0.0011	0.0171	0.0643	0.0604	−2.2580	0.9880
SE0001	SERP2553	2.330294	972.286	380.714	972.835	380.165	973.649	379.351	1	6	0.0010	0.0158	0.0010	0.0159	0.0629	0.0592	−2.2657	0.9883
SE1969	SERP1981	2.330294	462.585	191.415	463.585	190.415	464.173	189.827	2	7	0.0043	0.0367	0.0043	0.0376	0.1144	0.1026	−2.2758	0.9886
SE1793	SERP1801	2.330294	575.406	228.594	575.105	228.895	576.344	227.656	2	7	0.0035	0.0306	0.0035	0.0312	0.1122	0.1009	−2.2780	0.9886
SE0227	SERP2353	2.330294	1110.237	452.763	1110.387	452.613	1111.400	451.600	2	7	0.0018	0.0155	0.0018	0.0156	0.1154	0.1034	−2.2842	0.9888
SE2197	SERP2208	2.330294	1057.870	409.130	1057.170	409.830	1058.608	408.392	2	7	0.0019	0.0171	0.0019	0.0173	0.1098	0.0990	−2.2893	0.9890
SE1999	SERP2012	2.330294	818.626	330.374	818.352	330.648	819.577	329.423	6	10	0.0073	0.0303	0.0074	0.0309	0.2395	0.1932	−2.2929	0.9891
SE2252	SERP2282	2.330294	941.876	357.124	942.176	356.824	943.114	355.886	2	7	0.0021	0.0196	0.0021	0.0199	0.1055	0.0955	−2.2978	0.9892
SE1079	SERP0969	2.330294	776.619	294.381	776.619	294.381	777.707	293.293	2	7	0.0026	0.0238	0.0026	0.0242	0.1074	0.0970	−2.3041	0.9894
SE0343	SERP0218	2.330294	461.343	183.657	461.793	183.207	461.568	183.432	11.5	13.5	0.0249	0.0736	0.0253	0.0775	0.3265	0.2461	−2.3178	0.9898
SE1948	SERP1960	2.330294	1548.653	614.347	1548.352	614.648	1549.591	613.409	2	7	0.0013	0.0114	0.0013	0.0115	0.1130	0.1016	−2.3218	0.9899
SE0185	SERP2388	2.330294	1386.777	542.223	1384.678	544.322	1385.727	543.273	16.5	16.5	0.0119	0.0304	0.0120	0.0310	0.3871	0.2791	−2.3254	0.9900
SE1873	SERP1881	2.330294	999.155	395.845	999.755	395.245	1000.543	394.457	3	8	0.0030	0.0202	0.0030	0.0205	0.1463	0.1277	−2.3348	0.9902
SE0894	SERP0785	2.330294	531.337	209.663	530.337	210.663	531.925	209.075	3	8	0.0057	0.0381	0.0057	0.0391	0.1458	0.1272	−2.3539	0.9907
SE0340	SERP0215	2.330294	515.441	213.559	515.392	213.608	515.416	213.584	17	18	0.0330	0.0843	0.0337	0.0894	0.3770	0.2738	−2.3605	0.9909
SE0845	SERP0735	2.330294	363.278	143.722	363.479	143.521	363.379	143.621	7	11	0.0193	0.0766	0.0195	0.0808	0.2413	0.1944	−2.3800	0.9913
SE1940	SERP1952	2.330294	764.572	285.428	764.572	285.428	765.661	284.339	3	8	0.0039	0.0280	0.0039	0.0286	0.1364	0.1200	−2.3845	0.9914
SE0889	SERP0780	2.330294	141.011	59.989	140.711	60.289	141.949	59.051	0	6	0.0000	0.0998	0.0000	0.1071	0.0000	0.0000	−2.3853	0.9915
SE0619	SERP0513	2.330294	192.987	71.013	192.438	71.562	193.801	70.199	0	6	0.0000	0.0842	0.0000	0.0893	0.0000	0.0000	−2.3941	0.9917
SE1006	SERP0893	2.330294	627.944	242.056	628.868	241.132	629.494	240.506	4	9	0.0064	0.0373	0.0064	0.0382	0.1675	0.1435	−2.4102	0.9920

SE0688	SERP0578	2.330294	528.562	191.438	528.012	191.988	529.375	190.625	0	6	0.0000	0.0313	0.0000	0.0320	0.0000	0.0000	−2.4242	0.9923
SE2348	SERP0069	2.330294	1033.413	430.587	1033.413	430.587	1034.502	429.498	0	6	0.0000	0.0139	0.0000	0.0141	0.0000	0.0000	−2.4310	0.9925
SE0678	SERP0568	2.330294	881.220	360.780	881.844	360.156	882.620	359.380	0	6	0.0000	0.0166	0.0000	0.0168	0.0000	0.0000	−2.4348	0.9925
SE0922	SERP0812	2.330294	548.188	219.812	548.162	219.838	549.263	218.737	0	6	0.0000	0.0273	0.0000	0.0278	0.0000	0.0000	−2.4386	0.9926
SE0319	SERP0196	2.330294	512.281	201.719	513.932	200.068	514.194	199.806	5	10	0.0097	0.0498	0.0098	0.0515	0.1903	0.1599	−2.4419	0.9927
SE2248	SERP2280	2.330294	852.007	344.993	852.557	344.443	853.370	343.630	0	6	0.0000	0.0174	0.0000	0.0176	0.0000	0.0000	−2.4444	0.9927
SE1053	SERP0942	2.330294	857.427	348.573	857.127	348.873	858.365	347.635	0	6	0.0000	0.0172	0.0000	0.0174	0.0000	0.0000	−2.4507	0.9929
SE1181	SERP1060	2.330294	630.056	254.944	629.206	255.794	630.719	254.281	1	7	0.0016	0.0274	0.0016	0.0279	0.0573	0.0542	−2.4533	0.9929
SE1100	SERP0989	2.330294	465.478	191.522	466.727	190.273	466.102	190.898	1	7	0.0021	0.0367	0.0021	0.0376	0.0559	0.0529	−2.4562	0.9930
SE0252	SERP2328	2.330294	1004.555	411.445	1004.306	411.694	1005.519	410.481	1	7	0.0010	0.0170	0.0010	0.0172	0.0581	0.0549	−2.4633	0.9931
SE0387	SERP0270	2.330294	361.272	145.728	361.272	145.728	361.272	145.728	2	8	0.0055	0.0549	0.0056	0.0570	0.0982	0.0895	−2.4748	0.9933
SE1946	SERP1958	2.330294	1199.885	477.115	1199.735	477.265	1200.898	476.102	1	7	0.0008	0.0147	0.0008	0.0148	0.0541	0.0513	−2.4749	0.9933
SE1735	SERP1744	2.330294	927.478	371.522	928.478	370.522	929.066	369.934	2	8	0.0022	0.0216	0.0022	0.0219	0.1005	0.0913	−2.4802	0.9934
SE1939	SERP1951	2.330294	473.880	192.120	474.580	191.420	475.318	190.682	2	8	0.0042	0.0417	0.0042	0.0429	0.0979	0.0892	−2.4821	0.9935
SE0327	SERP0204	2.330294	361.756	142.244	361.305	142.695	362.619	141.381	2	8	0.0055	0.0562	0.0056	0.0584	0.0959	0.0875	−2.4834	0.9935
SE2158	SERP2168	2.330294	1071.776	425.224	1072.077	424.923	1073.015	423.985	2	8	0.0019	0.0188	0.0019	0.0191	0.0995	0.0905	−2.4844	0.9935
SE0636	SERP0528	2.330294	1051.270	427.730	1052.120	426.880	1051.695	427.305	9.5	13.5	0.0090	0.0316	0.0091	0.0323	0.2817	0.2198	−2.4866	0.9936
SE1731	SERP1740	2.330294	835.475	346.525	835.802	346.198	836.727	345.273	2	8	0.0024	0.0231	0.0024	0.0235	0.1021	0.0927	−2.4905	0.9936
SE0262	SERP2316	2.330294	688.380	271.620	689.681	270.319	690.119	269.881	2	8	0.0029	0.0295	0.0029	0.0301	0.0963	0.0879	−2.4945	0.9937
SE0342	SERP0217	2.330294	385.601	160.399	386.052	159.948	385.827	160.173	7	12	0.0181	0.0749	0.0184	0.0789	0.2332	0.1891	−2.4996	0.9938
SE0630	SERP0522	2.330294	169.789	70.211	168.990	71.010	170.478	69.522	0	7	0.0000	0.0991	0.0000	0.1063	0.0000	0.0000	−2.5492	0.9946
SE0277	SERP2300	2.330294	455.112	177.888	456.212	176.788	455.662	177.338	4	10	0.0088	0.0564	0.0088	0.0586	0.1502	0.1306	−2.5712	0.9949
SE1009	SERP0897	2.330294	906.760	371.240	907.309	370.691	908.123	369.877	4	10	0.0044	0.0270	0.0044	0.0275	0.1600	0.1379	−2.5741	0.9950
SE0382	SERP0264	2.330294	542.773	207.227	541.923	208.077	542.348	207.652	9.5	13.5	0.0175	0.0650	0.0177	0.0680	0.2603	0.2065	−2.5777	0.9950
SE1875	SERP1883	2.330294	347.355	144.645	349.454	142.546	349.493	142.507	8	13	0.0230	0.0905	0.0233	0.0965	0.2415	0.1945	−2.5853	0.9951
SE1267	SERP1148	2.330294	1293.667	533.333	1293.367	533.633	1293.517	533.483	1	8	0.0008	0.0150	0.0008	0.0151	0.0530	0.0503	−2.6196	0.9956
SE0873	SERP0764	2.330294	368.594	156.406	368.567	156.433	368.581	156.419	3	10	0.0081	0.0639	0.0082	0.0668	0.1228	0.1093	−2.6221	0.9956
SE0004	SERP2549	2.330294	1368.051	560.949	1368.401	560.599	1369.314	559.686	0	7	0.0000	0.0125	0.0000	0.0126	0.0000	0.0000	−2.6250	0.9957
SE2193	SERP2204	2.330294	535.811	211.189	535.811	211.189	536.899	210.101	0	7	0.0000	0.0331	0.0000	0.0339	0.0000	0.0000	−2.6279	0.9957
SE0817	SERP0708	2.330294	750.975	299.025	750.625	299.375	751.888	298.112	1	8	0.0013	0.0267	0.0013	0.0272	0.0478	0.0456	−2.6464	0.9959
SE0180	SERP2395	2.330294	962.651	390.349	963.451	389.549	964.139	388.861	2	9	0.0021	0.0231	0.0021	0.0234	0.0897	0.0824	−2.6489	0.9960
SE0803	SERP0692	2.330294	959.289	399.711	960.538	398.462	961.001	397.999	2	9	0.0021	0.0226	0.0021	0.0229	0.0917	0.0840	−2.6515	0.9960
SE1874	SERP1882	2.330294	757.178	310.822	757.002	310.998	758.178	309.822	1	8	0.0013	0.0257	0.0013	0.0262	0.0496	0.0473	−2.6516	0.9960
SE0908	SERP0799	2.330294	522.664	212.336	522.664	212.336	523.752	211.248	2	9	0.0038	0.0424	0.0038	0.0436	0.0872	0.0802	−2.6629	0.9961
SE2416	SERP0004	2.330294	1322.930	552.070	1322.630	552.370	1323.868	551.132	0	7	0.0000	0.0127	0.0000	0.0128	0.0000	0.0000	−2.6667	0.9962
SE0635	SERP0527	2.330294	851.740	354.260	852.191	353.809	853.054	352.946	3	10	0.0035	0.0282	0.0035	0.0288	0.1215	0.1084	−2.6872	0.9964
SE0863	SERP0753	2.330294	480.179	185.821	480.053	185.947	481.204	184.796	6	12	0.0125	0.0646	0.0126	0.0675	0.1867	0.1573	−2.6979	0.9965
SE0844	SERP0734	2.330294	417.508	167.492	417.258	167.742	418.471	166.529	3	10	0.0072	0.0597	0.0072	0.0622	0.1158	0.1037	−2.7042	0.9966
SE1880	SERP1889	2.330294	801.624	320.376	799.975	322.025	801.888	320.112	3	10	0.0037	0.0311	0.0038	0.0318	0.1195	0.1067	−2.7088	0.9966

SE0565	SERP0450	2.330294	1709.106	666.894	1708.256	667.744	1709.769	666.231	3	10	0.0018	0.0150	0.0018	0.0151	0.1192	0.1065	−2.7126	0.9967
SE0322	SERP0199	2.330294	498.305	194.695	498.803	194.197	499.642	193.358	7	13	0.0140	0.0669	0.0142	0.0700	0.2029	0.1686	−2.7223	0.9968
SE0878	SERP0768	2.330294	779.065	318.935	777.941	320.059	779.591	318.409	4	11	0.0051	0.0344	0.0052	0.0352	0.1477	0.1287	−2.7245	0.9968
SE2187	SERP2198	2.330294	901.386	352.614	901.386	352.614	902.474	351.526	3	10	0.0033	0.0284	0.0033	0.0289	0.1142	0.1025	−2.7251	0.9968
SE0881	SERP0771	2.330294	432.502	176.498	431.652	177.348	432.077	176.923	3.5	10.5	0.0081	0.0593	0.0081	0.0618	0.1311	0.1159	−2.7267	0.9968
SE0869	SERP0760	2.330294	567.112	227.888	566.511	228.489	567.900	227.100	3	10	0.0053	0.0438	0.0053	0.0452	0.1173	0.1050	−2.7269	0.9968
SE0372	SERP0254	2.330294	547.562	226.438	546.163	227.837	547.951	226.049	4	11	0.0073	0.0484	0.0074	0.0501	0.1477	0.1287	−2.7295	0.9968
SE0914	SERP0805	2.330294	363.650	137.350	362.350	138.650	364.088	136.912	4	11	0.0110	0.0797	0.0111	0.0843	0.1317	0.1164	−2.7443	0.9970
SE0851	SERP0741	2.330294	313.318	124.682	316.120	121.880	314.719	123.281	4	11	0.0127	0.0892	0.0128	0.0950	0.1347	0.1187	−2.7862	0.9973
SE0055	SERP2501	2.330294	246.518	101.482	248.094	99.906	247.306	100.694	15	19	0.0607	0.1887	0.0632	0.2174	0.2907	0.2252	−2.7976	0.9974
SE0885	SERP0776	2.330294	444.122	176.878	444.848	176.152	445.573	175.427	1	9	0.0022	0.0510	0.0023	0.0528	0.0436	0.0417	−2.7982	0.9974
SE1511	SERP1366	2.330294	1034.286	411.714	1035.986	410.014	1036.224	409.776	1	9	0.0010	0.0219	0.0010	0.0222	0.0450	0.0431	−2.8019	0.9975
SE2162	SERP2173	2.330294	320.042	123.958	319.692	124.308	319.867	124.133	5	12	0.0156	0.0967	0.0158	0.1035	0.1527	0.1324	−2.8025	0.9975
SE2148	SERP2159	2.330294	949.209	385.791	950.209	384.791	950.797	384.203	6	13	0.0063	0.0337	0.0063	0.0345	0.1826	0.1544	−2.8081	0.9975
SE1867	SERP1875	2.330294	597.010	236.990	597.183	236.817	598.184	235.816	5	12	0.0084	0.0507	0.0084	0.0524	0.1603	0.1382	−2.8126	0.9975
SE0883	SERP0774	2.330294	295.644	109.356	295.344	109.656	296.582	108.418	1	9	0.0034	0.0822	0.0034	0.0870	0.0391	0.0376	−2.8342	0.9977
SE1879	SERP1888	2.330294	674.812	276.188	674.662	276.338	675.825	275.175	2	10	0.0030	0.0362	0.0030	0.0371	0.0809	0.0748	−2.8451	0.9978
SE0383	SERP0266	2.330294	637.973	247.027	637.496	247.504	638.822	246.178	6	13	0.0094	0.0526	0.0095	0.0545	0.1743	0.1484	−2.8545	0.9978
SE2005	SERP2018	2.330294	1278.171	548.829	1278.871	548.129	1279.609	547.391	2	10	0.0016	0.0182	0.0016	0.0185	0.0865	0.0796	−2.8628	0.9979
SE1041	SERP0930	2.330294	1779.359	740.641	1778.536	741.464	1780.036	739.964	3	11	0.0017	0.0148	0.0017	0.0150	0.1133	0.1018	−2.8852	0.9980
SE0029	SERP2468	2.330294	158.937	60.063	160.665	58.335	160.801	58.199	20.6	22.4	0.1288	0.3787	0.1413	0.5272	0.2680	0.2114	−2.8918	0.9981
SE0997	SERP0885	2.330294	1050.045	419.955	1050.144	419.856	1050.095	419.905	4	12	0.0038	0.0286	0.0038	0.0291	0.1306	0.1155	−2.9048	0.9982
SE1094	SERP0981	2.330294	575.686	231.314	576.286	230.714	575.986	231.014	4	12	0.0069	0.0519	0.0070	0.0538	0.1301	0.1151	−2.9095	0.9982
SE1076	SERP0966	2.330294	508.057	211.943	508.730	211.270	508.393	211.607	8	15	0.0157	0.0709	0.0159	0.0745	0.2134	0.1759	−2.9160	0.9982
SE0862	SERP0752	2.330294	563.587	225.413	563.736	225.264	564.750	224.250	8	15	0.0142	0.0666	0.0143	0.0697	0.2052	0.1702	−2.9203	0.9983
SE0622	SERP0516	2.330294	692.894	276.106	693.417	275.583	693.155	275.845	15	20	0.0216	0.0725	0.0220	0.0763	0.2883	0.2238	−2.9289	0.9983
SE0358	SERP0235	2.330294	537.438	209.562	537.588	209.412	537.513	209.487	2	12	0.0037	0.0573	0.0037	0.0596	0.0621	0.0585	−2.9454	0.9984
SE0696	SERP0587	2.330294	578.678	228.322	578.378	228.622	579.616	227.384	0	9	0.0000	0.0394	0.0000	0.0405	0.0000	0.0000	−2.9779	0.9985
SE0716	SERP0605	2.330294	849.868	341.132	849.868	341.132	850.956	340.044	0	9	0.0000	0.0264	0.0000	0.0269	0.0000	0.0000	−2.9889	0.9986
SE0206	SERP2371	2.330294	659.309	273.691	659.009	273.991	660.247	272.753	1	10	0.0015	0.0365	0.0015	0.0374	0.0401	0.0386	−2.9931	0.9986
SE1077	SERP0967	2.330294	510.854	209.146	510.854	209.146	511.942	208.058	2	11	0.0039	0.0526	0.0039	0.0545	0.0716	0.0668	−3.0057	0.9987
SE1866	SERP1874	2.330294	436.979	175.021	436.679	175.321	436.829	175.171	2	11	0.0046	0.0628	0.0046	0.0656	0.0701	0.0655	−3.0117	0.9987
SE0385	SERP0268	2.330294	522.438	203.562	523.216	202.784	523.915	202.085	1	10	0.0019	0.0492	0.0019	0.0509	0.0373	0.0360	−3.0225	0.9987
SE0360	SERP0237	2.330294	598.774	238.226	598.824	238.176	599.887	237.113	3	12	0.0050	0.0504	0.0050	0.0521	0.0960	0.0876	−3.0246	0.9988
SE0318	SERP0195	2.330294	756.693	317.307	756.693	317.307	757.781	316.219	2	11	0.0026	0.0347	0.0026	0.0355	0.0732	0.0682	−3.0274	0.9988
SE0196	SERP2380	2.330294	960.859	413.141	961.409	412.591	962.222	411.778	5	14	0.0052	0.0339	0.0052	0.0347	0.1499	0.1303	−3.0484	0.9988
SE0843	SERP0733	2.330294	569.582	231.418	570.055	230.945	570.907	230.093	3	12	0.0053	0.0519	0.0053	0.0538	0.0985	0.0897	−3.0530	0.9989
SE2231	SERP2263	2.330294	1417.707	547.293	1418.857	546.143	1419.371	545.629	3	12	0.0021	0.0219	0.0021	0.0223	0.0942	0.0861	−3.0560	0.9989
SE0325	SERP0202	2.330294	477.807	182.193	477.381	182.619	478.683	181.317	3	12	0.0063	0.0658	0.0063	0.0689	0.0914	0.0838	−3.0656	0.9989

SE0023	SERP2529	2.330294	344.122	132.878	345.122	131.878	345.710	131.290	0	10	0.0000	0.0755	0.0000	0.0796	0.0000	0.0000	−3.1094	0.9991
SE0341	SERP0216	2.330294	441.710	188.290	442.333	187.667	443.109	186.891	1	11	0.0023	0.0585	0.0023	0.0609	0.0378	0.0364	−3.1103	0.9991
SE0146	SERP2421	2.330294	634.160	250.840	633.460	251.540	634.898	250.102	0	10	0.0000	0.0398	0.0000	0.0409	0.0000	0.0000	−3.1221	0.9991
SE0363	SERP0240	2.330294	768.534	305.466	768.534	305.466	769.622	304.378	0	10	0.0000	0.0327	0.0000	0.0335	0.0000	0.0000	−3.1308	0.9991
SE1089	SERP0976	2.330294	813.583	314.417	812.157	315.843	813.958	314.042	9	17	0.0111	0.0539	0.0112	0.0560	0.2000	0.1667	−3.1356	0.9991
SE0281	SERP2297	2.330294	1480.609	583.391	1481.108	582.892	1481.947	582.053	15	21	0.0101	0.0360	0.0102	0.0369	0.2764	0.2166	−3.1386	0.9992
SE0389	SERP0273	2.330294	554.665	219.335	554.842	219.158	555.842	218.158	2	12	0.0036	0.0547	0.0036	0.0568	0.0634	0.0596	−3.1477	0.9992
SE0329	SERP0205	2.330294	628.061	247.939	627.362	248.638	628.800	247.200	6	15	0.0096	0.0604	0.0096	0.0630	0.1524	0.1322	−3.1496	0.9992
SE0135	SERP2430	2.330294	913.195	373.805	914.195	372.805	914.783	372.217	2	12	0.0022	0.0321	0.0022	0.0329	0.0669	0.0627	−3.1596	0.9992
SE0356	SERP0233	2.330294	318.872	131.128	319.296	130.704	320.173	129.827	1	11	0.0031	0.0840	0.0031	0.0891	0.0348	0.0336	−3.1644	0.9992
SE0830	SERP0720	2.330294	527.082	210.918	525.859	212.141	526.470	211.530	7	16	0.0133	0.0756	0.0134	0.0797	0.1681	0.1439	−3.1712	0.9992
SE0165	SERP2406	2.330294	537.244	215.756	534.170	218.830	536.795	216.205	11	19	0.0205	0.0874	0.0208	0.0930	0.2237	0.1828	−3.1856	0.9993
SE0833	SERP0723	2.330294	658.599	265.401	661.098	262.902	659.848	264.152	8	17	0.0121	0.0644	0.0122	0.0673	0.1813	0.1535	−3.1886	0.9993
SE0214	SERP2366	2.330294	1605.991	638.009	1605.991	638.009	1607.079	636.921	2	12	0.0012	0.0188	0.0012	0.0190	0.0632	0.0594	−3.1939	0.9993
SE1075	SERP0965	2.330294	624.614	257.386	624.690	257.310	624.652	257.348	10	19	0.0160	0.0738	0.0162	0.0777	0.2085	0.1725	−3.2460	0.9994
SE0367	SERP0244	2.330294	661.348	274.652	662.148	273.852	661.748	274.252	7	17	0.0106	0.0620	0.0107	0.0647	0.1654	0.1419	−3.2624	0.9994
SE0995	SERP0883	2.330294	730.375	292.625	730.924	292.076	731.738	291.262	0	11	0.0000	0.0376	0.0000	0.0386	0.0000	0.0000	−3.2712	0.9995
SE0351	SERP0228	2.330294	463.003	184.997	462.153	185.847	463.666	184.334	2	13	0.0043	0.0701	0.0043	0.0736	0.0584	0.0552	−3.3109	0.9995
SE0381	SERP0263	2.330294	456.179	176.821	455.879	177.121	457.117	175.883	4	15	0.0088	0.0848	0.0088	0.0899	0.0979	0.0892	−3.3238	0.9996
SE1734	SERP1743	2.330294	850.049	337.951	849.500	338.500	850.863	337.137	2	13	0.0024	0.0384	0.0024	0.0395	0.0608	0.0573	−3.3332	0.9996
SE0384	SERP0267	2.330294	665.790	276.210	664.214	277.786	666.090	275.910	8	18	0.0120	0.0650	0.0121	0.0680	0.1779	0.1511	−3.3351	0.9996
SE1092	SERP0979	2.330294	563.691	225.309	563.541	225.459	564.704	224.296	4	15	0.0071	0.0666	0.0071	0.0697	0.1019	0.0924	−3.3389	0.9996
SE0860	SERP0750	2.330294	996.418	395.582	996.418	395.582	997.506	394.494	3	14	0.0030	0.0354	0.0030	0.0363	0.0826	0.0763	−3.3480	0.9996
SE0278	SERP2299	2.330294	560.238	222.762	559.611	223.389	559.925	223.075	4	15	0.0071	0.0672	0.0072	0.0704	0.1023	0.0928	−3.3533	0.9996
SE0915	SERP0806	2.330294	527.436	207.564	528.587	206.413	529.100	205.900	1	13	0.0019	0.0628	0.0019	0.0656	0.0290	0.0281	−3.4070	0.9997
SE0323	SERP0200	2.330294	927.586	374.414	928.535	373.465	929.149	372.851	10	20	0.0108	0.0535	0.0109	0.0555	0.1964	0.1642	−3.4429	0.9997
SE0861	SERP0751	2.330294	840.014	341.986	840.564	341.436	841.377	340.623	1	13	0.0012	0.0380	0.0012	0.0390	0.0308	0.0299	−3.4471	0.9997
SE0005	SERP2548	2.330294	1898.033	780.967	1898.433	780.567	1899.321	779.679	1	13	0.0005	0.0167	0.0005	0.0168	0.0298	0.0289	−3.4486	0.9997
SE0627	SERP0521	2.330294	891.652	344.348	892.253	343.747	891.953	344.047	6	17	0.0067	0.0494	0.0068	0.0511	0.1331	0.1174	−3.4583	0.9997
SE0388	SERP0272	2.330294	514.932	202.068	514.133	202.867	514.533	202.467	2	14	0.0039	0.0691	0.0039	0.0725	0.0538	0.0510	−3.4648	0.9997
SE0320	SERP0197	2.330294	732.225	296.775	732.150	296.850	733.276	295.724	3	15	0.0041	0.0505	0.0041	0.0523	0.0784	0.0727	−3.4655	0.9997
SE0359	SERP0236	2.330294	693.222	293.778	692.922	294.078	694.161	292.839	2	14	0.0029	0.0476	0.0029	0.0492	0.0589	0.0557	−3.4680	0.9997
SE1067	SERP0957	2.330294	623.116	249.884	623.417	249.583	624.355	248.645	3	15	0.0048	0.0601	0.0048	0.0626	0.0767	0.0712	−3.4741	0.9997
SE0338	SERP0213	2.330294	475.601	187.399	475.702	187.298	475.652	187.348	3	15	0.0063	0.0801	0.0063	0.0847	0.0744	0.0692	−3.4860	0.9998
SE0195	SERP2381	2.330294	2157.034	857.966	2155.562	859.438	2157.386	857.614	2	14	0.0009	0.0163	0.0009	0.0165	0.0545	0.0517	−3.5014	0.9998
SE1008	SERP0896	2.330294	980.872	393.128	981.395	392.605	982.221	391.779	4	16	0.0041	0.0407	0.0041	0.0419	0.0979	0.0891	−3.5042	0.9998
SE0625	SERP0519	2.330294	871.176	340.824	872.176	339.824	872.764	339.236	3	15	0.0034	0.0441	0.0034	0.0454	0.0749	0.0697	−3.5093	0.9998
SE2188	SERP2199	2.330294	803.191	303.809	803.191	303.809	804.279	302.721	3	15	0.0037	0.0494	0.0037	0.0511	0.0724	0.0675	−3.5161	0.9998
SE0891	SERP0782	2.330294	662.662	267.338	662.911	267.089	662.787	267.213	6	18	0.0091	0.0674	0.0091	0.0706	0.1289	0.1142	−3.5267	0.9998

SE0697	SERP0588	2.330294	600.271	251.729	600.371	251.629	601.409	250.591	11	22	0.0183	0.0874	0.0186	0.0929	0.2002	0.1668	−3.5490	0.9998
SE0639	SERP0531	2.330294	828.477	323.523	827.576	324.424	828.026	323.974	6	18	0.0072	0.0556	0.0073	0.0577	0.1265	0.1123	−3.5688	0.9998
SE0621	SERP0515	2.330294	551.706	225.294	552.006	224.994	552.944	224.056	3	16	0.0054	0.0711	0.0055	0.0747	0.0736	0.0686	−3.5946	0.9998
SE1070	SERP0960	2.330294	701.926	273.074	702.227	272.773	703.165	271.835	2	15	0.0028	0.0550	0.0029	0.0571	0.0508	0.0483	−3.6053	0.9998
SE0191	SERP2384	2.330294	783.648	320.352	784.646	319.354	784.147	319.853	12.5	23.5	0.0159	0.0735	0.0161	0.0773	0.2083	0.1724	−3.6489	0.9999
SE0371	SERP0253	2.330294	851.534	333.466	851.510	333.490	852.610	332.390	12	23	0.0141	0.0690	0.0142	0.0724	0.1961	0.1640	−3.6520	0.9999
SE2161	SERP2172	2.330294	1135.632	457.368	1135.632	457.368	1135.632	457.368	6	19	0.0053	0.0415	0.0053	0.0427	0.1241	0.1104	−3.6628	0.9999
SE1066	SERP0956	2.330294	461.550	183.450	461.850	183.150	462.788	182.212	1	15	0.0022	0.0818	0.0022	0.0867	0.0254	0.0247	−3.6730	0.9999
SE0897	SERP0788	2.330294	457.911	184.089	457.034	184.966	458.561	183.439	2	16	0.0044	0.0867	0.0044	0.0921	0.0478	0.0456	−3.6999	0.9999
SE0021	SERP2531	2.330294	568.066	220.934	566.366	222.634	567.216	221.784	3	17	0.0053	0.0767	0.0053	0.0809	0.0655	0.0615	−3.7172	0.9999
SE0163	SERP2408	2.330294	343.701	148.299	343.053	148.947	343.377	148.623	13	25	0.0379	0.1682	0.0388	0.1905	0.2037	0.1692	−3.7185	0.9999
SE0542	SERP0427	2.330294	1995.186	836.814	1995.235	836.765	1996.299	835.701	1	15	0.0005	0.0179	0.0005	0.0181	0.0276	0.0269	−3.7237	0.9999
SE0877	SERP0767	2.330294	901.631	373.369	901.782	373.218	902.795	372.205	2	16	0.0022	0.0429	0.0022	0.0441	0.0499	0.0475	−3.7362	0.9999
SE0887	SERP0778	2.330294	851.252	345.748	850.952	346.048	852.190	344.810	3	17	0.0035	0.0491	0.0035	0.0508	0.0689	0.0645	−3.7365	0.9999
SE0375	SERP0257	2.330294	720.321	299.679	720.745	299.255	721.621	298.379	5	19	0.0069	0.0634	0.0070	0.0663	0.1056	0.0955	−3.7515	0.9999
SE0332	SERP0208	2.330294	1075.096	424.904	1073.622	426.378	1074.359	425.641	18	28	0.0168	0.0658	0.0169	0.0688	0.2456	0.1972	−3.7628	0.9999
SE2042	SERP2055	2.330294	1162.974	475.026	1161.324	476.676	1163.237	474.763	5	19	0.0043	0.0399	0.0043	0.0410	0.1049	0.0949	−3.7881	0.9999
SE2041	SERP2054	2.330294	1074.693	413.307	1074.644	413.356	1075.757	412.243	4	18	0.0037	0.0435	0.0037	0.0449	0.0824	0.0761	−3.7912	0.9999
SE0787	SERP0676	2.330294	1190.552	489.448	1189.852	490.148	1191.290	488.710	0	15	0.0000	0.0306	0.0000	0.0313	0.0000	0.0000	−3.8171	0.9999
SE0637	SERP0529	2.330294	930.625	383.375	929.126	384.874	929.875	384.125	7	21	0.0075	0.0547	0.0076	0.0568	0.1338	0.1180	−3.8342	0.9999
SE0245	SERP2336	2.330294	1575.543	608.457	1575.693	608.307	1575.618	608.382	6	20	0.0038	0.0329	0.0038	0.0336	0.1131	0.1016	−3.8369	0.9999
SE2147	SERP2158	2.330294	1011.527	437.473	1012.076	436.924	1012.890	436.110	3	18	0.0030	0.0412	0.0030	0.0423	0.0709	0.0662	−3.8371	0.9999
SE0337	SERP0212	2.330294	622.401	253.599	622.401	253.599	623.489	252.511	2	17	0.0032	0.0670	0.0032	0.0702	0.0456	0.0436	−3.8391	0.9999
SE0016	SERP2536	2.330294	898.488	382.512	899.465	381.535	900.065	380.935	1	16	0.0011	0.0419	0.0011	0.0431	0.0255	0.0249	−3.8689	0.9999
SE0350	SERP0227	2.330294	696.047	281.953	697.498	280.502	696.772	281.228	5	20	0.0072	0.0711	0.0072	0.0747	0.0964	0.0879	−3.8750	0.9999
SE0831	SERP0721	2.330294	753.663	302.337	753.663	302.337	754.751	301.249	4	19	0.0053	0.0628	0.0053	0.0656	0.0808	0.0748	−3.8812	0.9999
SE2220	SERP2253	2.330294	889.256	352.744	889.355	352.645	890.394	351.606	3	18	0.0034	0.0510	0.0034	0.0529	0.0643	0.0604	−3.8847	0.9999
SE0695	SERP0586	2.330294	459.126	173.874	457.577	175.423	459.440	173.560	1	17	0.0022	0.0973	0.0022	0.1043	0.0211	0.0207	−3.8981	1.0000
SE0037	SERP2498	2.330294	965.603	381.397	968.051	378.949	966.827	380.173	24	33	0.0248	0.0868	0.0252	0.0923	0.2730	0.2145	−3.8996	1.0000
SE0022	SERP2530	2.330294	583.314	214.686	582.765	215.235	584.128	213.872	0	16	0.0000	0.0744	0.0000	0.0784	0.0000	0.0000	−3.9005	1.0000
SE2192	SERP2203	2.330294	1446.970	563.030	1447.420	562.580	1448.283	561.717	3	18	0.0021	0.0320	0.0021	0.0327	0.0642	0.0603	−3.9266	1.0000
SE0698	SERP0589	2.330294	985.969	397.031	986.518	396.482	987.332	395.668	2	18	0.0020	0.0454	0.0020	0.0468	0.0427	0.0410	−3.9691	1.0000
SE2160	SERP2170	2.330294	1869.951	755.049	1871.076	753.924	1871.602	753.398	6	21	0.0032	0.0278	0.0032	0.0284	0.1127	0.1013	−3.9780	1.0000
SE0852	SERP0742	2.330294	1161.045	449.955	1163.347	447.653	1162.196	448.804	20	31	0.0172	0.0691	0.0174	0.0725	0.2400	0.1935	−4.0032	1.0000
SE0910	SERP0801	2.330294	876.786	347.214	876.437	347.563	877.700	346.300	5	21	0.0057	0.0605	0.0057	0.0630	0.0905	0.0830	−4.0241	1.0000
SE1074	SERP0964	2.330294	697.521	289.479	696.572	290.428	698.135	288.865	7	23	0.0100	0.0793	0.0101	0.0838	0.1205	0.1076	−4.0492	1.0000
SE0858	SERP0748	2.330294	949.641	397.359	948.717	398.283	950.267	396.733	6	22	0.0063	0.0553	0.0063	0.0574	0.1098	0.0989	−4.0647	1.0000
SE0632	SERP0524	2.330294	747.623	314.377	747.747	314.253	747.685	314.315	5	23	0.0067	0.0732	0.0067	0.0770	0.0870	0.0800	−4.0724	1.0000
SE1078	SERP0968	2.330294	819.413	329.587	818.513	330.487	818.963	330.037	5	22	0.0061	0.0667	0.0061	0.0698	0.0874	0.0804	−4.1527	1.0000

SE0857	SERP0747	2.330294	687.671	275.329	687.371	275.629	688.609	274.391	1	19	0.0015	0.0690	0.0015	0.0724	0.0207	0.0203	−4.1788	1.0000
SE1068	SERP0958	2.330294	626.184	276.816	625.184	277.816	626.772	276.228	4	22	0.0064	0.0793	0.0064	0.0838	0.0764	0.0710	−4.1885	1.0000
SE0854	SERP0744	2.330294	658.120	274.880	657.220	275.780	658.758	274.242	3	21	0.0046	0.0763	0.0046	0.0804	0.0572	0.0541	−4.2137	1.0000
SE0901	SERP0792	2.330294	1193.463	462.537	1191.463	464.537	1192.963	463.037	15	30	0.0126	0.0647	0.0127	0.0677	0.1876	0.1580	−4.2227	1.0000
SE1130	SERP1012	2.330294	628.415	247.585	628.265	247.735	629.428	246.572	0	19	0.0000	0.0767	0.0000	0.0809	0.0000	0.0000	−4.2579	1.0000
SE0906	SERP0797	2.330294	523.033	208.967	522.183	209.817	523.696	208.304	0	19	0.0000	0.0907	0.0000	0.0967	0.0000	0.0000	−4.2788	1.0000
SE0842	SERP0732	2.330294	603.529	236.471	604.153	235.847	604.929	235.071	1	20	0.0017	0.0847	0.0017	0.0899	0.0189	0.0186	−4.2877	1.0000
SE0362	SERP0239	2.330294	697.024	283.976	696.874	284.126	698.037	282.963	3	22	0.0043	0.0775	0.0043	0.0817	0.0526	0.0500	−4.3299	1.0000
SE1080	SERP0970	2.330294	897.206	365.794	897.529	365.471	898.455	364.545	4	23	0.0045	0.0629	0.0045	0.0657	0.0685	0.0641	−4.3487	1.0000
SE1071	SERP0961	2.330294	641.690	252.310	639.889	254.111	640.790	253.210	6	26	0.0094	0.1027	0.0094	0.1104	0.0851	0.0785	−4.5040	1.0000
SE0893	SERP0784	2.330294	783.725	308.275	783.495	308.505	784.698	307.302	2	23	0.0026	0.0746	0.0026	0.0786	0.0331	0.0320	−4.5247	1.0000
SE1065	SERP0955	2.330294	1288.831	520.169	1288.159	520.841	1288.495	520.505	15	33	0.0116	0.0634	0.0117	0.0662	0.1767	0.1502	−4.5291	1.0000
SE0624	SERP0518	2.330294	1037.945	417.055	1040.871	414.129	1039.408	415.592	10	30	0.0096	0.0722	0.0097	0.0759	0.1278	0.1133	−4.5855	1.0000
SE0836	SERP0726	2.330294	1222.830	484.170	1223.257	483.743	1224.132	482.868	12	32	0.0098	0.0661	0.0099	0.0692	0.1431	0.1252	−4.6648	1.0000
SE0892	SERP0783	2.330294	937.316	367.684	939.115	365.885	938.216	366.784	9	30	0.0096	0.0818	0.0097	0.0866	0.1120	0.1007	−4.7129	1.0000
SE0369	SERP0251	2.330294	925.015	370.985	924.391	371.609	924.703	371.297	2	25	0.0022	0.0673	0.0022	0.0705	0.0312	0.0303	−4.7502	1.0000
SE1090	SERP0977	2.330294	952.203	388.797	949.904	391.096	951.053	389.947	8.5	31.5	0.0089	0.0808	0.0090	0.0855	0.1053	0.0952	−4.8440	1.0000
SE1072	SERP0962	2.330294	1145.346	459.654	1145.097	459.903	1146.309	458.691	2	26	0.0017	0.0565	0.0017	0.0588	0.0289	0.0281	−4.8549	1.0000
SE0912	SERP0803	2.330294	981.845	383.155	982.020	382.980	983.020	381.980	1	26	0.0010	0.0679	0.0010	0.0711	0.0141	0.0139	−4.9244	1.0000
SE1099	SERP0988	2.330294	982.113	385.887	980.740	387.260	982.515	385.485	4	29	0.0041	0.0750	0.0041	0.0790	0.0519	0.0493	−4.9495	1.0000
SE0321	SERP0198	2.330294	1215.702	476.298	1215.075	476.925	1216.477	475.523	14	37	0.0115	0.0776	0.0116	0.0819	0.1416	0.1241	−4.9703	1.0000
SE1069	SERP0959	2.330294	648.728	275.272	648.702	275.298	649.803	274.197	0	26	0.0000	0.0944	0.0000	0.1009	0.0000	0.0000	−5.0199	1.0000
SE0346	SERP0220	2.330294	815.849	330.151	815.246	330.754	815.548	330.452	6	32	0.0074	0.0968	0.0074	0.1037	0.0714	0.0666	−5.0583	1.0000
SE0888	SERP0779	2.330294	1714.963	691.037	1715.790	690.210	1716.465	689.535	12	37	0.0070	0.0536	0.0070	0.0556	0.1259	0.1118	−5.1090	1.0000
SE0380	SERP0262	2.330294	1185.003	473.997	1185.329	473.671	1186.254	472.746	8	34	0.0068	0.0718	0.0068	0.0754	0.0902	0.0827	−5.1131	1.0000
SE0904	SERP0795	2.330294	695.796	279.204	696.096	278.904	697.034	277.966	2	29	0.0029	0.1039	0.0029	0.1119	0.0259	0.0253	−5.1188	1.0000
SE0856	SERP0746	2.330294	1685.205	639.795	1684.304	640.696	1684.754	640.246	12	37	0.0071	0.0578	0.0072	0.0601	0.1198	0.1070	−5.1664	1.0000
SE0832	SERP0722	2.330294	1694.652	705.348	1694.776	705.224	1694.714	705.286	19.5	44.5	0.0115	0.0631	0.0116	0.0659	0.1760	0.1497	−5.2289	1.0000
SE0884	SERP0775	2.330294	1220.748	474.252	1222.597	472.403	1222.761	472.239	4	32	0.0033	0.0676	0.0033	0.0709	0.0465	0.0445	−5.2405	1.0000
SE1091	SERP0978	2.330294	1361.776	525.224	1361.377	525.623	1362.665	524.335	9	37	0.0066	0.0704	0.0066	0.0739	0.0893	0.0820	−5.3440	1.0000
SE0841	SERP0731	2.330294	1258.950	505.050	1258.473	505.527	1259.800	504.200	1	33	0.0008	0.0653	0.0008	0.0683	0.0117	0.0116	−5.6125	1.0000
SE0909	SERP0800	2.330294	2539.242	1027.758	2540.492	1026.508	2539.867	1027.133	19.5	54.5	0.0077	0.0531	0.0077	0.0550	0.1400	0.1228	−6.1135	1.0000
SE0902	SERP0793	2.330294	1457.848	588.152	1459.147	586.853	1458.497	587.503	10.5	49.5	0.0072	0.0843	0.0072	0.0894	0.0805	0.0745	−6.1348	1.0000
SE0699	SERP0590	2.330294	1303.657	538.343	1304.407	537.593	1305.120	536.880	4	43	0.0031	0.0799	0.0031	0.0845	0.0367	0.0354	−6.1734	1.0000
SE1097	SERP0986	2.330294	2003.770	798.230	2003.520	798.480	2004.733	797.267	4	43	0.0020	0.0539	0.0020	0.0559	0.0358	0.0345	−6.2255	1.0000
SE0837	SERP0727	2.330294	1672.805	673.195	1671.554	674.446	1673.267	672.733	15	58	0.0090	0.0861	0.0090	0.0914	0.0985	0.0896	−6.5850	1.0000
SE0036	SERP2499	2.330294	1173.611	452.389	1177.382	448.618	1175.496	450.504	50	81	0.0425	0.1798	0.0438	0.2056	0.2130	0.1756	−6.6026	1.0000
SE0690	SERP0580	2.330294	1284.015	521.985	1281.967	524.033	1282.991	523.009	4	53	0.0031	0.1013	0.0031	0.1089	0.0285	0.0277	−6.7467	1.0000
SE0868	SERP0758	2.330294	1971.136	776.864	1970.486	777.514	1971.899	776.101	8	54	0.0041	0.0695	0.0041	0.0729	0.0562	0.0532	−6.7474	1.0000

SE1131	SERP1013	2.330294	2462.809	972.191	2468.585	966.415	2465.697	969.303	34	75	0.0138	0.0774	0.0139	0.0817	0.1701	0.1454	-6.8346	1.0000
SE0879	SERP0769	2.330294	2248.866	922.134	2247.067	923.933	2247.966	923.034	9	74	0.0040	0.0802	0.0040	0.0848	0.0472	0.0450	-7.9345	1.0000
