# Supplementary material for: Conserved genes in a path from commensalism to pathogenicity: comparative phylogenetic profiles of Staphylococcus epidermidis RP62A and ATCC12228
Source: BMC Genomics. 2006 May 10;7:112. doi: 10.1186/1471-2164-7-112 (PMC1482698; doi:10.1186/1471-2164-7-112)

Additional file 6 – Comparison the sensitivity to H_2O_2 of both *Staphylococcus epidermidis* strains.

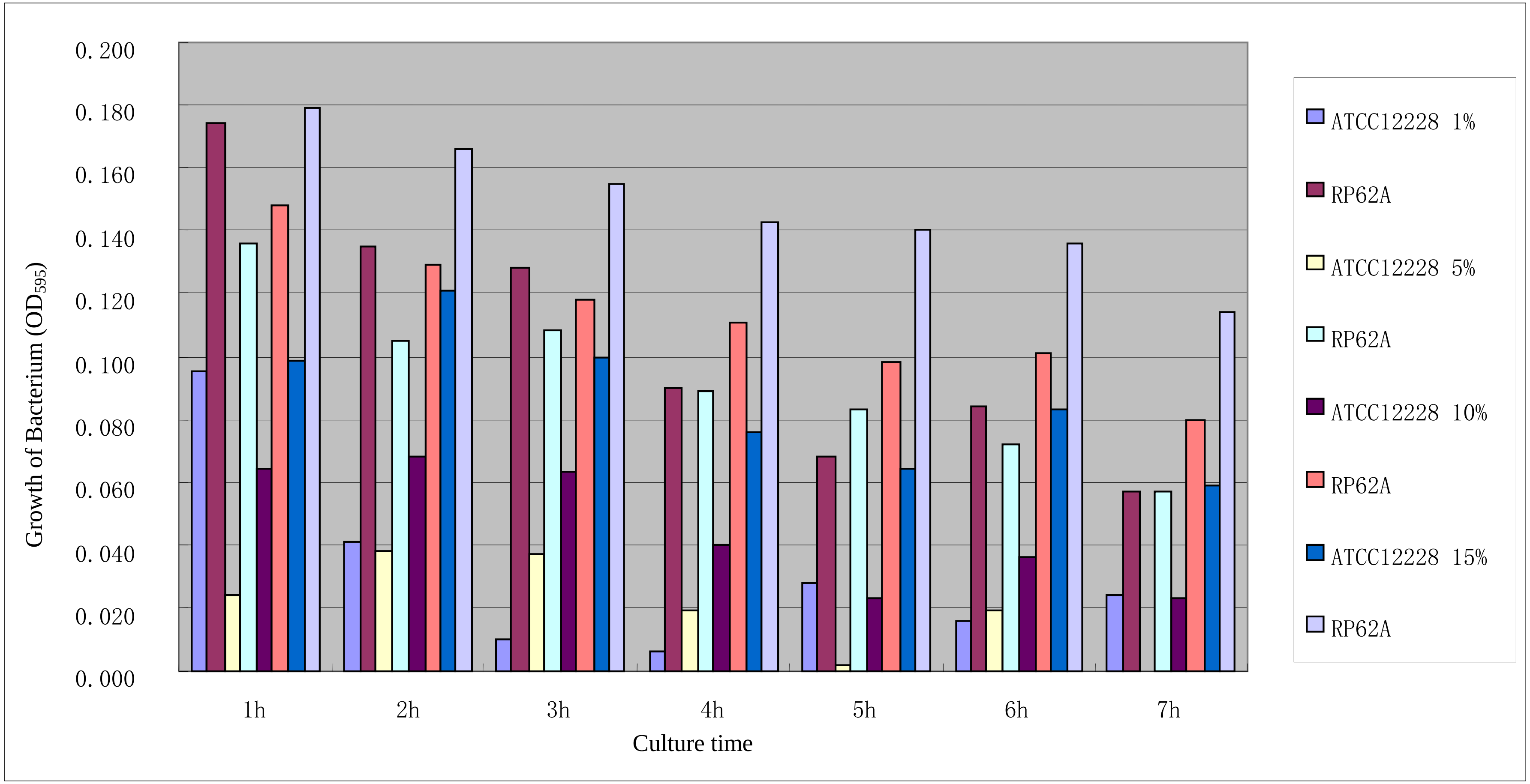

Supplement: Additional File 6 — Comparison the sensitivity to H2O2 of both Staphylococcus epidermidis strains. Each bar represents the OD595 value of one strain at specific time and concentration of H2O2. [file 1471-2164-7-112-S6.pdf]
