# Supplementary material for: Conserved genes in a path from commensalism to pathogenicity: comparative phylogenetic profiles of Staphylococcus epidermidis RP62A and ATCC12228
Source: BMC Genomics. 2006 May 10;7:112. doi: 10.1186/1471-2164-7-112 (PMC1482698; doi:10.1186/1471-2164-7-112)

Additional file 7 – Comparison the sensitivity to H_2O_2 (5%) of both *Staphylococcus epidermidis* strains.

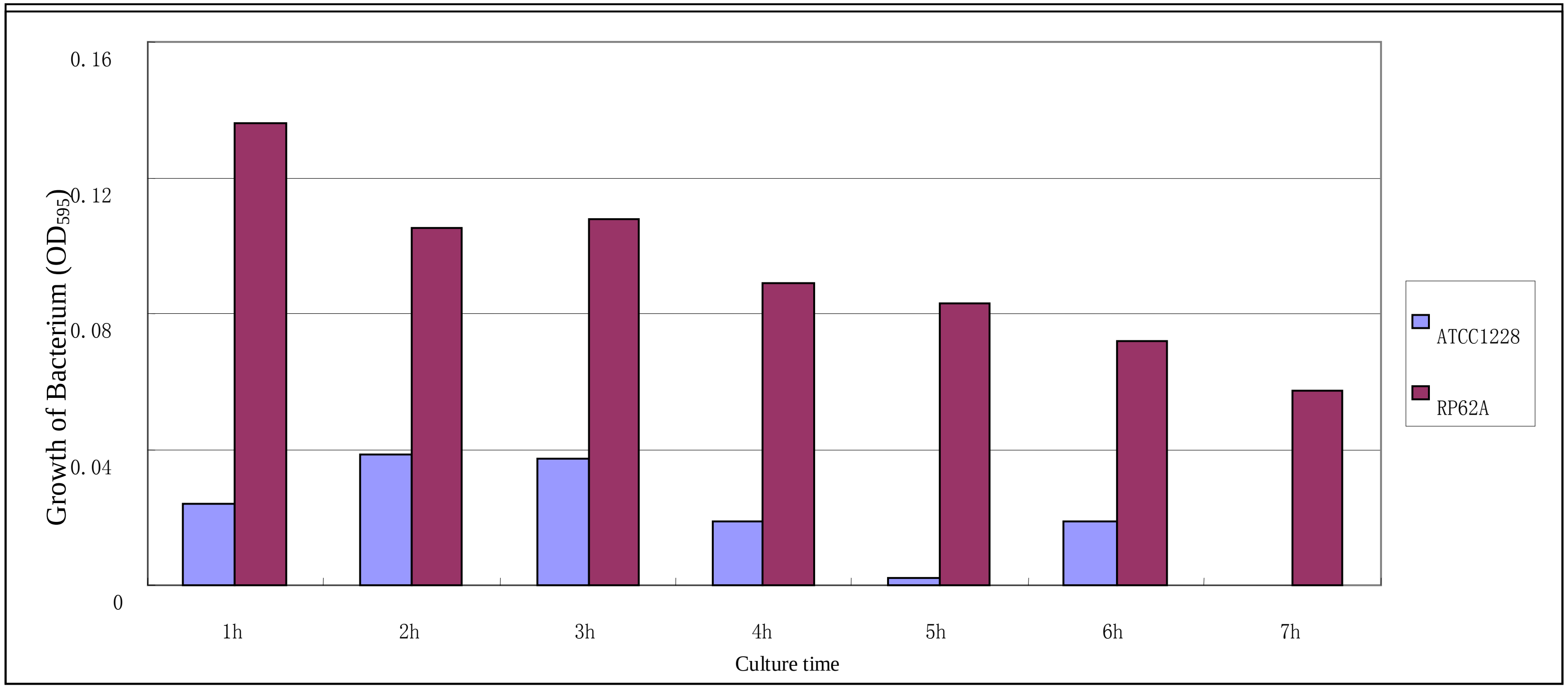

Supplement: Additional File 7 — Comparison the sensitivity to H2O2 (5%) of both Staphylococcus epidermidis strains. Red bars represent the OD595 value of S. epidermidis RP62A at different time and blue bars represent S. epidermidis ATCC12228. [file 1471-2164-7-112-S7.pdf]
