# Supplementary material for: Conserved genes in a path from commensalism to pathogenicity: comparative phylogenetic profiles of Staphylococcus epidermidis RP62A and ATCC12228
Source: BMC Genomics. 2006 May 10;7:112. doi: 10.1186/1471-2164-7-112 (PMC1482698; doi:10.1186/1471-2164-7-112)
Supplement: Additional File 9 — Comparison of identical and non-identical distribution of different functional groups of orthologous genes. [file 1471-2164-7-112-S9.pdf]

Additional file 9 – Comparison of identical and non-identical distribution of different functional groups of orthologous genes

Groups[a]	No.CDS	Non-identical[b]	Identical[b]	Ratio[b]	P_I[c]	P_{II}	P_{III}
Ⅰ	28	15	13	1.461538			
Ⅱ	12	12	0	-	0.004*		
Ⅲ	139	60	79	0.898734	0.313	0.000*	
Total	2053	1194	859	1.816065	0.544[d]	0.003[d] *	0.000[d] *

[a] Groups defines as (Ⅰ) orthologous genes whose products are virulence factors and genomic islands in two *S. epidermidis* genome; (Ⅱ) orthologous genes whose products are surface proteins; (Ⅲ) orthologous genes whose products belong to translation, ribosomal structure and biogenesis catalog according to COG.

[b] (Non-identical) number of non-identical orthologs; (Identical) number of identical orthologs; (Ratio) ratios of non-identical orthologs vs. identical orthologs.

[c] *P*-value from χ^2 test for the difference in ratios in all the pairwise comparisons among the three functional groups (* indicates $p<0.05$).

[d] *P*-value from χ^2 test for the difference in ratios between the three functional groups and total without themselves (* indicates $p<0.05$).
