# Supplementary material for: Conserved genes in a path from commensalism to pathogenicity: comparative phylogenetic profiles of Staphylococcus epidermidis RP62A and ATCC12228
Source: BMC Genomics. 2006 May 10;7:112. doi: 10.1186/1471-2164-7-112 (PMC1482698; doi:10.1186/1471-2164-7-112)
Supplement: Additional File 8 — Primers used in the PCR experiments. [file 1471-2164-7-112-S8.pdf]

Additional file 8 –Primers used in the PCR experiments.

Number		Primer		Products
ATCC12228	RP62A			
SE1029	SERP0918	Sense	5'-GGCGTTAATGCTGAACAATTT-3'	2192
		Anti	5'-TCGTTGATTCGCTTGAAAAA-3'	
SE0231	SERP2349	Sense	5'-TCCTGTGATCATTCGTTTGG-3'	760
		Anti	5'-AGGTTGGGGTCATTTTGATG-3'	
SE0800	SERP0689	Sense	5'-ACCCACTCAAATGAGAAGTTA-3'	981
		Anti	5'-ACCATTTGCTATTTTAAAGAC-3'	
SE1546	SERP1399	Sense	5'-TGAGCACTTCCTCATCAACAA-3'	846
		Anti	5'-TTTGGCAAGGAGAATATGGG-3'	
SE2085	SERP2099	Sense	5'-CTACTCTTAGCACCACCACCG-3'	1224
		Anti	5'-AGGGTGAACGATGGTGAAAG-3'	
SE1042	SERP0931	Sense	5'-TCAATTGTTGTTCCAGGCAT-3'	424
		Anti	5'-ATCGATCCTTTGGATGATGG-3'	
SE0977	SERP0866	Sense	5'-ACGTAGGGGTCTCCCTGTTT-3'	421
		Anti	5'-TTTAATGGCTGGTGCTGTTG-3'	
SE1170	SERP1049	Sense	5'-CACTGCTTAATTGGGTCTACA-3'	1422
		Anti	5'-GGAATGGTTTGGTTTTGATGA-3'	
SE0502	SERP0385	Sense	5'-CCCCAAATGAAAAAGTGGAA-3'	394
		Anti	5'-AGTCACTTTTAGGTCGCGTTC-3'	
SE0546	SERP0431	Sense	5'-TAAACGAGCCAAACAAGGC-3'	1083
		Anti	5'-GCCTCTTGGTAAGTGGTTGG-3'	
SE0265		Sense	5'-TGTTGATTTGAGGTTGCTCAT-3'	422
		Anti	5'-CGAACGGAACCTTGAACAAT-3'	
	SERP2313	Sense	5'-CGAACGGAACCTTCAACAAT-3'	
		Anti	5'-TGTTGATTTGAGGTTGCTCAT-3'	
SE0378		Sense	5'-TTTGAAGATGGGCATTTCCT-3'	470
		Anti	5'-GCATAAAACTGCTATTGCTTG-3'	
	SERP0259	Sense	5'-TGAAAAAGACAATGTTCATCA-3'	
		Anti	5'-CGAATTTCAAAAGGCGAGAG-3'	
SE0039		Sense	5'-AGATGGCAGTTTTTGTGTCG-3'	392
		Anti	5'-GGACGTTGGTTCGCATATTT-3'	
	SERP2495	Sense	5'-GGACGTTGGTTCGCATATTT-3'	
		Anti	5'-AAGATGGCAGTTTTTGTGTTG-3'	
